# Importance of work engagement in primary healthcare

**DOI:** 10.1186/s12913-022-08402-7

**Published:** 2022-08-16

**Authors:** Polona Szilvassy, Klemen Širok

**Affiliations:** 1 Community Health Centre Ljubljana, Metelkova ulica 9, 1000 Ljubljana, Slovenia; 2 Faculty of health sciences, University of Primorska, Polje 42, 6310 Izola, Slovenia

**Keywords:** Work engagement, Job resources, Primary healthcare, Job characteristics

## Abstract

**Background:**

Work engagement is crucial for quality care at the primary healthcare level. This is especially true during the Covid-19 pandemic, as it has effects on the community from both a health and economic point of view. For example, inadequate work engagement can lead to fewer referrals to the secondary healthcare level. This study aims to examine the work engagement level in a public healthcare organisation at the primary healthcare level to further explore the role of work environment characteristics. The study addresses a research gap in the field of primary healthcare and emphasises the importance of managing the factors promoting work engagement. The future of healthcare will be strongly shaped by population ageing and Covid-19 disruption, which have created unpredictable and unfavourable working situations.

**Method:**

A descriptive, cross-sectional, correlational design was used including the Utrecht Work Engagement Scale with a non-probabilistic availability sample of 630 employees of the Community Health Centre Ljubljana, Slovenia, in 2018. The role of the work environment was observed by applying the job resources concept adapted to the context of the observed organisation.

**Results:**

Work engagement in the observed organisation is higher compared to previous research. The research confirmed that job resources play an important role in employees’ work engagement. The high level of work engagement of the home care nursing employees coupled with the significant proportions of unengaged in the management of the organization also caught our attention. This difference highlights the importance of the leadership style, career choices and employment process that exist in an institution.

**Conclusion:**

The study has important implications for healthcare management at the primary level for unlocking the work engagement by ‘managing’ the factors stimulating work engagement. The hidden potential is especially large in so called ‘soft areas’, such as leadership style, communication and organisational climate, which are also less expensive to manage than other aspects of the work environment.

**Supplementary Information:**

The online version contains supplementary material available at 10.1186/s12913-022-08402-7.

## Background

There are several important reasons why an engaged workforce is essential in 21st-century healthcare: (1) an increased need for healthcare services due to population ageing, and consequently, (2) a higher demand for healthcare workers, (3) who are also ageing. The ageing population and societal factors such as urbanisation and sedentary lifestyles have increased the number of people living with chronic conditions and multimorbidities. This will continue to drive strong demand for a variety of healthcare services [1] and in turn cause shortages of healthcare workers [2–4]. Recent labour force projections [5, 6] suggest that the number of occupational categories (e.g. healthcare workers) is expected to grow significantly, and a global healthcare worker shortage is predicted to occur within the next 10–20 years. This is already evident in Slovenia, which is witnessing critical shortages of nurses [7] and family medicine specialists [8]. Further, like the population they serve, healthcare workers are also ageing, with a growing share reaching retirement age and facing working limitations brought about by the ageing process.

The above-mentioned developments put healthcare workers and healthcare systems under pressure, resulting in numerous challenges: employee retention, attracting the new workers, burnout prevention and maintenance and/or improvement of service quality. Research [9, 10] has also shown that healthcare workers are inclined to search for work abroad due to better conditions, supported by increased mobility driven by rising East–West and South–North intra-European migration, especially within the European Union—as is also the case in Slovenia. Failure to address these challenges will inevitably affect the healthcare system, as it is unlikely that simply training more healthcare workers will help to avoid a shortage given the continuing reduction in healthcare costs and increasing prevalence of expatriate healthcare staff [4].

Work engagement is one of the key factors that can address those challenges. According to Schaufeli and Bakker [11] work engagement is defined as a positive, fulfilling, work-related state of mind that is characterized by vigor, dedication, and absorption. Vigor is characterized by high levels of energy and mental resilience while working. Dedication refers to being strongly involved in one’s work, and experiencing a sense of significance and enthusiasm. Absorption is characterized by being fully concentrated and happily engrossed in one’s work. In an increasingly competitive and challenging healthcare environment characterised by increasing work demands and limited resources, providing a working environment that generates positive work attitudes and behaviours is critical [12]. Research has demonstrated that work engagement mediates the relationship between work environment and turnover intention [13–15]. Work engagement also plays an important role in burnout prevention [16], which is particularly significant under the strenuous conditions that modern healthcare is facing. The consequences of burnout can be severe for both the healthcare professional and the patient and, consequently, for the healthcare organisation [17, 18]. Work engagement is also a key factor in providing exemplary healthcare services. Engaged healthcare employees deliver high-quality, cost-effective care and pursue activities beyond their formal job descriptions [19, 20]. Work engagement may be associated with mandatory in-role behaviours and discretionary organisational citizenship behaviours (OCBs) at work [19, 21]. OCBs are particularly important for front-line healthcare workers because they have the most frequent interactions with patients, making them crucial for high quality healthcare services [22].

Importance of engaged and flexible healthcare staff was accentuated during the Covid-19 pandemic. López-Cabarcos et al. [23] report that nurses with higher engagement experienced less burnout facing high job demands during the Covid-19 pandemic. The research among front line nurses in China [24] revealed that that psychological resilience was significantly correlated with the overall nurse work engagement in various dimensions. In addition, as was the case in Slovenia, the requirement for functional flexibility of other healthcare employees to help to deal with crisis situations related to Covid-19 testing, mass vaccination and pulmonology emergencies during the pandemic peak also confirmed that an engaged workforce is positively related to two performance factors: adaptivity and proactivity [25].

There are numerous factors associated with employee engagement and the healthcare setting at both the organisational and individual levels that organisations can manage to improve employee work engagement levels. In their meta-analytic review Lesener et al. [26] categorised the drivers of work engagement into group level, leader level, and organisational level job resources and established the general positive impact of job resources on work engagement. More specifically, Van Bogaert et al. [27] list several characteristics of the (nurse) work environment that influence work engagement: job complexity, role ambiguity, high responsibility, mental and physical workload, lack of job control, span of control and workload, lack of opportunities for intellectual and professional growth, inadequate leadership, deficient social support by supervisor and/or colleagues, difficult nurse–doctor collaboration and effort–reward imbalance.

Nevertheless, studies on work engagement in (primary) healthcare are limited despite the importance of healthcare worker engagement [28]. Empirical research on work engagement in primary healthcare (PHC) is scarce [29–31]. An in-depth literature review only uncovered three empirical studies on work engagement at the PHC level [32–34]. In addition, the studies on work engagement have reported inconsistent results, which could be related to the use of different measuring instruments. Moreover, healthcare research results show that the majority of employees do not have an optimal engagement level, especially in professions that require significant dedication and energy. Gallup [35] identified only 33% engaged health professionals compared to 52% non-engaged and 15% actively unengaged. The consulting firm Towers Watson [34] found that 34% of employees in hospitals and other healthcare organisations are highly engaged while 42% are partly engaged, with higher scores on the rational and motivational dimensions but lower on the emotional dimension. The remaining 24% are either partly or fully unengaged, meaning they are completely disconnected on all three dimensions. These figures have changed little in the past few years. Moreover, Aboshaiqah et al. [13] observe that low levels of work engagement have been reported among nurses in comparison with other healthcare workers.

## Methods

### Aim

This study addresses a research gap in the field of PHC and employee engagement, despite the fact that dedicated staff is key for ensuring the quality of care. Effective PHC functioning is crucial for ensuring quality healthcare and the health of the population in general [36], as PHC is the cornerstone of health systems and a cost-effective way to provide universal health coverage. Therefore, this study seeks to understand work engagement within a PHC organisation in relation to various job resources, as empirical evidence evidently suggests that job resources at each of the three levels (group level, leader level, and organisational level) predict work engagement over time [26].

### Study design and participants

Study used a cross-sectional, descriptive, correlational design. The research was carried out in May 2018 in the largest PHC centre in Slovenia (Community Health Centre Ljubljana; CHCL). The study included 1554 employees working in nine organisational units at different locations in Ljubljana, the capital city of Slovenia. CHCL provides more than 20 different healthcare activities, such as family medicine infirmaries, pre-school, school and teen healthcare, dental care, laboratory diagnostics, x-ray diagnostics and a mental health centre. The surveying resulted in a non-probabilistic availability sample (Additional file 1) of 630 complete responses (90.5%) and 96 partial responses (9.5%). The majority of the respondents — almost half (46%) — were employed as nurses. A quarter (25%) of the respondents were doctors and dentists. There were fewer other healthcare and non-healthcare workers (healthcare workers and associates: 17%, non-healthcare workers: 10%, management: 2%).

A comparison of the population and respondents demonstrated that the sample adequately reflected the structure of the population (Table 1). The healthcare services representation, professional role and managerial position structures were comparable. There were some differences in service and educational structures, with those in female healthcare services and with an undergraduate education level being overrepresented and those who attained professional secondary education (4 years) and in specialist services being under-represented.

**Table 1:** Comparison of personnel structure with respondents’ structure

Demographics	Population *N* = 1554	% of the population	Respondents *n* = 630	% of the respondents
Healthcare Services				
Family medicine infirmaries	508	33	193	31
Dental care	207	13	89	14
Health visiting and home nursing	117	8	62	10
Specialist services	205	13	53	8
Female healthcare services	36	2	14	2
Other services	481	31	219	35
Occupational group				
Doctors and dentists	452	29	160	25
Nurses	702	45	290	46
Healthcare professionals and associates	262	17	104	17
Non-healthcare professionals	124	8	64	10
Top management	14	1	12	2
Educational groups				
Vocational secondary education (3 years)	25	2	16	3
Professional secondary education (4 years)	579	37	177	28
Higher vocational education	73	5	57	9
Undergraduate 1st cycle	407	26	233	37
Master’s degree	420	27	126	20
MSc	36	2	17	3
PhD	14	1	4	1
Managerial tasks				
Non-manager	1395	90	531	84
Manager	159	10	99	16

### Data collection

Data were collected through a self-report web survey targeting the whole accessible population of CHCL, whereby all employees received an invitation email with a link to the web survey, which was set up in LimeSurvey. In the email, the objective of the research was stated as well as the total confidentiality and voluntary nature of participation. During the data collection period, a reminder was sent to non-respondents after one week. The guidelines and ethical principles of the Declaration of Helsinki were followed accordingly throughout the research process [37].

### Measures

Based on different concepts of work engagement, researchers have developed several instruments for applied research in organisations as well as for scientific purposes. First, a distinction needs to be made between questionnaires that assess work engagement as a separate concept and questionnaires that assess engagement as the opposite of burnout [11]. Second, engagement questionnaires are most appropriate for measuring the emotions of engaged employees or the state of engagement. Since behavioural engagement leads directly to work outcomes and is a predictor of job performance [25], the 17-item version of the Utrecht Work Engagement Scale (UWES) [38] was chosen. The official version of the questionnaire [39] was used as a basis and was back-translated from English to Slovene. The tool consists of 17 items that measure the three components of work engagement: absorption (6 items, e.g., ‘Time flies when I'm working’), dedication (5 items, e.g., ‘I find the work that I do full of meaning and purpose’) and vigour (6 items, e.g., ‘At my work, I feel bursting with energy’). Each item is rated on a seven-point Likert scale ranging from ‘never’ to ‘always’ and scored from 0 to 6, with a higher score indicating higher work engagement. The results of the UWES are divided into five categories: very low, low, average, high and very high level of engagement. Based on the guidelines of the questionnaire designers [39], the following score norms were used to interpret the levels of work engagement: very low ≤ 1.93; low 1.94–3.06; average 3.07–4.66; high 4.67–5.53, very high ≥ 5.54. The original UWES, which has been widely used, showed evidence of convergent and divergent validity and high reliability equal to or exceeding 0.90 [18, 40]. In our study, the alpha reliability of the scale was assessed (α = 0.951), and it did not require the exclusion of any items.

The second part of the questionnaire addressed job resources, as work engagement may also be influenced by factors, such as work experience [41], peer relations and support, good leadership and communication [40, 42] and supervisor social support [43]. Following the classification of job resources [26], we observed respondents' satisfaction with the quality of group-level resources (co-workers’ relationships, communication), leader-level resources (supervisor relationship, organisational leadership) and organisational-level resources (availability of material resources, learning opportunities, personal and professional growth). The job resources items were developed taking into account existing questionnaires and adapted to the context of the observed organisation. A five-point Likert scale was used, ranging from ‘strongly disagree’ to ‘strongly agree’. In addition, respondents were asked to indicate their departmental affiliation (i.e. healthcare activity), occupational group, hierarchical position and education attained.

### Statistical analysis

Using SPSS version 26.0, the collected data were examined using univariate descriptive statistics. Cronbach’s alpha coefficient was used to assess the internal consistency of the questionnaire. The Student’s t-test was used to compare two independent groups, and for multiple groups comparisons a one-way analysis of variance (ANOVA) was used. To determine statistical differences, Levene’s test for the homogeneity of variances was applied and then, if required, the nonparametric Welch test and the Games–Howell test for post-hoc comparisons. In order to assess the relationship between workplace and organisational unit characteristics and employee work engagement, a general regression procedure [44] was applied by first exploring the correlations, presenting the regression model and model parameters and checking for bias and assumptions of the linear models. The level of statistical significance was set at *p* < 0.05.

### Ethical considerations

Although the research had no direct impact on humans and the research methods were non-invasive, the study was approved by the institutional review board—the Primary Healthcare Research and Development Institute. All ethical principles of research were followed and there was no penalty for withdrawing or stopping the study. Written informed consent was obtained from all participants before the study.

## Results

The research showed that CHCL employees are engaged, and more than half of them are highly or very highly engaged. The total score shows the average work engagement ($\overline{x }$=4.65; SD = 0.95), which is very close to the lower limit of the high work engagement level according to the UWES norm. Based on the dimension scores (Table 2), vigor is average ($\overline{x }$=4.56) as is dedication ($\overline{x }$=4.82), while absorption is high ($\overline{x }$=4.60). The share of very highly engaged employees in the total observed sample (*n* = 630) is 15.7%, and the share of highly engaged employees is 41.1%. In contrast, 5.4% employees have low and 1.1% employees have a very low work engagement level. The ratio between engaged and other employees is 1.3:1, and the ratio between engaged and actively unengaged employees is 8.7:1.

**Table 2:** Work engagement – descriptive statistics

	Vigor	Dedication	Absorption	Total engagement score
n	630	630	630	630
mean	4.56	4.82	4.60	4.65
standard deviation	1.002	1.015	0.99	0.95
qualification according to UWES norms^a^	average	average	high	average

^a^UWES score norms: Very low ≤ 1.93; Low 1.94 – 3.06; Average 3.07 – 4.66; High 4.67 – 5.53, Very high ≥ 5.54

An overview of the job resources shows that the respondents generally agree that they are properly taken care of. Employees perceive that they have adequate material resources to do their job well ($\overline{x }$=3.21; SD = 1.060) and that the organisation enables personal growth ($\overline{x }$=3.34; SD = 1.077). They also strongly agree that they have good relationships with their co-workers ($\overline{x }$=4.06; SD = 0.881) as well as with their supervisor ($\overline{x }$=4.02; SD = 0.967). The organisation management is perceived as good ($\overline{x }$=3.52; SD = 1.003). Employees have the information they need to do a quality job ($\overline{x }$=3.52; SD = 0.948) and enough learning opportunities for professional development in the organisation ($\overline{x }$=3.55; SD = 1.071).

CHCL employees working in different healthcare activities grouped according to the specifics of work and patients exhibit different work engagement levels. Highly engaged employees are involved in the activities of health visiting and home nursing and dental care, and these two activities also have the highest share of engaged employees. In other activities, including family medicine infirmaries, specialist services, female healthcare services and other services, employees have an average work engagement level (Table 3). Levene’s test for homogeneity of variance (*p* < 0.05) indicated that a nonparametric Welch test should be performed, which showed that there were statistically significant differences (F(5, 85.05) = 6,745; *p* = 0.000) between health visiting and home nursing, which have higher levels of work engagement than family medicine infirmaries (0.621 higher engagement level, *p* = 0.000) and other services (0.375 higher engagement level, *p* = 0.004).

**Table 3:** Work engagement mean scores according to the activities

Activities	Mean	SD
Health visiting and home nursing*	5.08	0.660
Dental care	4.82	1.004
Family medicine infirmaries*	4.46	0.939
Specialist services	4.88	0.980
Female healthcare services	4.31	1.301
Other services*	4.71	0.846

^*^statistically significant differences (*p* < 0.05)

The findings indicate that highly engaged employees come from three occupational groups: doctors and dentists, healthcare workers and associates and top management (Table 4). Top management includes the positions of general manager, medical director, deputy director and heads of sectors and organisational units. The other two occupational groups, nurses and non-healthcare workers, exhibit an average work engagement level. The ANOVA showed that there were no statistically significant differences in work engagement by occupational group (F(4, 609) = 0.730, *p* = 0.572). Although top management has a high level of work engagement, they also have a significant proportion of employees with an average engagement level (41.7%).

**Table 4:** Work engagement mean scores according to the occupational groups

Occupational group	Mean	SD
Doctors and dentists	4.710	0.882
Healthcare workers and associates	4.670	0.865
Management	4.850	0.610
Nurses	4.658	0.985
Non-healthcare workers	4.477	1.026

Those who perform managerial tasks in top and middle management positions ($\overline{x }$=4.94; SD = 0.616) are significantly (t(628) = 23,571; *p* = 0,000) more engaged than non-managers ($\overline{x }$=4.60; S = 0.992). Individuals performing managerial duties (of 159 employees in CHCL, 14 occupy top management positions and 145 occupy middle management positions) exhibit a high work engagement level, while non-managers exhibit average work engagement. However, while nearly three-quarters of managers have a very high or high engagement level, a quarter of them have an average and none of them have a low or very low work engagement level.

The findings indicate that work engagement levels are statistically significantly lower for individuals with professional secondary education (4 years). Four educational groups have a high work engagement: employees with a PhD ($\overline{x }$=5.29; SD = 0.468), MSc ($\overline{x }$=4.86; SD = 0.665), undergraduate first cycle ($\overline{x }$=4.78; SD = 0.823) and higher vocational education ($\overline{x }$=4.72; SD = 0.812). The remaining three educational groups have an average work engagement level: employees with master’s degree ($\overline{x }$=4.65; SD = 0.878), professional secondary education (4 years) ($\overline{x }$=4.45; SD = 1.103) and vocational secondary education (3 years) ($\overline{x }$=4.80; SD = 1.271). Engagement increases almost linearly with a higher level of education. Levene’s test for homogeneity of variance indicated the use of the nonparametric Welch test, which uncovered statistically significant differences between the educational level achieved and work engagement (F(6, 32.727) = 2.743; *p* = 0.028). Furthermore, the Games–Howell post hoc procedure showed that employees with secondary education (4 years) exhibit a 0.310 (p = 0.028) lower level of engagement compared to those with undergraduate first cycle education.

The initial regression model with all job characteristics predictors exhibited some problems. Two predictors, organisational leadership (t(7) = 1.32, *p* < 0.187) and learning opportunity (t(7) = -0.79, *p* < 0.459), proved to be statistically insignificant, and learning opportunity shoved signs of possible collinearity with personal and professional growth (*r* = 0.816, *p* = 0.000). After organisational leadership and learning opportunity were excluded from the final model, the improved model had an R^2^ value only 0.002 lower than the initial model, with a significantly higher F-ratio. All correlations of the final model are positive, moderate in size and significant at *p* = 0.001. None of the correlations between two predictor variables is above 0.80, indicating that our predictors are measuring different things, that is, there is no collinearity. The absence of collinearity was also confirmed by the variance inflation factor (model average VIF is 2.03, largest VIF is 2.40) and tolerance statistics (1/VIF ranges between 0.43 and 0.67). In our model, each predictor has most of its variance loading onto a different dimension. Only personal and professional growth have two rather large loadings over two dimensions (0.84 and 0.67). We also checked the data for evidence of bias by examining the case-wise diagnostics and linearity assumptions. Our sample appears to just conform to what one would expect for a fairly accurate model, as 29 cases (4.8%) fell outside the criterion that 95% of cases should have standardised residuals within about ± 2 [45]. In our sample, there are four cases with a standardised residual greater than 3, which should be investigated further. None of them have a Cook’s distance greater than 1, so none of the cases has an undue influence on the model. Heteroscedasticity and non-linearity were checked using a plot of standardised residuals against standardised predicted values. The histogram and normal probability plot indicate the normality of the residuals. Regarding the employee engagement data, the distribution is very normal: the histogram is approximately bell-shaped and symmetrical. The P-P plot shows small deviations from normality as deviations from the diagonal line, and thus this plot also suggests that the residuals are normally distributed.

We can define our specific regression model as follows:

$$\mathrm{engagementi }= b0 + b1\mathrm{ material resourcesi }+ b2\mathrm{ co}-\mathrm{workers relationshipi }+ b3\mathrm{ supervisor relationshipi }+ b5\mathrm{ communicationi }+ b7\mathrm{ personal and professional growthi }= 1.87 + (0.17\mathrm{ material resources}) + (0.15\mathrm{ co}-\mathrm{workers relationship}) + (0.14\mathrm{ supervisor relationship}) + (0.15\mathrm{ communication}) + (0.16\mathrm{ personal and professional growth})$$

All b-values values (see Table 5) are positive and similar in size, meaning that an increased perception of the presence of the specific job resources listed below by one unit on the Likert scale affects the level of engagement if the effects of all other predictors are held constant. For instance, as the perception that the physical work environment provides the worker with the necessary means increased by one unit, employee engagement increased by 0.16 units if other predictors were held constant.^1^ In the model, all predictors have very tight confidence intervals not crossing zero, indicating that the estimates for the current model are likely to be representative of the true population values.

**Table 5:** Regression coefficients

	b	SE b	B	p
(Constant)	1.87	0.15		0.000
material resources	0.17	0.03	.19	0.000
co-workers relationship	0.15	0.05	.14	0.002
supervisor relationship	0.14	0.05	.15	0.002
communication	0.15	0.05	.15	0.002
personal and professional growth	0.16	0.04	.18	0.000

The R^2^ value, which measures how much the variability in the outcome is accounted for by the predictors, is 0.388, which means that the selected job resources account for 39% of the variation in employee engagement. Comparison of adjusted R^2^ to R^2^ yields a small difference, meaning that if the model were derived from the population rather than a sample it would account for approximately 0.5% less variance in the outcome. For the model, the F-ratio is 79.206 and *p* < 0.000, indicating that the proposed regression model significantly improved our ability to predict the outcome variable compared to not fitting the model. The Durbin–Watson test statistic of 2.026 indicates that the assumption of independent errors is tenable. As a conservative rule, Field [45] suggests that values less than 1 or greater than 3 should raise an alarm; the closer to value is to 2, the better.

## Discussion

The majority of employees in CHCL are highly or very highly engaged. Only a small proportion of employees exhibit low or very low work engagement. Although direct comparison of UWES and Gallup Q12 instruments is considered problematic [46], the comparison results considering the UWES score norms and Gallup Q12 engagement index presented in Table 6 below clearly demonstrate that CHCL employees seem to be more engaged in work when compared to the institutions observed in other research in Slovenia and abroad.

**Table 6:** Comparison of employee work engagement levels with other surveys

Research (research setting, work engagement instrument)	GALLUP					Ratio
	**Engaged (%)**		**Unengaged (%)**	**Active unengaged (%)**		
	**UWES**					
	**Very high (%)**	**High (%)**	**Average (%)**	**Low (%)**	**Very low (%)**	**Engaged: active unengaged**
World average Gallup [35] (142 countries worldwide) Q12	13		63	24		0.5:1
Slovenian average Gallup [35] Q12	15		70	16		0.9:1
**Field of healthcare abroad**						
Gallup [35] Q12	33		52	15		2.2:1
Towers Watson [34] (hospitals and other healthcare-related organisations)	34		42	24		1.4:1
Canada, Ontario [47] (16 hospitals – Employee Experience Survey)	29		39	33		0.9:1
India [48] (35 hospitals – Employee Engagement questionnaire combined with a customer perception questionnaire)	11		no data	0		-
**Field of healthcare in Slovenia**						
CHCL (2018) (PHC – UWES-17)	16	41	37	5	1	8.7:1
Organisational unit Patronage care CHC Maribor [31] (PHC – Gallup Q12)	44		54	2		22:1
KOVNINT [49] (Hospital – Gallup Q12)	35		51	14		2.5:1
ZZV Novo mesto [50] (other health org. – Gallup Q12)	72		24	4		20:1

The research confirmed that job resources play an important role in employees’ work engagement. In Slovenia, CHCL is recognised as one of the most successful PHC organisations, which offers its employees good working conditions and enough opportunities for learning and professional growth (spreading mediation skills, through various interpersonal communication workshops and quality weeks, managing personal education and career development plans). This is in line with Wang and Liu [51], who found that a supportive work environment and sufficient resources were more likely to engage nurses in their work. Van Bogaert et al. [27] found that nurse management, nurse–physician relations and workload predicted work engagement.

Healthcare service differences in work engagement indicate the important role of management style and career choices and the employment process that is in place in an institution. Significantly higher work engagement in the health visiting and home nursing service can be explained by the fact that the work in this unit is performed by community nurses, including the head of the service, and specific work tasks do not differ significantly depending on hierarchical position. In addition to the patients’ medical treatment in the field, the head of service has few additional managerial duties. For subordinates, this results in a positive perception and additional motivation, especially in the case where the manager performs the same work as the employee and leads by example. Another hypothetical explanation is that community nurses enjoy their job, have higher OCB levels and are therefore very dedicated. The daily experience in CHCL is in line with the findings of Sacks et al. [52] and indicates that not all mid-level nurses or graduate nurses prefer daily field work, and those career choices are heavily driven by a strong intrinsic motivation to provide help to and work with difficult patients.

No statistical differences were found when comparing the work engagement of occupational groups. The observed engagement levels are in line with the existing body of evidence. The average work engagement level for nurses is 4.66, which is just below the high engagement threshold, meaning that nurses in CHCL are only slightly less engaged than doctors and other healthcare workers and associates. However, it should be kept in mind that nurses in health visiting and home nursing as a group are much more engaged than nurses from other organisational units. A total of 56.9% of nurses working in CHCL are engaged, 34.5% are unengaged and 8.6% are actively unengaged, comparable to findings of Milojevič [49], who found 35% of nurses are engaged, 51% are unengaged and 14% are actively unengaged. In CHCL, 77.4% of employees in the health visiting and home nursing service are engaged, 21.0% are unengaged and 1.6% are actively unengaged, which is also much better than the findings from the Organizational Unit Patronage Care in Community Health Centre Maribor, in which 44% of employed nurses are engaged, 54% are unengaged and 2% are actively unengaged [31]. A cross-sectional study conducted on Spanish nurses to assess the work engagement level of Spanish nurses during the Covid-19 pandemic found a high level of work engagement in all dimensions in general, and the nurses in PHC are even more engaged than nurses in hospitals [53]. A Gallup study for India also reported that 81% of nurses and 82% of physicians are engaged in work, and none of the respondents reported being actively unengaged [35]. In this research, there are fewer engaged nurses (56.9%) and doctors and dentists (60.1%).

Regarding the managerial position, research found that those in managerial positions are significantly more engaged than those who do not occupy managerial function. Horvat [31] reported similar findings in research on the Organizational Unit Patronage Care CHC Maribor as did the study in Finland [54]. Those in middle management are slightly more engaged than those in top management, although both have a high work engagement level. It was also found that more than a quarter of managers are unengaged. The relatively high proportion of non-engaged management personnel is surprising, as many studies have confirmed a positive correlation between perceived supervisor support and subordinate work engagement [50, 55–57]. Similar findings have been reported in studies conducted in healthcare environments [48, 53, 58]. We hypothesize that this could be due to excessive workload, work-related stress or disillusionment. The education level differences in terms of significantly lower levels of work engagement for those with professional secondary education (4 years) can be hypothetically explained by the job and task structure within (public) the health sector's job classification. Lower paid jobs of lesser quality are usually occupied by lower educated and less skilled workers and—certainly in the healthcare sector—also have lower occupational prestige. Similar results were also observed by Fink, Bauer and Bošković [57], indicating that highly educated employees have higher work engagement level.

The regression results further confirm the important role of job resources in increased work engagement. When excluding organisational leadership and learning opportunity from the model (which not proved to be statistically significant), the availability of material resources, quality of co-workers and supervisor relationship and communication as well as the opportunity for personal and professional growth account for 38% of the variation in employee work engagement. These findings are in line with those of existing research [27, 51].

There are some study limitations that must be acknowledged. First, self-reported questionnaires were used with an inherent assumption of the trustworthiness of the respondents. In addition, common method variance could bias the results. Further, despite the reasonably large sample, the data came from one PHC institution located in a highly urbanised and culturally (relatively) homogeneous environment. The study also used a cross-sectional design, limiting assertions about cause–effect relationships. Common method bias was addressed by using measures with well-established construct validity and internal reliability and a questionnaire design with separate dependent, independent and criterion variables.

## Conclusion

The study makes multiple contributions to the existing literature and opens new lines of research. Our study clearly demonstrated that employees in CHCL are engaged and that the job resources play an important role in work engagement.

1. Regardless of limitations, this study is the first large-scale study to examine the attributes of the PHC work environment on employee work engagement. The impact of role, job and organisational characteristics on nurse managers’ work engagement has been well researched [27] but to a lesser extent in the PHC or with PHC workers. The observed differences in work engagement levels from other studies in Slovenia and abroad indicate the need for future research to explore the role of different cultural and service characteristics that might impact work engagement.
2. Additionally, the specific job resource correlating with work engagement in an organisational setting has been explored for the first time: that is, the availability of material resources required for work. Research has also confirmed the positive relationship between the job resources and employee work engagement, including the (unclear) role of leadership and the importance of ‘soft factors’, such as the quality of co-worker and supervisor relationships and communication. However, more research is needed considering other aspects besides flexibility and the high number of different situations derived from the new work contexts. Future research in other cultural environments should attempt to replicate the findings of this study, ideally with regard to outcomes, using methods other than self-report. In addition, future research should also examine the role of work engagement in other job-related attitudes, including job satisfaction and organisational commitment.

The study has important implications for healthcare management (at the PHC level) in terms of improving work engagement by ‘managing’ the factors stimulating work engagement. The hidden potential is especially large in so-called ‘soft areas’, such as leadership style, communication and organisational climate, which are also less expensive to address than other dimensions of work environment. Career management and the employment process are other areas that deserve attention in this respect. They might—according to the existing body of evidence concerning the role of work engagement on retention, productivity, burnout prevention, required functional flexibility, shortages of healthcare workers and service quality—prove decisive for the future of healthcare, which is being strongly shaped by population ageing and Covid-19, both of which have created extremely unpredictable and unfavourable situations. Healthcare organisations need to be as flexible as possible and ready for rapid change, and effective collaboration between healthcare teams including timely conflict resolution will be crucial for managing this situation successfully and effectively.

Finally, despite its key importance to ensuring social welfare, PHC has not received adequate research attention, at least in the area of employee work engagement. First, patient healthcare begins at the PHC level, where the majority of healthcare services are provided. Second, at least in Slovenia, one-third of healthcare workers are employed at the PHC level, which is certainly not negligible. Third, quality care requires an adequate supply of engaged workers. In PHC, work engagement is crucial, especially during the Covid-19 pandemic, as poor work engagement can negatively affect the community from both a health and economic point of view (fewer referrals to a secondary level of care or in hospitals).

## Supplementary Information

**Additional file 1.** 

## Data Availability

The dataset supporting the conclusions of this article is included as a supplementary file. All data generated or analyzed during this study are also available on request from the respective author.

## Footnotes

1 The perception that the respondent has good relationships with co-workers and supervisor and that all information required to do one’s work tasks is provided and the organisation allows for personal development.
