# Supplementary material for: Importance of work engagement in primary healthcare
Source: BMC Health Serv Res. 2022 Aug 16;22:1044. doi: 10.1186/s12913-022-08402-7 (PMC9381147; doi:10.1186/s12913-022-08402-7)
Supplement: Supplementary file 1 — Additional file 1. [file 12913_2022_8402_MOESM1_ESM.pdf]

id	lastpage	q_	T1_1	T1_2	T1_3	T1_4	T1_5	T1_6	T1_7	T1_8	T1_9	T1_10	T1_11	T1_12	T1_13	T1_14	T1_15	T1_16	T1_17	T2_1	T2_2	T2_3	T2_4	T2_5	T2_6	T2_7	D6	D5	D5_other	D7	D7_other	D2	D2_other	D4	zavzetost	D5_recoded	D5_r_old	D5_drugo_R	D5_other_r
1	#NIČ!	5468156	#NIČ!	#NIČ!	#NIČ!	#NIČ!	#NIČ!	#NIČ!	#NIČ!	#NIČ!	#NIČ!	#NIČ!	#NIČ!	#NIČ!	#NIČ!	#NIČ!	#NIČ!	#NIČ!	#NIČ!	#NIČ!	#NIČ!	#NIČ!	#NIČ!	#NIČ!	#NIČ!	#NIČ!	#NIČ!	#NIČ!		#NIČ!		#NIČ!		#NIČ!	#NIČ!	#NIČ!	#NIČ!	34	#NIČ!
2	2	977907	6	7	7	6	5	4	5	4	4	7	5	4	5	6	5	2	5	3	4	4	4	3	4	4	9	#NIČ!		4		3		2	4,12	#NIČ!	#NIČ!	34	#NIČ!
3	2	8485067	5	6	6	5	4	6	4	3	6	6	6	6	4	6	6	3	6	4	3	3	3	2	4	3	2	2		2		4		2	4,18	2,00	5,00	34	#NIČ!
4	2	9491612	6	7	6	7	6	7	6	6	6	7	6	6	7	7	6	6	6	5	4	5	4	4	5	5	8	#NIČ!	Uprava	4		4		1	5,35	98,00	7,00	27	1,00
5	2	2002862	6	7	5	6	6	5	6	5	6	7	5	5	6	6	6	4	6	2	4	3	2	3	2	1	4	7		2		4		2	4,71	7,00	4,00	34	#NIČ!
6	2	1539472	4	4	6	5	4	4	4	3	5	3	4	6	4	5	5	2	6	3	3	3	3	2	3	3	4	1		2		5		2	3,35	1,00	2,00	34	#NIČ!
7	2	1688775	6	7	7	6	6	6	6	6	7	7	6	6	7	7	6	6	6	3	3	3	3	3	3	3	2	6		1		3		2	5,35	6,00	3,00	34	#NIČ!
8	2	3825457	7	7	7	6	6	7	6	6	6	6	6	5	7	7	6	5	7	4	4	3	4	4	4	4	6	1		2		2		1	5,29	1,00	2,00	34	#NIČ!
9	2	4060964	3	4	6	3	1	3	1	1	1	4	1	1	3	1	1	1	1	1	2	1	1	3	3	3	6	1		1		5		2	1,12	1,00	2,00	34	#NIČ!
10	#NIČ!	8828448	#NIČ!	#NIČ!	#NIČ!	#NIČ!	#NIČ!	#NIČ!	#NIČ!	#NIČ!	#NIČ!	#NIČ!	#NIČ!	#NIČ!	#NIČ!	#NIČ!	#NIČ!	#NIČ!	#NIČ!	#NIČ!	#NIČ!	#NIČ!	#NIČ!	#NIČ!	#NIČ!	#NIČ!	#NIČ!	#NIČ!		#NIČ!		#NIČ!		#NIČ!	#NIČ!	#NIČ!	#NIČ!	34	#NIČ!
11	#NIČ!	1959348	#NIČ!	#NIČ!	#NIČ!	#NIČ!	#NIČ!	#NIČ!	#NIČ!	#NIČ!	#NIČ!	#NIČ!	#NIČ!	#NIČ!	#NIČ!	#NIČ!	#NIČ!	#NIČ!	#NIČ!	#NIČ!	#NIČ!	#NIČ!	#NIČ!	#NIČ!	#NIČ!	#NIČ!	#NIČ!	#NIČ!		#NIČ!		#NIČ!		#NIČ!	#NIČ!	#NIČ!	#NIČ!	34	#NIČ!
12	2	3311397	6	7	7	7	7	5	7	6	7	7	6	6	7	6	6	6	6	3	5	5	5	3	5	5	2	14		2		4		2	5,41	14,00	1,00	34	#NIČ!
13	2	678943	5	6	6	6	4	4	3	4	5	4	3	3	3	3	4	3	5	4	4	4	4	3	3	3	8	#NIČ!	fin.-rač.	4		2		2	3,18	98,00	7,00	8	1,00
14	2	3460522	6	7	7	7	6	7	6	6	7	7	6	6	7	7	6	2	7	2	3	4	3	3	4	5	6	1		2		4		2	5,29	1,00	2,00	34	#NIČ!
15	2	5265781	3	7	7	5	5	4	5	5	4	6	4	5	4	5	4	4	6	3	3	3	3	3	2	3	3	5		2		3		2	3,88	5,00	3,00	34	#NIČ!
16	2	5947342	6	7	7	7	7	5	7	6	6	7	7	7	7	7	5	6	6	5	5	5	4	4	5	5	6	2		2		4		2	5,47	2,00	5,00	34	#NIČ!
17	2	3939364	6	5	6	7	7	7	7	6	6	7	7	6	7	7	4	2	5	1	5	5	4	4	5	5	2	14		2		4		2	5,00	14,00	1,00	34	#NIČ!
18	2	1854795	6	7	7	6	6	7	6	6	6	7	7	7	7	7	6	6	6	3	3	4	4	4	4	4	2	3		1		4		1	5,47	3,00	5,00	34	#NIČ!
19	2	7455883	7	7	7	7	7	7	7	7	7	7	7	7	7	7	7	7	7	5	5	5	5	5	5	5	3	7		2		1		2	6,00	7,00	4,00	34	#NIČ!
20	2	1715029	3	4	7	3	4	7	4	4	2	4	6	6	4	7	3	7	7	2	5	5	2	2	5	5	3	1		1		3		2	3,82	1,00	2,00	34	#NIČ!
21	2	6207497	6	7	6	7	6	6	6	7	6	7	6	6	6	7	6	5	7	4	4	4	4	3	4	4	8	#NIČ!		4		6		2	5,29	#NIČ!	#NIČ!	34	#NIČ!
22	2	5892400	6	6	6	6	6	4	6	5	6	6	6	6	6	6	4	4	5	3	4	3	3	3	3	3	5	12		3		4		2	4,53	12,00	1,00	34	#NIČ!
23	2	839507	6	7	7	7	5	6	6	5	6	7	7	6	7	7	6	5	6	3	4	4	3	3	3	2	2	25		2		4		2	5,24	98,00	1,00	34	#NIČ!
24	2	6520337	6	7	7	6	6	7	7	7	7	6	6	5	6	6	5	4	5	3	3	3	3	3	4	4	3	7		2		1		2	5,06	7,00	4,00	34	#NIČ!
25	0	83165	#NIČ!	#NIČ!	#NIČ!	#NIČ!	#NIČ!	#NIČ!	#NIČ!	#NIČ!	#NIČ!	#NIČ!	#NIČ!	#NIČ!	#NIČ!	#NIČ!	#NIČ!	#NIČ!	#NIČ!	#NIČ!	#NIČ!	#NIČ!	#NIČ!	#NIČ!	#NIČ!	#NIČ!	#NIČ!	#NIČ!		#NIČ!		#NIČ!		#NIČ!	#NIČ!	#NIČ!	#NIČ!	34	#NIČ!
26	2	854815	7	7	7	7	7	5	7	7	7	7	6	7	7	7	6	5	7	5	5	5	4	5	5	5	2	7		2		5		2	5,65	7,00	4,00	34	#NIČ!
27	2	4024581	7	7	7	5	4	4	2	4	4	5	4	4	4	4	4	4	5	2	4	4	3	3	3	3	6	4		1		5		1	3,59	4,00	6,00	34	#NIČ!
28	2	7558461	4	6	6	6	6	6	5	5	5	5	5	5	5	6	6	4	4	3	3	4	4	3	3	3	6	#NIČ!	adminis	#NIČ!	adm.	2		2	4,24	98,00	7,00	1	1,00
29	2	5718559	4	7	7	6	6	5	6	6	6	7	5	4	6	7	4	4	6	4	5	3	3	3	2	2	2	12		5		5		1	4,65	12,00	1,00	34	#NIČ!
30	2	5917414	6	5	7	6	4	7	4	4	7	4	4	4	3	4	6	3	6	4	3	3	3	3	3	3	6	17		2		5		2	3,94	17,00	1,00	34	#NIČ!
31	2	2431394	6	6	6	5	4	4	5	5	4	4	2	4	5	5	5	4	5	5	5	5	4	4	4	4	8	#NIČ!	uprava	5		#NIČ!	MBA	1	3,65	98,00	7,00	26	1,00
32	2	4404727	7	7	7	7	7	1	7	7	7	7	7	7	7	7	7	5	7	5	5	5	3	5	3	5	3	4		2		4		2	5,53	4,00	6,00	34	#NIČ!
33	2	4729454	6	7	7	6	6	7	6	6	6	6	6	6	7	7	6	6	7	4	5	4	4	4	4	4	3	7		2		3		2	5,35	7,00	4,00	34	#NIČ!
34	0	433090	#NIČ!	#NIČ!	#NIČ!	#NIČ!	#NIČ!	#NIČ!	#NIČ!	#NIČ!	#NIČ!	#NIČ!	#NIČ!	#NIČ!	#NIČ!	#NIČ!	#NIČ!	#NIČ!	#NIČ!	#NIČ!	#NIČ!	#NIČ!	#NIČ!	#NIČ!	#NIČ!	#NIČ!	#NIČ!	#NIČ!		#NIČ!		#NIČ!		#NIČ!	#NIČ!	#NIČ!	#NIČ!	34	#NIČ!
35	2	7977087	5	6	6	6	5	4	5	4	6	7	6	6	7	7	6	5	7	3	5	5	4	5	4	4	3	1		4		2		2	4,76	1,00	2,00	34	#NIČ!
36	2	8586164	6	7	7	6	6	6	6	6	6	7	6	6	7	7	6	5	7	5	3	4	4	3	2	2	4	#NIČ!	adminis	4		4		2	5,29	98,00	7,00	1	1,00
37	2	8999562	6	7	7	6	6	5	4	4	4	7	6	5	6	6	4	4	5	2	5	5	3	2	5	4	6	7		2		4		2	4,41	7,00	4,00	34	#NIČ!
38	2	9239317	6	7	7	7	6	6	6	6	6	7	7	6	7	6	6	6	6	4	4	4	4	4	5	4	3	7		2		4		2	5,35	7,00	4,00	34	#NIČ!
39	2	9197898	5	5	5	5	5	5	5	5	5	5	5	5	5	5	5	3	5	3	3	3	3	3	3	3	2	17		2		4		2	3,88	17,00	1,00	34	#NIČ!
40	2	8271539	6	7	7	7	7	4	7	6	7	7	6	6	7	7	6	6	6	3	5	5	5	4	3	4	3	5		1		4		2	5,41	5,00	3,00	34	#NIČ!
41	2	3764001	5	6	7	6	6	6	6	5	6	6	6	6	6	6	6	5	6	2	3	3	3	4	4	3	2	15		3		4		2	4,88	15,00	1,00	34	#NIČ!
42	2	4005388	4	6	7	6	6	7	7	6	7	7	7	6	7	7	4	5	7	2	4	2	3	2	3	3	2	#NIČ!	uprava	2		3		1	5,24	98,00	7,00	26	1,00
43	2	8734940	5	7	7	6	5	7	4	3	2	6	7	5	5	6	6	4	7	2	3	3	3	2	3	3	2	1		2		2		2	4,41	1,00	2,00	34	#NIČ!
44	#NIČ!	1658533	#NIČ!	#NIČ!	#NIČ!	#NIČ!	#NIČ!	#NIČ!	#NIČ!	#NIČ!	#NIČ!	#NIČ!	#NIČ!	#NIČ!	#NIČ!	#NIČ!	#NIČ!	#NIČ!	#NIČ!	#NIČ!	#NIČ!	#NIČ!	#NIČ!	#NIČ!	#NIČ!	#NIČ!	#NIČ!	#NIČ!		#NIČ!		#NIČ!		#NIČ!	#NIČ!	#NIČ!	#NIČ!	34	#NIČ!
45	2	2087847	4	6	7	6	6	6	5	4	5	6	6	6	6	6	4	4	5	2	3	3	2	1	2	2	7	1		4		4		2	4,41	1,00	2,00	34	#NIČ!
46	2	5463634	7	7	7	7	7	7	7	7	7	7	7	7	7	7	7	1	7	5	5	3	3	5	5	5	3	7		2		4		2	5,65	7,00	4,00	34	#NIČ!
47	2	1054632	4	4	5	3	4	5	4	4	6	5	6	4	4	6	5	3	7	3	4	4	3	3	3	3	4	#NIČ!		4		2		2	3,65	#NIČ!	#NIČ!	34	#NIČ!
48	2	8882256	6	7	7	6	7	7	6	6	7	7	6	7	7	7	2	4	7	4	5	5	5	5	5	5	3	#NIČ!	informa	4		4		2	5,24	98,00	7,00	11	1,00
49	2	1247385	6	7	7	6	6	6	6	6	7	7	6	5	6	6	6	4	5	4	5	5	5	5	5	5	2	15		3		4		2	5,00	15,00	1,00	34	#NIČ!
50	2	9590158	7	7	7	5	5	4	4	3	6	4	4	5	4	5	5	4	6	5	5	5	3	3	4	4	8	#NIČ!		4		5		2	4,00	#NIČ!	#NIČ!	34	#NIČ!
51	2	4208634	3	3	3	3	3	2	2	1	2	4	3	4	4	3	3	7	4	2	5	3	2	2	2	1	4	1		2		2		2	2,18	1,00	2,00	34	#NIČ!

52	2	2272699	4	6	6	6	7	6	6	5	6	7	6	5	7	6	5	3	6	2	4	4	4	3	4	4	6	2		2		4	2	4,71	2,00	5,00	34	#NIČ!
53	2	8737591	6	7	7	6	4	6	5	4	4	5	4	4	5	6	4	5	6	3	4	4	4	3	5	4	3	#NIČ!	Adminis	#NIČ!	poslov	3	2	4,18	98,00	7,00	2	1,00
54	2	6869861	4	5	5	5	5	6	3	4	2	3	2	5	4	3	1	2	5	2	3	2	1	1	2	2	8	#NIČ!		4		3	2	2,76	#NIČ!	#NIČ!	34	#NIČ!
55	#NIČ!	8136531	#NIČ!	#NIČ!	#NIČ!	#NIČ!	#NIČ!	#NIČ!	#NIČ!	#NIČ!	#NIČ!	#NIČ!	#NIČ!	#NIČ!	#NIČ!	#NIČ!	#NIČ!	#NIČ!	#NIČ!	#NIČ!	#NIČ!	#NIČ!	#NIČ!	#NIČ!	#NIČ!	#NIČ!	#NIČ!	#NIČ!		#NIČ!		#NIČ!	#NIČ!	#NIČ!	#NIČ!	#NIČ!	34	#NIČ!
56	2	73071	6	7	7	6	6	7	6	6	7	7	6	7	7	7	6	7	7	4	5	5	4	4	4	4	4	#NIČ!		2		3	1	5,59	#NIČ!	#NIČ!	34	#NIČ!
57	2	5955765	5	6	6	6	5	6	5	6	6	6	5	5	5	6	6	5	6	4	4	4	3	3	3	3	8	#NIČ!		#NIČ!		4	2	4,59	#NIČ!	#NIČ!	34	#NIČ!
58	2	9559611	5	5	4	4	4	4	5	4	4	5	5	4	5	5	4	4	6	4	4	4	4	4	4	3	8	#NIČ!	UPRAVA	4		4	2	3,53	98,00	7,00	28	1,00
59	2	9930760	6	5	6	6	6	6	6	5	6	6	6	5	6	6	6	5	6	3	3	3	2	2	2	2	2	12		3		6	2	4,76	12,00	1,00	34	#NIČ!
60	2	974966	4	6	6	5	5	6	6	5	6	6	6	6	6	6	5	6	6	3	3	3	3	3	3	3	8	#NIČ!	uprava	4		3	2	4,65	98,00	7,00	26	1,00
61	2	5082553	7	7	7	7	7	6	7	7	7	7	7	7	7	7	7	6	6	3	5	5	3	3	4	3	2	21		2		2	2	5,82	21,00	1,00	34	#NIČ!
62	2	2487868	6	7	7	6	6	7	7	6	7	7	6	6	6	7	6	5	6	4	4	4	4	4	5	4	3	7		2		4	1	5,35	7,00	4,00	34	#NIČ!
63	2	7191679	6	5	7	6	5	6	4	4	4	4	5	6	4	6	4	2	7	5	5	1	3	3	3	1	6	17		2		4	2	4,00	17,00	1,00	34	#NIČ!
64	2	8494793	4	7	4	5	5	5	5	4	4	7	4	4	4	4	3	2	4	3	3	3	3	3	3	3	6	6		2		2	2	3,41	6,00	3,00	34	#NIČ!
65	2	898927	6	7	7	7	7	7	7	7	7	7	7	7	7	7	7	6	7	5	5	5	5	5	5	5	8	#NIČ!	Uprava	4		4	2	5,88	98,00	7,00	27	1,00
66	2	9010256	6	7	6	6	5	6	6	6	5	6	6	6	7	6	5	4	6	4	5	5	5	5	5	4	5	7		2		3	2	4,82	7,00	4,00	34	#NIČ!
67	2	2354501	7	7	7	7	6	7	6	6	6	7	7	7	7	7	7	7	7	4	4	5	3	4	5	4	1	26		1		5	1	5,76	26,00	1,00	34	#NIČ!
68	#NIČ!	4741737	#NIČ!	#NIČ!	#NIČ!	#NIČ!	#NIČ!	#NIČ!	#NIČ!	#NIČ!	#NIČ!	#NIČ!	#NIČ!	#NIČ!	#NIČ!	#NIČ!	#NIČ!	#NIČ!	#NIČ!	#NIČ!	#NIČ!	#NIČ!	#NIČ!	#NIČ!	#NIČ!	#NIČ!	#NIČ!	#NIČ!		#NIČ!		#NIČ!	#NIČ!	#NIČ!	#NIČ!	#NIČ!	34	#NIČ!
69	2	6645184	6	6	7	6	6	6	6	6	6	6	6	6	5	6	6	5	6	4	5	5	4	3	3	3	2	9		3		4	2	4,94	9,00	1,00	34	#NIČ!
70	2	9000707	6	6	6	6	6	7	7	6	7	7	6	7	7	6	5	4	7	4	4	4	4	4	4	4	9	#NIČ!	IRROZ	1		7	2	5,24	98,00	7,00	13	1,00
71	2	5067984	7	7	7	7	4	7	4	2	6	7	7	7	7	7	3	7	5	2	3	3	2	3	2	2	8	#NIČ!	uprava	3		4	2	4,94	98,00	7,00	26	1,00
72	2	8337799	6	6	7	7	6	6	6	7	7	7	7	7	7	7	7	6	6	4	5	4	3	3	4	4	1	7		2		3	2	5,59	7,00	4,00	34	#NIČ!
73	2	6485042	6	6	6	5	6	4	5	6	4	6	5	5	6	6	5	4	6	3	4	4	4	3	4	3	8	#NIČ!		4		5	2	4,35	#NIČ!	#NIČ!	34	#NIČ!
74	2	7411815	7	7	7	7	7	7	7	7	7	7	7	7	7	7	7	7	7	5	5	5	5	5	5	5	7	#NIČ!		1		5	2	6,00	#NIČ!	#NIČ!	34	#NIČ!
75	2	7603949	2	5	7	4	2	3	2	2	3	4	3	3	2	2	2	1	5	2	5	3	3	3	3	2	1	5		3		2	2	2,06	5,00	3,00	34	#NIČ!
76	2	5784301	7	7	7	7	7	7	7	7	7	7	7	7	7	7	7	7	7	5	5	5	5	5	5	5	8	#NIČ!	Uprava	4		4	2	6,00	98,00	7,00	27	1,00
77	2	6109657	7	6	6	6	6	6	6	6	6	6	6	6	6	6	6	5	6	3	4	4	4	3	4	4	6	#NIČ!		2		4	1	5,00	#NIČ!	#NIČ!	34	#NIČ!
78	2	3195382	5	5	7	5	5	3	4	3	2	5	3	1	6	5	4	3	7	2	4	3	3	3	4	4	7	#NIČ!	nmp	1		6	1	3,29	98,00	7,00	14	1,00
79	2	7647938	6	6	5	6	6	6	6	6	6	6	6	6	6	6	4	6	6	3	4	4	4	4	4	4	5	1		1		5	1	4,82	1,00	2,00	34	#NIČ!
80	2	8653546	6	7	6	7	7	6	6	6	5	6	6	4	5	6	5	5	5	3	4	3	3	3	2	3	3	18		3		2	1	4,76	18,00	3,00	34	#NIČ!
81	2	323915	3	7	6	6	3	6	5	5	2	7	4	1	7	6	3	1	6	2	4	2	2	3	1	1	1	8		3		4	2	3,59	8,00	1,00	34	#NIČ!
82	2	5658938	6	6	7	5	5	7	6	5	5	6	7	6	6	6	5	6	7	5	5	5	4	5	5	5	8	#NIČ!	Uprava	4		4	2	4,94	98,00	7,00	27	1,00
83	2	7322944	7	7	6	5	5	6	5	6	4	5	5	5	4	5	6	5	5	4	5	4	4	4	4	4	8	#NIČ!		#NIČ!		2	2	4,35	#NIČ!	#NIČ!	34	#NIČ!
84	#NIČ!	9637908	#NIČ!	#NIČ!	#NIČ!	#NIČ!	#NIČ!	#NIČ!	#NIČ!	#NIČ!	#NIČ!	#NIČ!	#NIČ!	#NIČ!	#NIČ!	#NIČ!	#NIČ!	#NIČ!	#NIČ!	#NIČ!	#NIČ!	#NIČ!	#NIČ!	#NIČ!	#NIČ!	#NIČ!	#NIČ!	#NIČ!		#NIČ!		#NIČ!	#NIČ!	#NIČ!	#NIČ!	#NIČ!	34	#NIČ!
85	2	6220709	6	6	7	6	6	5	7	7	7	7	7	6	7	6	4	5	6	4	5	5	4	4	5	5	9	#NIČ!		#NIČ!		7	2	5,18	#NIČ!	#NIČ!	34	#NIČ!
86	2	2189820	7	7	6	7	6	6	6	6	6	6	6	6	6	6	6	6	7	4	5	5	4	4	5	4	2	#NIČ!	fiziatričr	2		2	2	5,24	98,00	7,00	9	1,00
87	2	2286972	6	6	7	6	6	6	6	5	5	7	6	6	6	6	5	4	7	4	5	5	4	4	5	4	6	1		1		5	2	4,88	1,00	2,00	34	#NIČ!
88	2	4865400	4	7	7	7	6	7	6	6	6	7	7	6	6	7	4	1	7	3	3	3	3	3	3	3	8	#NIČ!	uprava	4		4	1	4,94	98,00	7,00	26	1,00
89	2	7466087	6	7	7	6	6	6	6	6	6	6	7	7	6	7	6	6	7	2	4	3	2	2	3	2	6	3		1		6	2	5,35	3,00	5,00	34	#NIČ!
90	2	2734233	6	6	6	6	5	6	5	5	5	5	6	6	5	6	6	5	6	3	5	5	4	4	4	3	3	#NIČ!		4		3	2	4,59	#NIČ!	#NIČ!	34	#NIČ!
91	1	1272906	6	6	7	6	5	6	6	6	6	6	6	6	6	6	6	5	6	1	4	4	2	3	2	2	#NIČ!	#NIČ!		#NIČ!		#NIČ!	#NIČ!	4,94	#NIČ!	#NIČ!	34	#NIČ!
92	2	8161829	5	7	7	6	5	6	5	5	6	7	4	5	5	4	6	3	6	2	4	3	3	3	4	3	3	7		2		2	2	4,41	7,00	4,00	34	#NIČ!
93	2	6990430	7	7	7	7	6	6	6	6	6	6	6	6	7	7	7	6	7	2	4	3	3	3	4	4	2	25		1		6	1	5,47	98,00	1,00	34	#NIČ!
94	2	466664	7	7	7	7	7	6	6	6	7	7	7	6	6	7	6	4	7	4	4	4	4	4	4	4	4	14		2		5	2	5,47	14,00	1,00	34	#NIČ!
95	2	1362028	7	7	7	6	6	6	7	7	7	6	6	6	7	7	7	6	7	4	5	5	5	4	5	5	9	#NIČ!	adminis	4		3	2	5,59	98,00	7,00	1	1,00
96	2	5410150	6	5	7	6	6	6	5	5	6	6	5	6	5	6	6	2	6	2	5	5	3	4	3	2	2	#NIČ!		4		2	2	4,53	#NIČ!	#NIČ!	34	#NIČ!
97	2	2964666	4	5	5	4	4	4	4	4	4	4	4	4	4	4	4	4	4	2	3	3	3	3	3	3	2	17		2		2	2	3,12	17,00	1,00	34	#NIČ!
98	2	8592879	6	7	7	7	6	6	6	6	7	7	7	7	7	7	6	3	7	2	5	5	4	5	5	5	3	1		2		3	1	5,41	1,00	2,00	34	#NIČ!
99	2	4070399	1	1	1	1	1	1	1	1	1	1	1	1	1	1	1	1	1	1	1	1	1	1	1	1	5	#NIČ!		#NIČ!		#NIČ!	2	0,00	#NIČ!	#NIČ!	34	#NIČ!
100	2	4573357	6	7	7	7	7	6	7	6	6	7	4	6	7	7	7	3	7	5	5	5	5	5	5	5	1	#NIČ!	Vodstvo	2		4	1	5,29	98,00	7,00	31	1,00
101	2	655590	6	7	5	5	5	4	4	4	4	5	5	5	4	5	5	3	5	2	4	3	3	3	3	3	2	6		2		2	2	3,76	6,00	3,00	34	#NIČ!
102	#NIČ!	9557879	#NIČ!	#NIČ!	#NIČ!	#NIČ!	#NIČ!	#NIČ!	#NIČ!	#NIČ!	#NIČ!	#NIČ!	#NIČ!	#NIČ!	#NIČ!	#NIČ!	#NIČ!	#NIČ!	#NIČ!	#NIČ!	#NIČ!	#NIČ!	#NIČ!	#NIČ!	#NIČ!	#NIČ!	#NIČ!	#NIČ!		#NIČ!		#NIČ!	#NIČ!	#NIČ!	#NIČ!	#NIČ!	34	#NIČ!
103	2	5822625	6	6	6	4	5	5	5	6	6	5	5	6	5	6	5	4	5	3	4	4	4	4	4	3	6	1		2		2	2	4,29	1,00	2,00	34	#NIČ!
104	2	439486	6	7	7	7	7	6	6	6	7	7	6	6	7	7	6	6	6	3	4	2	2	2	4	2	1	1		1		5	2	5,47	1,00	2,00	34	#NIČ!
105	2	4729558	6	7	7	7	6	3	6	6	6	6	5	5	6	7	6	4	7	4	5	5	4	4	4	4	3	#NIČ!		5		5	1	4,88	#NIČ!	#NIČ!	34	#NIČ!

106	2	2329333	6	7	7	6	6	6	6	6	6	7	6	6	6	6	6	4	6	2	4	4	4	5	5	5	2	7		2	4	1	5,06	7,00	4,00	34	#NIČ!
107	2	7457988	5	7	6	6	6	6	6	4	2	6	6	6	5	5	2	3	5	3	3	3	3	3	2	2	6	18		3	2	2	4,06	18,00	3,00	34	#NIČ!
108	#NIČ!	301945	#NIČ!	#NIČ!	#NIČ!	#NIČ!	#NIČ!	#NIČ!	#NIČ!	#NIČ!	#NIČ!	#NIČ!	#NIČ!	#NIČ!	#NIČ!	#NIČ!	#NIČ!	#NIČ!	#NIČ!	#NIČ!	#NIČ!	#NIČ!	#NIČ!	#NIČ!	#NIČ!	#NIČ!	#NIČ!	#NIČ!		#NIČ!	#NIČ!	#NIČ!	#NIČ!	#NIČ!	#NIČ!	34	#NIČ!
109	#NIČ!	9135759	#NIČ!	#NIČ!	#NIČ!	#NIČ!	#NIČ!	#NIČ!	#NIČ!	#NIČ!	#NIČ!	#NIČ!	#NIČ!	#NIČ!	#NIČ!	#NIČ!	#NIČ!	#NIČ!	#NIČ!	#NIČ!	#NIČ!	#NIČ!	#NIČ!	#NIČ!	#NIČ!	#NIČ!	#NIČ!	#NIČ!		#NIČ!	#NIČ!	#NIČ!	#NIČ!	#NIČ!	#NIČ!	34	#NIČ!
110	2	4772960	4	4	7	6	5	7	5	3	6	6	5	5	4	6	5	1	6	4	4	2	3	4	2	2	5	1		2	2	2	4,00	1,00	2,00	34	#NIČ!
111	2	6457522	5	7	6	6	7	5	5	4	2	6	4	2	5	7	4	2	6	4	4	4	2	4	2	2	6	5		1	4	2	3,88	5,00	3,00	34	#NIČ!
112	2	7968733	6	7	7	6	7	7	7	7	7	7	7	7	7	7	6	6	7	3	4	4	3	4	4	3	4	6		1	4	2	5,76	6,00	3,00	34	#NIČ!
113	2	471096	4	6	7	4	4	6	4	4	2	3	4	1	7	5	4	1	7	3	3	3	2	2	3	3	3	1		2	2	2	3,29	1,00	2,00	34	#NIČ!
114	2	8449284	4	5	5	5	5	5	5	5	5	5	5	6	5	6	5	5	6	4	5	4	3	4	4	4	8	#NIČ!		4	2	2	4,12	#NIČ!	#NIČ!	34	#NIČ!
115	2	833132	6	7	7	6	6	7	6	7	7	7	7	7	6	7	7	6	6	5	5	5	5	5	2	3	3	6		2	3	2	5,59	6,00	3,00	34	#NIČ!
116	2	8817810	6	6	7	6	6	6	6	5	7	7	6	6	6	6	5	6	7	3	4	4	3	3	3	3	3	1		2	4	2	5,12	1,00	2,00	34	#NIČ!
117	2	1589653	6	7	7	6	6	6	6	5	6	7	6	6	6	7	6	5	6	2	5	2	3	3	2	2	5	8		3	4	2	5,12	8,00	1,00	34	#NIČ!
118	2	1494837	6	7	7	7	6	7	6	4	4	6	4	7	7	7	5	1	5	2	3	3	3	3	3	3	4	7		2	2	2	4,65	7,00	4,00	34	#NIČ!
120	2	2705223	6	6	7	6	5	7	6	5	6	6	7	6	7	7	5	5	4	3	4	4	4	4	4	3	6	1		2	2	2	4,94	1,00	2,00	34	#NIČ!
121	2	9041607	6	7	7	6	6	6	6	6	6	7	7	6	6	6	6	4	7	4	5	5	4	5	4	4	2	#NIČ!	radiološ	1	5	2	5,18	98,00	7,00	16	1,00
122	2	7092365	5	7	7	6	5	5	4	4	4	7	4	4	4	7	5	4	6	3	4	3	3	2	3	2	3	17		2	4	2	4,18	17,00	1,00	34	#NIČ!
123	2	8337005	6	6	5	5	5	6	6	6	6	5	5	6	6	5	5	2	5	3	3	3	3	3	3	2	6	4		2	4	2	4,29	4,00	6,00	34	#NIČ!
124	2	407929	6	6	7	5	5	6	5	3	3	4	3	6	4	3	2	1	6	2	3	3	3	3	3	2	2	3		1	5	2	3,41	3,00	5,00	34	#NIČ!
125	2	7028631	6	6	6	5	6	6	7	4	7	6	4	5	6	6	6	3	7	3	4	5	5	4	5	5	8	#NIČ!		#NIČ!	5	2	4,65	#NIČ!	#NIČ!	34	#NIČ!
126	2	3919369	6	7	6	6	6	4	6	6	4	7	5	4	7	5	6	4	5	2	5	4	3	3	2	2	3	2		1	5	2	4,53	2,00	5,00	34	#NIČ!
127	2	8510950	6	7	7	6	6	6	6	6	7	6	6	6	6	6	6	6	7	4	3	4	4	4	3	4	6	17		2	2	2	5,24	17,00	1,00	34	#NIČ!
128	2	796647	7	7	7	7	7	7	7	7	7	7	7	7	7	7	7	7	7	5	5	5	5	5	5	5	3	5		1	4	2	6,00	5,00	3,00	34	#NIČ!
129	2	8450382	7	7	7	7	6	6	6	6	6	7	3	6	7	7	6	5	6	3	5	4	4	4	4	3	3	17		2	2	2	5,18	17,00	1,00	34	#NIČ!
130	#NIČ!	9861969	#NIČ!	#NIČ!	#NIČ!	#NIČ!	#NIČ!	#NIČ!	#NIČ!	#NIČ!	#NIČ!	#NIČ!	#NIČ!	#NIČ!	#NIČ!	#NIČ!	#NIČ!	#NIČ!	#NIČ!	#NIČ!	#NIČ!	#NIČ!	#NIČ!	#NIČ!	#NIČ!	#NIČ!	#NIČ!	#NIČ!		#NIČ!	#NIČ!	#NIČ!	#NIČ!	#NIČ!	#NIČ!	34	#NIČ!
131	2	3958702	5	6	7	6	5	5	6	6	6	6	7	6	6	6	6	5	6	5	5	5	4	5	5	5	3	1		1	5	1	4,88	1,00	2,00	34	#NIČ!
132	2	207601	6	7	7	7	4	6	4	4	4	6	6	6	4	7	4	1	7	4	4	5	5	5	5	5	2	16		1	5	2	4,29	16,00	1,00	34	#NIČ!
133	2	9161898	6	7	6	6	6	4	6	6	6	7	6	5	6	6	6	4	7	4	5	5	5	4	5	5	8	#NIČ!	Uprava	5	6	1	4,88	98,00	7,00	27	1,00
134	2	5186689	6	7	7	7	7	7	7	6	7	7	7	7	7	7	6	6	7	3	4	4	4	4	2	3	4	6		2	4	2	5,76	6,00	3,00	34	#NIČ!
135	2	8447751	4	6	5	5	4	6	4	3	3	6	5	3	6	6	5	4	6	2	4	4	4	3	4	3	6	9		3	4	2	3,76	9,00	1,00	34	#NIČ!
136	2	6678687	6	6	6	6	6	7	6	6	6	7	6	6	7	7	6	5	6	3	5	5	3	4	4	3	3	14		2	4	2	5,18	14,00	1,00	34	#NIČ!
137	2	8050183	7	7	7	6	6	7	7	6	6	6	6	7	6	6	6	2	6	2	5	5	5	4	4	4	6	#NIČ!	dso AMI	1	5	2	5,12	98,00	7,00	7	1,00
138	2	214854	6	7	7	6	6	6	5	6	4	6	5	4	4	6	6	3	7	2	5	5	3	3	3	3	1	2		1	5	2	4,53	2,00	5,00	34	#NIČ!
139	2	6923724	4	4	7	7	7	4	3	4	7	7	7	6	6	7	7	4	5	3	3	3	3	3	3	2	2	17		2	2	2	4,65	17,00	1,00	34	#NIČ!
140	2	3974055	4	5	3	4	4	5	4	4	4	5	4	4	3	5	6	3	5	2	5	3	3	3	2	2	6	1		2	4	2	3,24	1,00	2,00	34	#NIČ!
141	2	9099106	3	7	7	4	6	2	4	2	3	7	2	4	5	4	4	4	5	2	3	3	3	3	3	2	6	2		2	2	2	3,29	2,00	5,00	34	#NIČ!
142	2	3573364	4	6	6	4	5	4	4	3	3	4	5	7	5	6	2	3	6	2	3	3	2	2	2	2	6	12		3	4	2	3,53	12,00	1,00	34	#NIČ!
143	#NIČ!	569501	#NIČ!	#NIČ!	#NIČ!	#NIČ!	#NIČ!	#NIČ!	#NIČ!	#NIČ!	#NIČ!	#NIČ!	#NIČ!	#NIČ!	#NIČ!	#NIČ!	#NIČ!	#NIČ!	#NIČ!	#NIČ!	#NIČ!	#NIČ!	#NIČ!	#NIČ!	#NIČ!	#NIČ!	#NIČ!	#NIČ!		#NIČ!	#NIČ!	#NIČ!	#NIČ!	#NIČ!	#NIČ!	34	#NIČ!
144	2	2127412	6	6	6	5	5	5	5	5	6	4	4	4	5	6	5	4	6	4	3	4	3	4	5	5	4	4		2	2	2	4,12	4,00	6,00	34	#NIČ!
145	2	8928560	6	7	6	7	7	6	7	6	7	7	6	6	6	7	7	4	6	4	5	5	4	4	4	4	2	5		1	4	2	5,35	5,00	3,00	34	#NIČ!
146	2	8260565	7	7	7	7	6	7	7	6	7	7	6	6	7	7	6	3	7	5	4	5	5	4	5	5	1	#NIČ!	tajništvc	4	3	2	5,47	98,00	7,00	24	1,00
147	2	4517144	6	7	7	7	6	7	6	6	6	6	6	6	6	7	6	5	6	4	4	3	4	4	5	5	2	1		1	6	1	5,24	1,00	2,00	34	#NIČ!
148	2	7804028	4	7	4	4	4	4	4	5	5	6	6	3	4	7	4	5	6	3	3	3	3	3	3	3	4	1		2	2	2	3,82	1,00	2,00	34	#NIČ!
149	#NIČ!	5468705	#NIČ!	#NIČ!	#NIČ!	#NIČ!	#NIČ!	#NIČ!	#NIČ!	#NIČ!	#NIČ!	#NIČ!	#NIČ!	#NIČ!	#NIČ!	#NIČ!	#NIČ!	#NIČ!	#NIČ!	#NIČ!	#NIČ!	#NIČ!	#NIČ!	#NIČ!	#NIČ!	#NIČ!	#NIČ!	#NIČ!		#NIČ!	#NIČ!	#NIČ!	#NIČ!	#NIČ!	#NIČ!	34	#NIČ!
150	2	3931443	7	7	7	7	7	7	6	5	6	6	6	7	6	7	6	6	7	2	4	4	5	5	5	5	7	#NIČ!	nmp	2	4	2	5,47	98,00	7,00	14	1,00
151	2	3251101	4	5	5	5	5	4	5	4	5	5	4	4	4	6	3	3	6	2	2	2	3	2	3	2	6	9		2	2	2	3,53	9,00	1,00	34	#NIČ!
152	2	4461173	6	7	7	6	6	6	6	6	5	6	6	5	6	6	4	4	6	3	5	5	2	3	2	2	6	5		1	5	2	4,76	5,00	3,00	34	#NIČ!
153	2	2552566	6	7	7	5	5	6	5	5	4	5	4	3	5	5	5	2	5	4	5	5	4	4	3	3	2	8		3	4	2	3,94	8,00	1,00	34	#NIČ!
154	2	9379310	7	7	6	6	7	7	7	6	6	7	6	7	7	7	6	4	7	4	5	5	5	5	3	4	2	6		2	2	2	5,47	6,00	3,00	34	#NIČ!
155	2	9238854	6	6	6	6	5	6	5	5	6	7	7	7	7	6	7	5	7	5	5	5	4	3	5	5	8	#NIČ!	Uprava	5	4	1	5,12	98,00	7,00	27	1,00
156	2	8056341	6	7	7	7	7	6	7	6	6	7	6	7	7	7	7	7	7	2	3	3	3	4	3	3	1	4		1	5	1	5,71	4,00	6,00	34	#NIČ!
157	2	2565141	6	6	7	7	6	6	6	5	6	6	5	6	6	6	5	4	6	4	4	5	3	5	5	5	3	26		2	4	1	4,82	26,00	1,00	34	#NIČ!
158	2	8656682	4	6	7	5	6	4	3	2	4	7	5	7	6	7	7	5	7	3	5	3	3	3	2	2	6	5		2	2	2	4,41	5,00	3,00	34	#NIČ!
159	2	5587989	6	7	7	7	6	6	6	6	6	6	6	6	6	6	5	5	5	3	3	3	2	3	3	3	2	22		1	5	1	5,00	98,00	1,00	34	#NIČ!
160	2	1969898	6	7	7	7	5	7	5	5	6	6	7	7	6	6	6	6	7	5	5	5	4	4	5	4	8	#NIČ!		4	4	1	5,24	#NIČ!	#NIČ!	34	#NIČ!

161	2	3085526	4	5	7	5	6	5	5	4	4	5	5	5	6	5	4	3	5	3	4	4	4	4	4	4	2	8		3	4	2	3,88	8,00	1,00	34	#NIČ!
162	2	9517933	7	7	7	7	7	7	7	7	7	7	7	7	7	7	7	7	7	5	5	5	5	5	5	5	6	1		2	4	2	6,00	1,00	2,00	34	#NIČ!
163	#NIČ!	8333088	#NIČ!	#NIČ!	#NIČ!	#NIČ!	#NIČ!	#NIČ!	#NIČ!	#NIČ!	#NIČ!	#NIČ!	#NIČ!	#NIČ!	#NIČ!	#NIČ!	#NIČ!	#NIČ!	#NIČ!	#NIČ!	#NIČ!	#NIČ!	#NIČ!	#NIČ!	#NIČ!	#NIČ!	#NIČ!	#NIČ!		#NIČ!	#NIČ!	#NIČ!	#NIČ!	#NIČ!	#NIČ!	34	#NIČ!
164	2	3111642	6	6	6	5	6	5	5	6	6	6	5	6	6	5	5	4	5	4	4	4	3	4	3	3	1	1		2	2	2	4,47	1,00	2,00	34	#NIČ!
165	2	558947	5	4	7	5	5	5	4	4	4	5	4	4	7	6	6	3	7	3	4	5	3	1	4	3	8	#NIČ!		4	3	2	4,00	#NIČ!	#NIČ!	34	#NIČ!
166	2	3459810	6	5	5	5	5	4	4	3	4	5	4	5	4	5	4	2	5	3	3	3	3	3	3	3	4	12		3	2	2	3,41	12,00	1,00	34	#NIČ!
167	1	5622210	6	7	7	7	7	7	7	7	6	7	6	6	7	7	6	5	6	3	4	5	3	3	3	2	#NIČ!	#NIČ!		#NIČ!	#NIČ!	#NIČ!	5,53	#NIČ!	#NIČ!	34	#NIČ!
168	2	7731618	6	5	7	5	4	6	5	5	5	5	5	4	6	6	5	4	7	5	5	5	5	4	5	4	8	#NIČ!	Adminis	4	4	2	4,29	98,00	7,00	3	1,00
169	2	1791460	7	7	7	7	7	6	6	7	7	7	7	7	7	7	7	6	6	4	5	5	3	3	4	3	5	14		2	4	1	5,76	14,00	1,00	34	#NIČ!
170	2	5762447	5	6	6	6	6	6	6	6	5	6	5	5	6	6	5	5	6	3	4	5	4	3	3	3	2	17		1	4	2	4,65	17,00	1,00	34	#NIČ!
171	#NIČ!	3437857	#NIČ!	#NIČ!	#NIČ!	#NIČ!	#NIČ!	#NIČ!	#NIČ!	#NIČ!	#NIČ!	#NIČ!	#NIČ!	#NIČ!	#NIČ!	#NIČ!	#NIČ!	#NIČ!	#NIČ!	#NIČ!	#NIČ!	#NIČ!	#NIČ!	#NIČ!	#NIČ!	#NIČ!	#NIČ!	#NIČ!		#NIČ!	#NIČ!	#NIČ!	#NIČ!	#NIČ!	#NIČ!	34	#NIČ!
172	2	9901943	6	6	7	7	7	5	6	6	6	7	7	7	6	7	4	7	6	4	3	4	4	4	4	5	1	2		2	4	2	5,29	2,00	5,00	34	#NIČ!
173	2	9196143	7	7	7	7	7	7	7	7	7	7	7	7	7	7	7	3	5	4	5	5	4	4	5	5	2	17		2	4	2	5,65	17,00	1,00	34	#NIČ!
174	2	6274886	6	7	6	6	6	6	6	6	6	6	5	4	6	6	5	4	5	3	3	3	3	3	3	3	6	15		3	4	2	4,65	15,00	1,00	34	#NIČ!
175	2	3786002	6	6	6	6	4	5	4	3	3	4	6	6	6	5	6	3	4	4	4	3	3	3	3	3	2	17		1	6	1	3,88	17,00	1,00	34	#NIČ!
176	2	105355	6	6	7	6	6	7	6	6	6	7	5	6	6	5	6	4	5	3	5	5	5	4	3	3	4	11		3	4	2	4,88	11,00	1,00	34	#NIČ!
177	2	9168766	6	6	7	6	6	6	6	6	6	6	5	5	6	6	6	5	5	2	3	3	3	2	2	3	1	5		1	4	2	4,82	5,00	3,00	34	#NIČ!
178	2	5527765	6	7	7	7	7	7	7	7	5	7	5	5	7	7	7	7	7	3	5	5	3	5	5	3	3	14		5	6	1	5,59	14,00	1,00	34	#NIČ!
179	2	132526	6	6	6	6	7	7	7	6	7	7	7	7	7	7	7	5	7	3	5	5	4	4	4	4	3	5		1	5	2	5,59	5,00	3,00	34	#NIČ!
180	2	4079338	4	5	5	5	5	5	5	5	5	5	5	5	5	6	6	5	5	4	4	4	4	4	4	4	3	1		2	2	2	4,06	1,00	2,00	34	#NIČ!
181	2	9999112	4	3	4	3	3	4	2	2	2	3	2	3	2	3	4	1	7	2	5	5	3	3	3	3	8	#NIČ!	adminis	4	2	2	2,06	98,00	7,00	1	1,00
182	2	7757545	6	7	7	7	6	7	6	6	6	7	7	7	7	7	7	7	7	5	5	3	2	3	4	3	6	1		1	5	2	5,71	1,00	2,00	34	#NIČ!
183	#NIČ!	8790390	#NIČ!	#NIČ!	#NIČ!	#NIČ!	#NIČ!	#NIČ!	#NIČ!	#NIČ!	#NIČ!	#NIČ!	#NIČ!	#NIČ!	#NIČ!	#NIČ!	#NIČ!	#NIČ!	#NIČ!	#NIČ!	#NIČ!	#NIČ!	#NIČ!	#NIČ!	#NIČ!	#NIČ!	#NIČ!	#NIČ!		#NIČ!	#NIČ!	#NIČ!	#NIČ!	#NIČ!	#NIČ!	34	#NIČ!
184	2	679315	4	7	7	7	5	5	4	4	4	7	4	4	7	7	7	4	7	3	3	3	3	3	4	3	2	16		2	4	1	4,53	16,00	1,00	34	#NIČ!
185	2	7025406	7	4	7	7	7	7	7	7	7	7	7	7	7	7	5	3	7	2	5	5	3	3	2	2	6	9		4	2	2	5,47	9,00	1,00	34	#NIČ!
186	2	3089085	6	7	7	6	6	3	2	6	5	7	3	4	4	7	4	2	7	4	4	4	4	3	5	3	1	#NIČ!	adminis	4	2	2	4,06	98,00	7,00	1	1,00
187	2	4369205	4	5	5	4	4	4	4	5	4	5	4	4	4	5	3	3	4	2	3	3	1	3	2	2	2	9		3	3	2	3,18	9,00	1,00	34	#NIČ!
188	2	2578773	4	7	7	4	4	5	3	3	7	7	4	4	4	4	3	4	7	2	4	4	3	3	3	3	8	#NIČ!	Uprava	3	4	2	3,76	98,00	7,00	27	1,00
189	2	9786248	6	7	7	7	7	6	6	7	6	7	7	7	7	7	6	6	6	2	4	2	1	5	4	3	1	14		2	4	2	5,59	14,00	1,00	34	#NIČ!
190	#NIČ!	1194922	#NIČ!	#NIČ!	#NIČ!	#NIČ!	#NIČ!	#NIČ!	#NIČ!	#NIČ!	#NIČ!	#NIČ!	#NIČ!	#NIČ!	#NIČ!	#NIČ!	#NIČ!	#NIČ!	#NIČ!	#NIČ!	#NIČ!	#NIČ!	#NIČ!	#NIČ!	#NIČ!	#NIČ!	#NIČ!	#NIČ!		#NIČ!	#NIČ!	#NIČ!	#NIČ!	#NIČ!	#NIČ!	34	#NIČ!
191	2	6615866	6	6	7	6	7	6	7	6	6	7	6	6	6	7	6	5	6	4	5	5	5	4	5	4	1	11		2	2	2	5,24	11,00	1,00	34	#NIČ!
192	2	9494566	7	7	7	7	7	7	7	7	5	7	6	6	7	7	6	4	6	5	5	5	5	5	5	5	5	7		2	4	2	5,47	7,00	4,00	34	#NIČ!
193	2	7625229	4	4	4	4	4	4	3	3	3	3	3	4	3	3	3	3	3	3	2	3	3	3	3	3	6	1		2	2	2	2,41	1,00	2,00	34	#NIČ!
194	2	9642450	5	7	7	6	6	6	6	7	7	7	7	4	6	6	6	5	6	3	5	5	3	4	3	3	3	8		3	4	1	5,12	8,00	1,00	34	#NIČ!
195	2	5336564	4	7	4	5	4	3	3	2	3	3	3	3	2	4	5	1	6	3	5	5	3	3	3	3	6	#NIČ!		2	2	2	2,65	#NIČ!	1,00	34	#NIČ!
196	2	7755468	6	6	7	6	6	5	6	5	5	7	6	5	5	7	6	4	7	3	5	5	4	4	4	4	2	9		3	4	2	4,82	9,00	1,00	34	#NIČ!
197	2	2767651	6	7	7	6	6	6	5	5	6	7	6	4	4	6	6	5	6	2	5	5	3	3	3	3	5	8		3	4	2	4,76	8,00	1,00	34	#NIČ!
198	2	571851	6	6	7	6	5	7	4	4	2	7	7	4	4	7	4	4	7	2	2	2	2	2	2	2	2	1		2	3	2	4,35	1,00	2,00	34	#NIČ!
199	2	4556301	7	7	7	6	6	5	6	6	6	6	6	2	6	6	5	3	6	2	4	4	4	4	3	3	1	2		2	2	2	4,65	2,00	5,00	34	#NIČ!
200	#NIČ!	1065955	#NIČ!	#NIČ!	#NIČ!	#NIČ!	#NIČ!	#NIČ!	#NIČ!	#NIČ!	#NIČ!	#NIČ!	#NIČ!	#NIČ!	#NIČ!	#NIČ!	#NIČ!	#NIČ!	#NIČ!	#NIČ!	#NIČ!	#NIČ!	#NIČ!	#NIČ!	#NIČ!	#NIČ!	#NIČ!	#NIČ!		#NIČ!	#NIČ!	#NIČ!	#NIČ!	#NIČ!	#NIČ!	34	#NIČ!
201	2	1660870	7	7	7	7	6	6	6	6	6	6	6	6	6	6	6	6	6	4	5	4	3	4	4	3	1	11		1	5	1	5,24	11,00	1,00	34	#NIČ!
202	2	5106486	4	4	6	4	3	5	3	4	4	3	5	4	3	4	3	2	3	3	3	3	3	2	2	2	2	3		2	2	2	2,76	3,00	5,00	34	#NIČ!
203	2	549821	6	7	7	6	6	6	6	6	6	7	6	7	7	7	7	6	7	4	5	5	4	4	5	5	2	17		2	4	1	5,47	17,00	1,00	34	#NIČ!
204	2	7429647	2	7	4	2	1	1	1	1	1	4	1	1	1	1	4	1	5	1	2	1	1	1	1	1	2	#NIČ!		4	4	2	1,24	#NIČ!	#NIČ!	34	#NIČ!
205	#NIČ!	5498774	#NIČ!	#NIČ!	#NIČ!	#NIČ!	#NIČ!	#NIČ!	#NIČ!	#NIČ!	#NIČ!	#NIČ!	#NIČ!	#NIČ!	#NIČ!	#NIČ!	#NIČ!	#NIČ!	#NIČ!	#NIČ!	#NIČ!	#NIČ!	#NIČ!	#NIČ!	#NIČ!	#NIČ!	#NIČ!	#NIČ!		#NIČ!	#NIČ!	#NIČ!	#NIČ!	#NIČ!	#NIČ!	34	#NIČ!
206	2	5204928	2	7	6	6	3	3	2	2	4	5	1	1	3	1	1	1	2	2	3	2	1	2	1	1	4	#NIČ!		4	1	2	1,94	#NIČ!	#NIČ!	34	#NIČ!
207	2	9528320	4	7	7	5	7	7	7	5	6	7	6	6	5	6	4	4	7	2	4	5	3	5	3	4	2	5		1	4	2	4,88	5,00	3,00	34	#NIČ!
208	2	2026814	4	5	6	6	4	5	5	4	4	6	5	4	6	5	4	1	4	3	3	2	3	3	2	2	2	1		2	2	2	3,59	1,00	2,00	34	#NIČ!
210	#NIČ!	1549111	#NIČ!	#NIČ!	#NIČ!	#NIČ!	#NIČ!	#NIČ!	#NIČ!	#NIČ!	#NIČ!	#NIČ!	#NIČ!	#NIČ!	#NIČ!	#NIČ!	#NIČ!	#NIČ!	#NIČ!	#NIČ!	#NIČ!	#NIČ!	#NIČ!	#NIČ!	#NIČ!	#NIČ!	#NIČ!	#NIČ!		#NIČ!	#NIČ!	#NIČ!	#NIČ!	#NIČ!	#NIČ!	34	#NIČ!
211	2	1665112	6	6	6	6	6	5	6	6	6	6	5	6	6	6	5	4	5	3	5	4	4	4	4	4	1	#NIČ!	specialis	2	4	1	4,65	98,00	7,00	22	1,00
212	2	3678228	6	6	7	6	5	6	6	5	5	6	6	4	5	7	6	4	7	2	4	3	2	2	2	2	4	5		1	4	2	4,71	5,00	3,00	34	#NIČ!
213	2	5944335	6	7	7	6	6	7	7	6	7	5	7	7	6	7	4	6	6	4	4	5	4	4	5	5	8	#NIČ!		4	5	2	5,29	#NIČ!	#NIČ!	34	#NIČ!
214	2	3395804	3	6	6	4	3	3	2	1	2	4	3	2	1	3	3	1	2	1	3	2	2	2	3	3	4	12		3	2	2	1,88	12,00	1,00	34	#NIČ!
215	2	9534265	6	6	7	6	5	6	5	5	7	6	6	6	5	5	4	2	7	3	3	4	3	3	3	3	4	5		1	4	1	4,53	5,00	3,00	34	#NIČ!

216	2	9838321	5	7	6	5	4	6	3	3	4	4	5	5	3	5	3	1	5	3	3	3	3	3	4	2	1	1		2	2	2	3,35	1,00	2,00	34	#NIČ!
217	2	588809	7	6	7	7	6	6	6	6	7	6	6	6	6	6	4	4	7	3	5	5	3	3	2	5	8	#NIČ!	frs	4	2	2	5,06	98,00	7,00	10	1,00
218	2	3429083	6	7	7	7	7	7	7	6	6	6	6	7	7	6	6	6	7	4	4	4	4	3	4	3	5	7		2	4	2	5,53	7,00	4,00	34	#NIČ!
219	2	5378988	6	6	6	6	6	6	6	6	6	6	6	6	6	6	5	5	6	3	3	3	3	3	3	3	4	6		2	2	2	4,88	6,00	3,00	34	#NIČ!
220	2	6607691	6	7	7	6	5	6	4	3	5	4	6	6	7	4	4	3	4	2	5	5	5	3	4	3	3	1		1	5	2	4,12	1,00	2,00	34	#NIČ!
221	#NIČ!	6901490	#NIČ!	#NIČ!	#NIČ!	#NIČ!	#NIČ!	#NIČ!	#NIČ!	#NIČ!	#NIČ!	#NIČ!	#NIČ!	#NIČ!	#NIČ!	#NIČ!	#NIČ!	#NIČ!	#NIČ!	#NIČ!	#NIČ!	#NIČ!	#NIČ!	#NIČ!	#NIČ!	#NIČ!	#NIČ!	#NIČ!		#NIČ!	#NIČ!	#NIČ!	#NIČ!	#NIČ!	#NIČ!	34	#NIČ!
222	2	4684380	6	6	7	6	7	7	7	6	6	7	7	6	7	7	6	6	6	4	4	4	4	4	3	4	6	20		1	5	1	5,47	20,00	1,00	34	#NIČ!
223	2	2717430	6	6	7	7	6	6	6	6	7	7	7	6	5	7	6	5	6	2	3	4	2	3	3	2	4	12		3	4	2	5,24	12,00	1,00	34	#NIČ!
224	2	9534009	6	6	6	5	5	6	4	4	4	5	5	6	4	3	5	1	7	4	5	5	3	4	5	3	6	1		1	5	2	3,82	1,00	2,00	34	#NIČ!
225	1	9517756	6	7	7	7	6	6	#NIČ!	#NIČ!	#NIČ!	#NIČ!	#NIČ!	#NIČ!	#NIČ!	#NIČ!	#NIČ!	#NIČ!	#NIČ!	#NIČ!	#NIČ!	#NIČ!	#NIČ!	#NIČ!	#NIČ!	#NIČ!	#NIČ!	#NIČ!		#NIČ!	#NIČ!	#NIČ!	#NIČ!	#NIČ!	#NIČ!	34	#NIČ!
226	2	8986753	4	5	5	5	6	6	6	5	5	5	5	5	6	6	6	4	6	3	3	4	4	4	3	3	2	16		2	2	2	4,29	16,00	1,00	34	#NIČ!
227	#NIČ!	6380496	#NIČ!	#NIČ!	#NIČ!	#NIČ!	#NIČ!	#NIČ!	#NIČ!	#NIČ!	#NIČ!	#NIČ!	#NIČ!	#NIČ!	#NIČ!	#NIČ!	#NIČ!	#NIČ!	#NIČ!	#NIČ!	#NIČ!	#NIČ!	#NIČ!	#NIČ!	#NIČ!	#NIČ!	#NIČ!	#NIČ!		#NIČ!	#NIČ!	#NIČ!	#NIČ!	#NIČ!	#NIČ!	34	#NIČ!
228	2	4942223	6	7	7	7	7	7	7	7	6	7	7	7	7	7	7	7	7	4	5	5	4	4	2	2	4	5		2	2	2	5,88	5,00	3,00	34	#NIČ!
229	2	5569628	6	7	7	6	6	6	5	6	6	6	6	6	6	6	7	6	5	4	4	4	4	4	4	4	5	1		1	4	2	5,06	1,00	2,00	34	#NIČ!
230	2	3021468	5	7	6	6	6	6	5	5	6	7	7	5	5	7	5	5	7	2	4	4	4	3	4	3	1	15		2	1	2	4,88	15,00	1,00	34	#NIČ!
231	0	8398460	#NIČ!	#NIČ!	#NIČ!	#NIČ!	#NIČ!	#NIČ!	#NIČ!	#NIČ!	#NIČ!	#NIČ!	#NIČ!	#NIČ!	#NIČ!	#NIČ!	#NIČ!	#NIČ!	#NIČ!	#NIČ!	#NIČ!	#NIČ!	#NIČ!	#NIČ!	#NIČ!	#NIČ!	#NIČ!	#NIČ!		#NIČ!	#NIČ!	#NIČ!	#NIČ!	#NIČ!	#NIČ!	34	#NIČ!
232	2	2927895	6	6	7	6	6	7	5	4	5	6	7	6	6	7	5	5	7	2	5	5	5	3	4	4	6	6		1	4	2	4,94	6,00	3,00	34	#NIČ!
233	2	9444097	3	3	6	4	4	5	3	2	1	2	4	3	4	3	2	2	6	2	4	4	4	5	3	2	2	16		2	2	2	2,35	16,00	1,00	34	#NIČ!
234	#NIČ!	8436798	#NIČ!	#NIČ!	#NIČ!	#NIČ!	#NIČ!	#NIČ!	#NIČ!	#NIČ!	#NIČ!	#NIČ!	#NIČ!	#NIČ!	#NIČ!	#NIČ!	#NIČ!	#NIČ!	#NIČ!	#NIČ!	#NIČ!	#NIČ!	#NIČ!	#NIČ!	#NIČ!	#NIČ!	#NIČ!	#NIČ!		#NIČ!	#NIČ!	#NIČ!	#NIČ!	#NIČ!	#NIČ!	34	#NIČ!
235	2	3851700	6	7	6	6	6	5	6	6	6	7	6	6	6	7	6	6	6	4	5	5	4	4	4	4	2	9		3	3	2	5,12	9,00	1,00	34	#NIČ!
236	2	3948106	7	7	7	7	6	6	6	6	7	7	6	5	6	7	7	6	6	3	5	5	5	3	3	3	3	#NIČ!		4	3	2	5,41	#NIČ!	#NIČ!	34	#NIČ!
237	2	8185429	6	6	6	6	6	6	6	6	6	6	6	6	6	6	6	6	6	3	3	3	3	2	2	2	8	#NIČ!		4	2	2	5,00	#NIČ!	#NIČ!	34	#NIČ!
238	#NIČ!	9082827	#NIČ!	#NIČ!	#NIČ!	#NIČ!	#NIČ!	#NIČ!	#NIČ!	#NIČ!	#NIČ!	#NIČ!	#NIČ!	#NIČ!	#NIČ!	#NIČ!	#NIČ!	#NIČ!	#NIČ!	#NIČ!	#NIČ!	#NIČ!	#NIČ!	#NIČ!	#NIČ!	#NIČ!	#NIČ!	#NIČ!		#NIČ!	#NIČ!	#NIČ!	#NIČ!	#NIČ!	#NIČ!	34	#NIČ!
239	2	857849	6	6	6	6	6	6	5	2	2	6	6	1	6	5	4	2	6	3	4	4	3	4	4	4	3	1		1	5	2	3,76	1,00	2,00	34	#NIČ!
240	2	7040764	3	5	7	4	3	7	3	1	1	1	5	6	4	4	4	2	4	2	4	4	3	3	3	2	6	1		2	2	2	2,76	1,00	2,00	34	#NIČ!
241	#NIČ!	2630275	#NIČ!	#NIČ!	#NIČ!	#NIČ!	#NIČ!	#NIČ!	#NIČ!	#NIČ!	#NIČ!	#NIČ!	#NIČ!	#NIČ!	#NIČ!	#NIČ!	#NIČ!	#NIČ!	#NIČ!	#NIČ!	#NIČ!	#NIČ!	#NIČ!	#NIČ!	#NIČ!	#NIČ!	#NIČ!	#NIČ!		#NIČ!	#NIČ!	#NIČ!	#NIČ!	#NIČ!	#NIČ!	34	#NIČ!
242	2	2029082	4	5	5	4	3	6	5	3	5	3	5	5	3	4	3	3	5	2	5	3	3	4	3	3	6	2		1	5	2	3,18	2,00	5,00	34	#NIČ!
243	2	2254587	6	7	7	7	7	7	6	6	6	7	7	7	7	7	7	6	7	4	5	5	5	5	5	5	3	#NIČ!	zd fužin	2	2	2	5,71	98,00	7,00	33	1,00
244	2	5185687	5	7	7	6	6	5	5	5	6	6	4	4	7	5	4	4	7	2	3	3	3	3	2	2	4	3		1	4	2	4,47	3,00	5,00	34	#NIČ!
245	2	9164673	7	7	7	7	7	7	7	7	7	7	7	7	7	7	7	7	7	2	5	5	5	5	5	5	6	1		1	5	1	6,00	1,00	2,00	34	#NIČ!
246	2	266307	6	7	7	7	7	5	5	5	7	5	7	7	7	7	7	5	7	3	4	4	3	4	4	4	3	26		2	2	1	5,35	26,00	1,00	34	#NIČ!
247	2	3837514	7	7	7	7	7	4	5	5	4	6	5	7	7	7	4	1	7	3	3	3	3	2	2	2	1	1		2	2	2	4,71	1,00	2,00	34	#NIČ!
248	2	8388649	5	6	6	5	5	5	5	4	6	6	4	6	6	5	6	4	5	3	4	4	4	4	4	3	4	14		2	4	2	4,24	14,00	1,00	34	#NIČ!
249	2	430703	7	4	7	1	3	1	3	4	5	3	3	4	3	3	5	1	5	3	2	3	3	2	3	2	2	#NIČ!		4	2	2	2,65	#NIČ!	#NIČ!	34	#NIČ!
250	2	6987569	6	7	7	7	6	6	6	6	6	7	6	6	6	7	7	7	7	4	5	4	5	4	4	4	6	21		1	6	1	5,47	21,00	1,00	34	#NIČ!
251	2	3645735	5	3	4	4	4	7	4	4	7	7	7	4	4	7	3	1	7	3	3	3	3	3	3	3	1	#NIČ!		4	#NIČ!	2	3,82	#NIČ!	#NIČ!	34	#NIČ!
252	#NIČ!	7265970	#NIČ!	#NIČ!	#NIČ!	#NIČ!	#NIČ!	#NIČ!	#NIČ!	#NIČ!	#NIČ!	#NIČ!	#NIČ!	#NIČ!	#NIČ!	#NIČ!	#NIČ!	#NIČ!	#NIČ!	#NIČ!	#NIČ!	#NIČ!	#NIČ!	#NIČ!	#NIČ!	#NIČ!	#NIČ!	#NIČ!		#NIČ!	#NIČ!	#NIČ!	#NIČ!	#NIČ!	#NIČ!	34	#NIČ!
253	2	5392646	6	7	4	6	5	6	5	5	7	7	5	7	4	6	6	3	7	5	4	5	5	5	5	5	3	1		2	6	2	4,65	1,00	2,00	34	#NIČ!
254	2	5165321	6	7	6	6	6	5	7	6	7	7	6	6	7	7	5	4	5	3	4	5	3	3	3	3	1	11		3	3	2	5,06	11,00	1,00	34	#NIČ!
255	2	9648667	6	7	7	7	6	7	7	6	7	7	7	7	6	5	6	4	6	4	5	5	4	3	2	3	1	6		1	3	2	5,35	6,00	3,00	34	#NIČ!
256	#NIČ!	2747373	#NIČ!	#NIČ!	#NIČ!	#NIČ!	#NIČ!	#NIČ!	#NIČ!	#NIČ!	#NIČ!	#NIČ!	#NIČ!	#NIČ!	#NIČ!	#NIČ!	#NIČ!	#NIČ!	#NIČ!	#NIČ!	#NIČ!	#NIČ!	#NIČ!	#NIČ!	#NIČ!	#NIČ!	#NIČ!	#NIČ!		#NIČ!	#NIČ!	#NIČ!	#NIČ!	#NIČ!	#NIČ!	34	#NIČ!
258	2	4790865	5	7	7	6	6	6	5	5	4	7	7	7	7	6	6	5	7	3	3	3	3	3	3	3	6	6		2	2	2	5,06	6,00	3,00	34	#NIČ!
260	2	4188439	6	7	7	7	7	7	7	7	7	7	7	7	7	6	5	6	7	3	3	3	3	3	3	3	3	7		2	2	2	5,71	7,00	4,00	34	#NIČ!
261	2	3805575	7	7	7	7	7	7	7	7	7	7	7	7	7	7	7	7	7	5	5	5	5	5	5	5	8	#NIČ!	uprava	5	4	1	6,00	98,00	7,00	26	1,00
262	2	6462557	6	6	7	6	6	5	6	6	7	7	7	4	6	7	6	4	6	3	3	3	3	3	5	5	3	2		2	3	2	5,00	2,00	5,00	34	#NIČ!
263	2	5712207	5	7	7	5	7	7	6	5	6	6	6	6	6	6	4	5	6	3	3	3	5	5	5	5	6	6		2	2	2	4,88	6,00	3,00	34	#NIČ!
264	2	896060	7	7	7	7	7	7	4	3	3	6	6	6	6	6	5	6	7	5	4	5	3	3	2	3	3	6		1	4	2	4,88	6,00	3,00	34	#NIČ!
265	0	5092628	#NIČ!	#NIČ!	#NIČ!	#NIČ!	#NIČ!	#NIČ!	#NIČ!	#NIČ!	#NIČ!	#NIČ!	#NIČ!	#NIČ!	#NIČ!	#NIČ!	#NIČ!	#NIČ!	#NIČ!	#NIČ!	#NIČ!	#NIČ!	#NIČ!	#NIČ!	#NIČ!	#NIČ!	#NIČ!	#NIČ!		#NIČ!	#NIČ!	#NIČ!	#NIČ!	#NIČ!	#NIČ!	34	#NIČ!
266	2	2774963	6	6	6	5	5	6	6	6	6	6	6	6	6	6	6	5	6	3	4	4	3	4	3	3	2	9		3	3	2	4,82	9,00	1,00	34	#NIČ!
267	2	8596932	6	7	7	7	7	6	7	7	7	7	4	5	5	7	7	4	7	5	5	5	5	4	4	3	3	8		3	#NIČ! visokoš	2	5,29	8,00	1,00	34	#NIČ!
268	2	4659772	6	7	7	7	7	7	7	7	7	7	7	6	6	7	5	4	6	4	5	5	4	4	4	4	3	2		2	4	2	5,47	2,00	5,00	34	#NIČ!
269	2	7508064	4	4	6	5	5	6	4	5	6	6	6	6	5	5	5	4	6	3	4	5	4	4	4	3	1	2		2	4	1	4,18	2,00	5,00	34	#NIČ!
270	2	3561005	6	7	6	5	5	6	5	5	6	6	6	6	6	6	5	4	6	3	2	4	3	3	2	2	1	12		3	4	2	4,65	12,00	1,00	34	#NIČ!
271	1	5280833	6	7	7	6	6	7	7	6	6	6	7	5	7	7	6	4	6	2	5	3	3	4	2	2	#NIČ!	#NIČ!		#NIČ!	#NIČ!	#NIČ!	5,24	#NIČ!	#NIČ!	34	#NIČ!

272	2	5721149	6	6	7	6	6	6	5	6	6	6	5	5	6	6	6	1	6	2	4	4	4	4	3	3	4	1		2		4	2	4,59	1,00	2,00	34	#NIČ!
273	#NIČ!	2763249	#NIČ!	#NIČ!	#NIČ!	#NIČ!	#NIČ!	#NIČ!	#NIČ!	#NIČ!	#NIČ!	#NIČ!	#NIČ!	#NIČ!	#NIČ!	#NIČ!	#NIČ!	#NIČ!	#NIČ!	#NIČ!	#NIČ!	#NIČ!	#NIČ!	#NIČ!	#NIČ!	#NIČ!	#NIČ!	#NIČ!		#NIČ!		#NIČ!	#NIČ!	#NIČ!	#NIČ!	#NIČ!	34	#NIČ!
274	#NIČ!	6652797	#NIČ!	#NIČ!	#NIČ!	#NIČ!	#NIČ!	#NIČ!	#NIČ!	#NIČ!	#NIČ!	#NIČ!	#NIČ!	#NIČ!	#NIČ!	#NIČ!	#NIČ!	#NIČ!	#NIČ!	#NIČ!	#NIČ!	#NIČ!	#NIČ!	#NIČ!	#NIČ!	#NIČ!	#NIČ!	#NIČ!		#NIČ!		#NIČ!	#NIČ!	#NIČ!	#NIČ!	#NIČ!	34	#NIČ!
275	2	4974238	6	6	7	6	6	7	6	6	7	6	7	6	7	7	6	4	6	2	5	3	3	3	2	2	3	11		3		4	2	5,24	11,00	1,00	34	#NIČ!
276	2	4912798	4	4	6	5	6	6	5	5	4	4	4	3	3	6	4	2	4	2	2	2	2	3	2	2	6	2		2		4	2	3,41	2,00	5,00	34	#NIČ!
277	2	9641275	5	4	7	5	4	3	4	2	4	4	3	6	4	6	5	5	6	1	3	3	3	2	3	3	6	#NIČ!		1		5	1	3,53	#NIČ!	1,00	34	#NIČ!
278	2	3467985	6	7	7	6	6	6	6	6	6	7	6	6	6	6	5	5	6	3	3	3	3	3	3	3	6	1		1		5	1	5,06	1,00	2,00	34	#NIČ!
279	2	8416099	6	7	6	5	6	6	6	6	6	6	6	6	6	6	5	2	7	2	5	5	4	4	3	3	1	6		1		5	2	4,76	6,00	3,00	34	#NIČ!
280	2	1676539	5	6	6	6	6	6	5	6	6	7	6	7	6	5	6	6	6	4	3	3	3	3	4	3	4	2		1		5	2	4,94	2,00	5,00	34	#NIČ!
281	2	3134398	5	6	7	6	6	6	6	4	5	6	5	6	6	6	5	5	7	5	5	5	3	4	3	2	6	#NIČ!		3		2	2	4,71	#NIČ!	1,00	34	#NIČ!
282	2	642372	6	6	7	7	6	6	6	7	7	7	7	7	6	6	5	6	6	3	5	5	4	4	3	3	3	8		3		4	2	5,35	8,00	1,00	34	#NIČ!
283	#NIČ!	3808669	#NIČ!	#NIČ!	#NIČ!	#NIČ!	#NIČ!	#NIČ!	#NIČ!	#NIČ!	#NIČ!	#NIČ!	#NIČ!	#NIČ!	#NIČ!	#NIČ!	#NIČ!	#NIČ!	#NIČ!	#NIČ!	#NIČ!	#NIČ!	#NIČ!	#NIČ!	#NIČ!	#NIČ!	#NIČ!	#NIČ!		#NIČ!		#NIČ!	#NIČ!	#NIČ!	#NIČ!	#NIČ!	34	#NIČ!
284	#NIČ!	7116229	#NIČ!	#NIČ!	#NIČ!	#NIČ!	#NIČ!	#NIČ!	#NIČ!	#NIČ!	#NIČ!	#NIČ!	#NIČ!	#NIČ!	#NIČ!	#NIČ!	#NIČ!	#NIČ!	#NIČ!	#NIČ!	#NIČ!	#NIČ!	#NIČ!	#NIČ!	#NIČ!	#NIČ!	#NIČ!	#NIČ!		#NIČ!		#NIČ!	#NIČ!	#NIČ!	#NIČ!	#NIČ!	34	#NIČ!
285	2	4155137	6	7	5	5	6	6	6	6	5	6	6	6	5	6	5	4	5	4	4	3	3	3	3	3	3	6		2		2	2	4,59	6,00	3,00	34	#NIČ!
286	2	9426997	6	7	7	6	6	6	6	6	7	6	6	6	7	6	6	5	6	3	4	4	5	4	5	4	3	6		1		4	2	5,18	6,00	3,00	34	#NIČ!
287	2	4669575	6	6	6	6	7	5	6	6	6	7	7	6	7	6	5	5	6	4	5	5	5	5	4	3	6	5		3		2	1	5,06	5,00	3,00	34	#NIČ!
288	2	5066886	6	6	6	6	6	5	6	6	5	6	5	6	6	6	6	4	5	4	4	4	4	4	5	5	3	1		2		4	2	4,65	1,00	2,00	34	#NIČ!
289	2	1325705	6	5	7	6	4	7	4	4	4	6	7	1	3	7	6	1	7	3	4	4	3	3	3	3	4	1		2		2	2	4,00	1,00	2,00	34	#NIČ!
290	2	1427868	7	7	7	7	7	7	7	6	7	7	7	6	6	7	6	6	7	4	5	5	5	5	5	5	3	5		2		2	2	5,71	5,00	3,00	34	#NIČ!
291	2	3162226	4	7	6	5	5	4	5	6	4	6	5	5	5	5	5	5	5	2	3	2	2	2	3	2	6	5		1		4	2	4,12	5,00	3,00	34	#NIČ!
292	2	1527526	1	7	3	4	5	5	3	2	3	5	6	4	5	5	4	6	4	3	3	3	4	4	3	3	2	3		2		2	2	3,24	3,00	5,00	34	#NIČ!
293	2	8150950	5	7	7	7	7	7	7	1	4	7	7	7	7	7	7	4	7	2	4	3	3	3	3	2	3	2		1		5	1	5,18	2,00	5,00	34	#NIČ!
294	2	6172175	6	6	6	6	7	5	6	5	6	7	6	6	7	7	6	5	7	3	4	3	3	3	1	1	4	20		2		2	1	5,12	20,00	1,00	34	#NIČ!
295	2	6408025	5	5	6	5	4	5	4	4	4	4	5	6	6	6	5	3	6	4	5	5	5	5	5	5	3	15		3		4	2	3,88	15,00	1,00	34	#NIČ!
296	2	3523600	3	4	7	4	4	7	4	3	4	4	6	7	6	7	4	7	7	4	3	3	3	3	5	3	3	1		1		5	2	4,18	1,00	2,00	34	#NIČ!
297	#NIČ!	8393925	#NIČ!	#NIČ!	#NIČ!	#NIČ!	#NIČ!	#NIČ!	#NIČ!	#NIČ!	#NIČ!	#NIČ!	#NIČ!	#NIČ!	#NIČ!	#NIČ!	#NIČ!	#NIČ!	#NIČ!	#NIČ!	#NIČ!	#NIČ!	#NIČ!	#NIČ!	#NIČ!	#NIČ!	#NIČ!	#NIČ!		#NIČ!		#NIČ!	#NIČ!	#NIČ!	#NIČ!	#NIČ!	34	#NIČ!
298	2	1398827	3	5	2	4	3	3	4	2	2	5	2	2	2	4	2	2	4	2	3	2	2	3	2	2	4	1		2		2	2	2,00	1,00	2,00	34	#NIČ!
299	2	1812360	4	5	7	5	5	6	7	6	1	7	7	5	5	4	4	3	5	1	3	2	2	2	2	2	6	8		#NIČ!		4	2	4,06	8,00	1,00	34	#NIČ!
300	2	4865318	4	4	5	4	6	6	6	6	6	6	3	5	6	7	6	5	5	2	2	2	1	1	3	2	2	1		2		4	2	4,29	1,00	2,00	34	#NIČ!
301	#NIČ!	8889512	#NIČ!	#NIČ!	#NIČ!	#NIČ!	#NIČ!	#NIČ!	#NIČ!	#NIČ!	#NIČ!	#NIČ!	#NIČ!	#NIČ!	#NIČ!	#NIČ!	#NIČ!	#NIČ!	#NIČ!	#NIČ!	#NIČ!	#NIČ!	#NIČ!	#NIČ!	#NIČ!	#NIČ!	#NIČ!	#NIČ!		#NIČ!		#NIČ!	#NIČ!	#NIČ!	#NIČ!	#NIČ!	34	#NIČ!
302	2	9851606	6	4	7	4	4	7	4	4	6	6	7	4	7	7	3	6	7	5	5	4	5	3	3	4	2	8		3		4	2	4,47	8,00	1,00	34	#NIČ!
303	2	2589493	6	7	6	7	7	7	6	6	7	7	7	6	7	7	6	6	6	3	5	5	3	5	3	3	3	#NIČ!	telefonij	4		2	2	5,53	98,00	7,00	25	1,00
304	2	3392646	7	7	7	7	7	6	6	7	7	7	6	6	7	7	7	7	7	3	5	3	2	3	3	2	3	5		3		2	2	5,76	5,00	3,00	34	#NIČ!
305	2	9194755	6	7	6	6	6	6	6	6	6	6	6	6	6	6	6	2	5	3	4	4	4	4	3	3	4	11		#NIČ!		4	2	4,76	11,00	1,00	34	#NIČ!
306	2	5795835	6	7	7	7	6	6	6	6	6	6	6	6	6	7	6	6	7	3	5	5	4	4	5	4	6	15		3		4	2	5,29	15,00	1,00	34	#NIČ!
307	2	1394909	6	7	7	6	6	5	6	6	5	7	6	6	5	6	6	2	7	2	5	5	3	3	3	3	6	15		2		2	2	4,82	15,00	1,00	34	#NIČ!
308	2	9587040	4	5	5	5	5	6	5	4	3	5	5	3	3	4	4	1	7	1	3	3	3	3	3	3	6	18		3		2	2	3,35	18,00	3,00	34	#NIČ!
309	#NIČ!	3750475	#NIČ!	#NIČ!	#NIČ!	#NIČ!	#NIČ!	#NIČ!	#NIČ!	#NIČ!	#NIČ!	#NIČ!	#NIČ!	#NIČ!	#NIČ!	#NIČ!	#NIČ!	#NIČ!	#NIČ!	#NIČ!	#NIČ!	#NIČ!	#NIČ!	#NIČ!	#NIČ!	#NIČ!	#NIČ!	#NIČ!		#NIČ!		#NIČ!	#NIČ!	#NIČ!	#NIČ!	#NIČ!	34	#NIČ!
310	2	9991255	4	7	7	7	6	6	6	4	5	5	5	7	6	7	4	3	7	3	5	4	4	3	3	3	6	20		2		2	2	4,65	20,00	1,00	34	#NIČ!
311	2	8704850	2	5	7	5	6	7	6	5	2	5	5	4	4	7	4	6	7	2	4	5	3	3	3	3	3	6		1		4	2	4,12	6,00	3,00	34	#NIČ!
312	2	3550485	6	6	7	6	6	7	6	6	6	6	7	7	7	6	5	4	6	4	5	5	4	4	4	4	6	2		1		5	2	5,12	2,00	5,00	34	#NIČ!
313	2	1637875	6	5	7	6	6	6	6	5	7	6	5	5	6	6	5	4	7	2	5	5	2	5	5	4	4	2		1		4	2	4,71	2,00	5,00	34	#NIČ!
314	2	7537919	4	4	7	7	7	6	6	6	5	6	6	6	6	6	4	4	7	3	4	3	3	3	3	3	2	8		3		5	2	4,71	8,00	1,00	34	#NIČ!
315	2	2775971	6	6	6	6	5	6	5	5	6	6	5	5	4	6	6	3	6	2	5	5	5	5	5	3	4	3		1		4	2	4,41	3,00	5,00	34	#NIČ!
316	2	1266099	6	6	7	6	6	7	6	5	7	6	6	7	7	6	5	6	7	3	3	4	4	4	5	3	2	1		2		4	1	5,24	1,00	2,00	34	#NIČ!
317	2	8002580	5	6	7	6	5	7	6	5	7	6	6	7	7	6	4	5	7	3	4	5	3	4	5	5	2	1		1		4	2	5,00	1,00	2,00	34	#NIČ!
318	2	6214605	7	7	7	7	7	7	7	7	7	7	7	7	7	7	7	2	7	5	5	5	5	5	5	5	7	6		1		4	2	5,71	6,00	3,00	34	#NIČ!
319	2	7065286	6	7	7	7	7	6	7	7	7	7	5	6	6	7	5	6	6	3	4	4	3	4	4	3	2	7		2		2	2	5,41	7,00	4,00	34	#NIČ!
320	2	6682616	7	7	7	7	6	7	7	7	7	7	7	7	7	7	7	5	7	4	5	5	4	4	4	4	6	6		2		4	2	5,82	6,00	3,00	34	#NIČ!
321	2	2217223	4	4	5	4	4	4	4	4	4	4	3	3	3	3	4	3	4	2	3	3	2	3	3	2	6	21		2		4	2	2,76	21,00	1,00	34	#NIČ!
322	2	1038265	6	7	6	6	6	6	5	5	3	5	6	7	7	7	5	1	7	2	5	4	1	3	4	2	6	1		1		4	2	4,59	1,00	2,00	34	#NIČ!
323	2	8539658	7	7	7	7	7	6	7	7	7	7	7	7	7	7	6	6	7	3	3	4	4	4	3	3	6	14		2		5	1	5,82	14,00	1,00	34	#NIČ!
324	2	9583493	4	7	7	4	4	5	5	3	2	5	5	4	4	5	3	4	5	1	4	4	3	3	4	4	7	#NIČ!	SNMP	1		5	2	3,47	98,00	7,00	21	1,00
325	2	2298491	6	7	7	7	7	6	7	6	6	7	7	7	7	7	4	4	3	3	3	4	3	5	5	5	7	#NIČ!	ambular	#NIČ!	hisni c	5	2	5,18	98,00	7,00	4	1,00

326	0	2741978	4	4	5	3	4	3	2	4	4	4	3	4	4	4	4	#NIČ!	5	1	2	2	2	#NIČ!	3	2	#NIČ!	#NIČ!		#NIČ!	#NIČ!	#NIČ!	#NIČ!	#NIČ!	#NIČ!	34	#NIČ!
327	2	6814416	5	5	7	3	5	5	5	5	5	4	4	4	5	4	4	4	5	2	3	4	3	2	2	3	6	#NIČ!		2	4	2	3,65	#NIČ!	#NIČ!	34	#NIČ!
328	2	5846145	3	7	7	5	6	7	5	5	7	6	7	6	7	7	5	5	7	1	4	5	3	3	2	1	2	9		3	4	2	5,00	9,00	1,00	34	#NIČ!
329	2	8787478	7	7	7	7	7	7	7	7	7	7	7	7	7	7	7	7	7	3	4	5	4	3	2	2	4	7		2	4	2	6,00	7,00	4,00	34	#NIČ!
330	2	6398955	7	7	7	7	7	6	7	6	6	7	6	6	6	7	6	3	7	4	5	5	3	4	4	4	6	7		2	4	2	5,35	7,00	4,00	34	#NIČ!
331	2	5632340	6	6	6	6	6	6	6	6	6	7	6	6	6	7	6	5	6	5	5	4	3	3	4	3	2	9		3	4	2	5,06	9,00	1,00	34	#NIČ!
332	2	8964836	7	7	7	7	7	7	7	7	7	7	7	7	7	7	7	1	7	3	3	3	3	3	3	3	4	7		2	4	2	5,65	7,00	4,00	34	#NIČ!
333	2	7927159	7	7	6	7	6	6	6	5	5	5	5	6	6	7	5	5	5	4	5	5	5	5	3	4	3	1		2	2	2	4,82	1,00	2,00	34	#NIČ!
334	0	2741287	#NIČ!	#NIČ!	#NIČ!	#NIČ!	#NIČ!	#NIČ!	#NIČ!	#NIČ!	#NIČ!	#NIČ!	#NIČ!	#NIČ!	#NIČ!	#NIČ!	#NIČ!	#NIČ!	#NIČ!	#NIČ!	#NIČ!	#NIČ!	#NIČ!	#NIČ!	#NIČ!	#NIČ!	#NIČ!	#NIČ!		#NIČ!	#NIČ!	#NIČ!	#NIČ!	#NIČ!	#NIČ!	34	#NIČ!
335	2	9924961	5	6	6	5	6	4	5	4	6	6	5	6	5	5	4	5	5	3	5	5	4	4	4	3	2	9		3	4	2	4,18	9,00	1,00	34	#NIČ!
336	2	1400944	5	5	7	5	5	5	5	5	5	5	5	5	5	5	5	3	5	3	3	3	3	3	3	3	1	1		1	5	2	4,00	1,00	2,00	34	#NIČ!
337	2	7229837	5	6	6	5	5	5	5	5	5	5	5	5	5	5	5	7	7	4	3	3	3	3	4	3	3	1		1	5	1	4,35	1,00	2,00	34	#NIČ!
338	#NIČ!	1946355	#NIČ!	#NIČ!	#NIČ!	#NIČ!	#NIČ!	#NIČ!	#NIČ!	#NIČ!	#NIČ!	#NIČ!	#NIČ!	#NIČ!	#NIČ!	#NIČ!	#NIČ!	#NIČ!	#NIČ!	#NIČ!	#NIČ!	#NIČ!	#NIČ!	#NIČ!	#NIČ!	#NIČ!	#NIČ!	#NIČ!		#NIČ!	#NIČ!	#NIČ!	#NIČ!	#NIČ!	#NIČ!	34	#NIČ!
339	2	8042263	6	6	6	6	4	4	4	3	6	6	6	6	6	6	5	3	3	1	4	3	3	2	4	3	6	7		2	4	2	4,06	7,00	4,00	34	#NIČ!
340	2	4372252	7	6	7	6	5	7	5	4	1	5	6	4	3	7	4	7	5	3	4	4	3	3	3	3	6	11		2	3	2	4,24	11,00	1,00	34	#NIČ!
341	2	7734472	7	7	7	7	7	7	6	7	6	7	7	7	7	7	6	6	7	3	5	5	4	4	3	3	4	6		2	2	2	5,76	6,00	3,00	34	#NIČ!
342	2	5555602	5	6	6	6	6	4	6	5	6	7	4	6	6	6	4	4	6	3	5	4	3	4	3	3	6	18		3	2	2	4,47	18,00	3,00	34	#NIČ!
343	2	4574597	6	6	7	7	6	6	6	6	7	7	6	6	6	7	7	5	7	3	5	5	3	3	3	3	5	#NIČ!		4	3	2	5,35	#NIČ!	#NIČ!	34	#NIČ!
344	2	6206178	7	7	7	7	7	7	6	6	6	7	7	6	7	7	6	7	7	4	5	5	5	4	5	4	6	5		1	4	2	5,71	5,00	3,00	34	#NIČ!
345	2	7307098	6	7	7	7	7	7	6	6	6	7	7	7	6	7	6	6	7	2	5	4	4	5	4	4	1	6		2	2	2	5,59	6,00	3,00	34	#NIČ!
346	2	7916959	6	7	7	7	7	7	7	6	6	6	7	7	7	7	7	6	6	3	5	5	5	4	3	3	4	7		2	4	2	5,65	7,00	4,00	34	#NIČ!
347	2	7663498	6	7	7	5	6	6	6	5	5	7	5	3	7	5	4	4	4	3	4	3	2	3	2	3	1	8		3	4	2	4,41	8,00	1,00	34	#NIČ!
348	1	4566616	5	7	7	6	6	4	5	4	4	7	7	3	7	6	3	1	5	1	5	3	2	3	2	1	#NIČ!	#NIČ!		#NIČ!	#NIČ!	#NIČ!	4,12	#NIČ!	#NIČ!	34	#NIČ!
349	2	9842585	6	7	6	6	5	5	6	6	6	7	6	6	6	6	7	5	7	4	3	4	4	4	5	4	1	1		2	2	2	5,06	1,00	2,00	34	#NIČ!
350	2	5513078	6	6	6	6	6	5	6	5	5	6	5	6	5	7	5	5	5	4	5	5	4	5	5	4	2	12		3	2	2	4,59	12,00	1,00	34	#NIČ!
351	2	8037634	4	6	7	6	5	6	4	4	4	5	7	4	7	7	3	6	7	3	5	5	4	4	3	3	6	1		2	2	2	4,41	1,00	2,00	34	#NIČ!
352	#NIČ!	3648939	#NIČ!	#NIČ!	#NIČ!	#NIČ!	#NIČ!	#NIČ!	#NIČ!	#NIČ!	#NIČ!	#NIČ!	#NIČ!	#NIČ!	#NIČ!	#NIČ!	#NIČ!	#NIČ!	#NIČ!	#NIČ!	#NIČ!	#NIČ!	#NIČ!	#NIČ!	#NIČ!	#NIČ!	#NIČ!	#NIČ!		#NIČ!	#NIČ!	#NIČ!	#NIČ!	#NIČ!	#NIČ!	34	#NIČ!
353	2	4131793	6	6	7	6	5	7	5	5	6	4	6	7	6	7	4	2	7	2	4	4	5	3	5	5	6	7		2	4	2	4,65	7,00	4,00	34	#NIČ!
354	2	9712146	6	7	7	6	6	6	6	6	7	7	6	6	6	7	7	1	7	1	5	5	5	4	5	5	6	7		2	4	2	5,12	7,00	4,00	34	#NIČ!
355	2	6165353	7	4	7	5	2	1	2	2	2	3	2	3	3	5	6	2	5	2	3	3	2	2	3	3	3	1		2	2	2	2,59	1,00	2,00	34	#NIČ!
356	2	1690327	6	7	7	7	6	7	7	4	5	7	6	7	7	7	4	5	3	3	3	3	3	3	3	4	6	7		2	5	2	5,00	7,00	4,00	34	#NIČ!
357	2	9955578	6	7	7	7	6	7	6	7	7	7	7	7	7	7	7	7	7	3	5	4	4	4	4	4	8	#NIČ!	uprava	4	2	2	5,82	98,00	7,00	26	1,00
358	2	4706907	4	5	5	4	5	5	4	4	5	5	5	4	4	5	4	4	4	3	2	3	2	2	2	3	4	7		2	2	2	3,47	7,00	4,00	34	#NIČ!
359	2	3667803	5	6	7	6	6	2	5	5	5	7	5	5	5	5	4	2	6	4	4	4	3	3	2	2	6	18		3	2	2	4,06	18,00	3,00	34	#NIČ!
360	2	4218295	6	7	6	6	6	5	5	5	6	6	6	6	6	6	6	5	6	3	3	4	4	4	4	4	6	3		2	4	1	4,82	3,00	5,00	34	#NIČ!
361	2	88063	6	6	7	6	6	7	6	6	6	7	7	7	6	6	6	4	6	4	5	4	4	4	5	4	6	7		2	4	2	5,18	7,00	4,00	34	#NIČ!
362	2	7785433	5	6	7	6	5	7	7	7	7	7	7	5	5	7	4	3	7	3	3	3	4	4	4	4	2	1		2	2	2	5,00	1,00	2,00	34	#NIČ!
363	2	8662978	5	6	7	4	4	6	5	4	5	6	6	4	6	6	5	6	7	2	3	4	3	3	2	2	4	3		5	5	1	4,41	3,00	5,00	34	#NIČ!
364	2	9958588	5	7	7	7	7	5	7	7	6	7	6	5	7	7	3	5	7	3	3	3	3	4	3	3	4	7		2	4	2	5,18	7,00	4,00	34	#NIČ!
365	2	3804006	5	7	7	7	6	7	6	6	6	6	6	6	6	7	6	6	7	3	4	4	3	3	2	3	2	21		2	2	2	5,29	21,00	1,00	34	#NIČ!
367	2	9144266	4	5	6	4	4	6	5	4	5	7	4	7	5	6	6	6	6	3	3	3	3	3	3	3	1	8		3	4	2	4,29	8,00	1,00	34	#NIČ!
368	2	4309315	5	6	7	7	7	6	3	6	7	6	5	7	7	7	7	1	6	1	1	3	2	3	3	1	4	7		2	2	1	4,88	7,00	4,00	34	#NIČ!
369	2	4113775	2	5	5	3	2	2	2	1	2	2	2	2	2	2	1	2	2	1	3	3	2	2	1	1	4	5		2	#NIČ!	2	1,29	5,00	3,00	34	#NIČ!
370	2	7640931	5	6	7	6	6	5	4	4	5	4	3	4	5	4	5	2	5	4	5	5	5	3	5	4	3	1		2	5	2	3,71	1,00	2,00	34	#NIČ!
371	2	5863331	6	7	7	6	6	7	6	4	4	6	4	4	6	6	5	3	5	2	4	4	3	2	4	3	7	#NIČ!	snmp	1	5	2	4,41	98,00	7,00	19	1,00
372	2	6393864	6	7	6	6	6	7	6	6	6	6	6	6	6	6	6	4	6	2	4	5	4	4	4	4	7	#NIČ!		1	4	2	5,00	#NIČ!	#NIČ!	34	#NIČ!
373	2	4379325	6	7	7	6	6	6	5	5	5	5	4	6	6	6	6	4	6	4	5	5	5	4	4	3	4	5		5	4	1	4,65	5,00	3,00	34	#NIČ!
374	#NIČ!	2715034	#NIČ!	#NIČ!	#NIČ!	#NIČ!	#NIČ!	#NIČ!	#NIČ!	#NIČ!	#NIČ!	#NIČ!	#NIČ!	#NIČ!	#NIČ!	#NIČ!	#NIČ!	#NIČ!	#NIČ!	#NIČ!	#NIČ!	#NIČ!	#NIČ!	#NIČ!	#NIČ!	#NIČ!	#NIČ!	#NIČ!		#NIČ!	#NIČ!	#NIČ!	#NIČ!	#NIČ!	#NIČ!	34	#NIČ!
376	#NIČ!	437196	#NIČ!	#NIČ!	#NIČ!	#NIČ!	#NIČ!	#NIČ!	#NIČ!	#NIČ!	#NIČ!	#NIČ!	#NIČ!	#NIČ!	#NIČ!	#NIČ!	#NIČ!	#NIČ!	#NIČ!	#NIČ!	#NIČ!	#NIČ!	#NIČ!	#NIČ!	#NIČ!	#NIČ!	#NIČ!	#NIČ!		#NIČ!	#NIČ!	#NIČ!	#NIČ!	#NIČ!	#NIČ!	34	#NIČ!
377	2	4040877	6	7	6	6	7	6	6	6	7	7	7	6	7	7	6	5	4	4	5	5	5	5	4	3	6	12		3	4	2	5,24	12,00	1,00	34	#NIČ!
378	2	8892796	5	6	6	5	5	6	5	5	5	5	5	5	6	6	5	4	6	3	5	5	4	4	4	4	3	3		1	5	2	4,29	3,00	5,00	34	#NIČ!
379	2	2341350	4	5	5	4	4	6	4	4	4	4	7	6	5	6	5	4	6	3	4	5	3	3	3	3	4	1		1	5	2	3,88	1,00	2,00	34	#NIČ!
381	2	5028361	6	6	7	6	6	7	6	6	6	6	7	7	7	7	7	7	7	5	4	5	5	5	4	4	1	8		3	4	1	5,53	8,00	1,00	34	#NIČ!
382	2	8117756	7	7	7	7	6	7	6	6	7	5	7	6	6	7	3	1	7	5	5	5	5	5	5	2	2	21		#NIČ!	2	2	5,00	21,00	1,00	34	#NIČ!

384	2	5503699	7	6	7	6	5	5	5	5	6	7	6	7	6	7	7	6	7	3	4	5	4	3	4	4	5	1		2	4		1	5,18	1,00	2,00	34	#NIČ!
385	2	3165227	5	7	7	4	5	4	5	4	6	5	3	2	5	5	3	2	5	3	5	5	3	2	3	2	4	8		3	3		2	3,53	8,00	1,00	34	#NIČ!
386	2	9315039	7	7	7	6	7	7	7	7	7	7	7	7	6	7	6	6	6	5	5	5	5	5	5	5	2	9		3	4		2	5,71	9,00	1,00	34	#NIČ!
387	#NIČ!	7079513	#NIČ!	#NIČ!	#NIČ!	#NIČ!	#NIČ!	#NIČ!	#NIČ!	#NIČ!	#NIČ!	#NIČ!	#NIČ!	#NIČ!	#NIČ!	#NIČ!	#NIČ!	#NIČ!	#NIČ!	#NIČ!	#NIČ!	#NIČ!	#NIČ!	#NIČ!	#NIČ!	#NIČ!	#NIČ!	#NIČ!		#NIČ!	#NIČ!		#NIČ!	#NIČ!	#NIČ!	#NIČ!	34	#NIČ!
388	2	7452448	5	5	5	4	4	4	4	5	5	5	5	5	5	5	5	3	4	3	3	3	3	3	3	3	1	4		2	2		2	3,59	4,00	6,00	34	#NIČ!
389	2	6023701	6	6	7	6	7	7	6	7	7	7	7	7	6	7	7	6	7	5	5	5	5	5	4	4	3	#NIČ!	sterilizac	2	2		2	5,65	98,00	7,00	23	1,00
390	2	7761162	6	7	7	7	7	7	7	6	7	7	6	7	6	7	6	5	7	3	5	5	4	4	3	3	2	6		1	4		2	5,59	6,00	3,00	34	#NIČ!
391	2	734709	5	6	6	5	5	6	5	4	5	6	6	5	7	6	3	5	6	3	3	3	3	3	3	3	3	11		3	4		2	4,35	11,00	1,00	34	#NIČ!
392	2	390058	4	7	7	7	4	4	4	4	4	5	4	4	5	5	5	4	4	3	4	3	3	3	3	3	8	#NIČ!		4	4		2	3,76	#NIČ!	#NIČ!	34	#NIČ!
393	2	9746162	7	7	7	7	7	6	7	7	6	7	6	6	7	7	7	5	7	4	5	5	4	4	4	3	1	6		1	4		2	5,65	6,00	3,00	34	#NIČ!
394	#NIČ!	7560637	#NIČ!	#NIČ!	#NIČ!	#NIČ!	#NIČ!	#NIČ!	#NIČ!	#NIČ!	#NIČ!	#NIČ!	#NIČ!	#NIČ!	#NIČ!	#NIČ!	#NIČ!	#NIČ!	#NIČ!	#NIČ!	#NIČ!	#NIČ!	#NIČ!	#NIČ!	#NIČ!	#NIČ!	#NIČ!	#NIČ!		#NIČ!	#NIČ!		#NIČ!	#NIČ!	#NIČ!	#NIČ!	34	#NIČ!
395	2	8564697	6	6	5	6	6	6	6	6	6	6	5	6	7	6	5	5	5	3	4	4	4	3	3	4	1	1		2	1		2	4,76	1,00	2,00	34	#NIČ!
396	2	141578	6	6	7	6	6	7	6	6	7	6	6	7	6	7	7	6	7	3	3	4	4	3	4	4	8	#NIČ!	Uprava	4	#NIČ!	univerz	2	5,41	98,00	7,00	27	1,00
397	2	5013796	7	7	7	6	5	7	5	6	6	5	6	7	6	6	7	3	7	2	5	5	5	4	3	3	3	17		3	5		2	5,06	17,00	1,00	34	#NIČ!
398	2	4644249	4	5	6	6	4	5	6	6	6	6	4	3	6	6	6	3	4	2	3	3	4	3	4	4	2	1		1	4		1	4,06	1,00	2,00	34	#NIČ!
399	2	8179858	5	7	6	7	7	6	6	6	6	7	6	6	7	6	6	1	7	4	5	5	4	4	2	3	4	6		2	1		2	5,00	6,00	3,00	34	#NIČ!
400	2	6966540	6	5	7	7	6	5	5	3	4	7	5	7	5	6	7	3	6	5	3	4	4	5	2	3	2	17		2	2		2	4,53	17,00	1,00	34	#NIČ!
401	2	293129	6	5	7	7	7	7	6	7	6	6	7	7	6	7	7	2	5	3	4	5	4	3	3	3	2	2		2	2		2	5,18	2,00	5,00	34	#NIČ!
402	#NIČ!	566023	#NIČ!	#NIČ!	#NIČ!	#NIČ!	#NIČ!	#NIČ!	#NIČ!	#NIČ!	#NIČ!	#NIČ!	#NIČ!	#NIČ!	#NIČ!	#NIČ!	#NIČ!	#NIČ!	#NIČ!	#NIČ!	#NIČ!	#NIČ!	#NIČ!	#NIČ!	#NIČ!	#NIČ!	#NIČ!	#NIČ!		#NIČ!	#NIČ!		#NIČ!	#NIČ!	#NIČ!	#NIČ!	34	#NIČ!
403	2	1950730	6	6	5	6	5	5	6	5	5	6	5	5	5	6	5	5	5	3	4	4	3	4	3	3	1	2		1	5		1	4,35	2,00	5,00	34	#NIČ!
404	2	8055582	1	7	7	7	7	6	5	5	6	7	7	2	4	7	6	1	7	4	4	1	1	4	3	2	1	1		2	2		2	4,41	1,00	2,00	34	#NIČ!
405	2	4425720	3	6	4	5	3	5	3	2	2	2	3	2	2	3	5	1	2	1	3	1	1	3	2	1	1	1		2	2		2	2,12	1,00	2,00	34	#NIČ!
406	2	7961852	6	6	6	7	7	7	7	7	7	7	7	7	7	7	7	6	6	4	3	4	3	3	3	3	1	1		2	3		2	5,71	1,00	2,00	34	#NIČ!
407	2	6532103	4	5	6	6	6	6	5	5	5	6	6	6	5	5	6	5	5	3	4	4	4	3	3	3	2	#NIČ!		3	3		2	4,41	#NIČ!	#NIČ!	34	#NIČ!
408	2	8774959	3	4	4	4	4	5	3	3	4	4	4	5	3	4	3	3	4	3	4	4	3	2	3	3	2	21		2	2		2	2,76	21,00	1,00	34	#NIČ!
409	2	4278484	6	6	7	6	6	6	6	6	6	6	7	6	6	7	6	7	7	3	3	3	3	3	3	3	2	17		2	2		2	5,29	17,00	1,00	34	#NIČ!
410	2	5067544	5	5	5	5	5	5	5	3	5	5	5	5	5	5	5	3	5	3	3	3	3	3	3	3	3	14		2	4		2	3,76	14,00	1,00	34	#NIČ!
412	2	7308712	3	6	6	6	7	6	7	6	7	7	7	7	7	7	6	1	7	3	3	3	2	3	2	2	5	18		3	2		1	5,06	18,00	3,00	34	#NIČ!
413	2	2502269	7	7	7	7	7	7	7	6	6	6	6	6	6	6	5	5	5	4	5	5	3	3	3	3	6	6		2	2		2	5,24	6,00	3,00	34	#NIČ!
414	2	9036755	6	7	7	7	6	6	6	7	7	7	6	7	6	7	6	5	7	4	5	5	3	4	2	3	1	20		1	5		1	5,47	20,00	1,00	34	#NIČ!
415	2	3257640	2	6	6	5	6	4	5	6	6	6	6	6	6	6	5	1	4	3	3	3	2	2	3	2	2	9		3	4		2	4,06	9,00	1,00	34	#NIČ!
416	2	9177935	7	7	7	6	6	7	5	4	5	7	7	6	7	7	6	6	7	2	4	5	4	3	3	3	4	3		2	3		1	5,29	3,00	5,00	34	#NIČ!
417	2	6116755	4	5	4	4	4	4	4	3	3	3	5	4	4	4	2	4	5	3	4	4	4	4	4	4	5	1		1	5		2	2,88	1,00	2,00	34	#NIČ!
418	2	3049971	7	7	7	7	6	6	7	7	6	7	6	6	7	7	7	5	7	4	3	3	2	3	3	1	1	26		#NIČ!	5		2	5,59	26,00	1,00	34	#NIČ!
419	#NIČ!	6899592	#NIČ!	#NIČ!	#NIČ!	#NIČ!	#NIČ!	#NIČ!	#NIČ!	#NIČ!	#NIČ!	#NIČ!	#NIČ!	#NIČ!	#NIČ!	#NIČ!	#NIČ!	#NIČ!	#NIČ!	#NIČ!	#NIČ!	#NIČ!	#NIČ!	#NIČ!	#NIČ!	#NIČ!	#NIČ!	#NIČ!		#NIČ!	#NIČ!		#NIČ!	#NIČ!	#NIČ!	#NIČ!	34	#NIČ!
420	2	5348044	6	7	7	7	6	6	7	6	6	7	6	6	6	6	6	5	5	5	5	4	3	3	3	3	3	1		1	5		2	5,18	1,00	2,00	34	#NIČ!
422	2	6041447	4	6	7	4	4	7	3	3	3	3	6	4	3	6	4	2	7	4	4	4	4	4	3	2	4	1		2	2		2	3,47	1,00	2,00	34	#NIČ!
423	2	4163103	6	7	7	6	6	6	6	6	6	7	6	5	6	7	6	6	7	4	4	5	5	4	5	5	8	#NIČ!		4	5		1	5,24	#NIČ!	#NIČ!	34	#NIČ!
424	2	2691174	6	7	7	6	6	7	7	6	6	7	6	7	6	7	6	5	6	5	5	5	5	5	5	5	1	1		1	5		2	5,35	1,00	2,00	34	#NIČ!
425	2	966559	6	7	7	7	7	6	6	6	6	7	6	7	7	6	5	6	6	2	3	3	3	4	2	3	4	7		2	4		2	5,35	7,00	4,00	34	#NIČ!
426	2	6759276	5	6	7	6	5	5	6	5	4	6	6	5	5	5	5	4	5	2	5	5	4	4	5	4	6	2		2	4		1	4,29	2,00	5,00	34	#NIČ!
427	2	896702	6	6	6	6	6	5	7	7	5	6	6	5	5	7	5	5	7	1	5	5	5	5	5	5	6	7		2	2		2	4,88	7,00	4,00	34	#NIČ!
428	2	4205681	6	7	7	6	7	7	7	6	7	7	7	6	7	6	5	7	7	3	3	3	3	4	3	2	4	7		2	4		2	5,59	7,00	4,00	34	#NIČ!
429	2	8338298	6	7	7	6	6	6	6	7	6	7	2	6	6	7	6	1	7	4	5	2	5	5	5	5	3	7		2	3		2	4,82	7,00	4,00	34	#NIČ!
430	2	9074451	4	5	2	6	6	6	6	5	7	7	7	7	7	7	7	7	7	3	1	4	2	4	5	5	4	12		3	2		2	5,06	12,00	1,00	34	#NIČ!
431	#NIČ!	357359	#NIČ!	#NIČ!	#NIČ!	#NIČ!	#NIČ!	#NIČ!	#NIČ!	#NIČ!	#NIČ!	#NIČ!	#NIČ!	#NIČ!	#NIČ!	#NIČ!	#NIČ!	#NIČ!	#NIČ!	#NIČ!	#NIČ!	#NIČ!	#NIČ!	#NIČ!	#NIČ!	#NIČ!	#NIČ!	#NIČ!		#NIČ!	#NIČ!		#NIČ!	#NIČ!	#NIČ!	#NIČ!	34	#NIČ!
432	2	4563442	7	7	7	6	6	7	6	6	6	7	6	7	6	7	6	6	6	4	5	5	4	4	3	2	2	1		2	2		2	5,41	1,00	2,00	34	#NIČ!
433	2	1745133	7	7	7	7	6	5	6	6	7	7	5	6	7	6	6	5	6	3	5	5	5	5	5	5	2	7		2	3		2	5,24	7,00	4,00	34	#NIČ!
434	2	5035340	7	7	7	7	6	7	5	5	7	5	7	5	5	7	6	3	7	2	5	5	5	4	5	4	2	7		2	2		2	5,06	7,00	4,00	34	#NIČ!
435	2	9941301	6	7	7	7	7	7	7	6	6	7	7	6	6	7	7	7	7	3	4	4	4	3	4	4	4	15		2	2		2	5,71	15,00	1,00	34	#NIČ!
436	2	4600487	6	7	7	6	6	5	6	7	7	6	6	6	6	7	5	5	7	3	5	4	5	4	4	4	2	7		2	4		2	5,18	7,00	4,00	34	#NIČ!
437	2	3178530	6	7	7	7	7	7	7	7	7	7	7	7	7	7	6	7	7	3	5	5	5	5	5	5	2	7		2	4		2	5,88	7,00	4,00	34	#NIČ!
438	2	2091188	6	7	7	7	7	7	7	6	6	7	7	7	6	7	6	6	7	4	3	3	3	3	4	3	1	7		2	4		2	5,65	7,00	4,00	34	#NIČ!
439	2	920353	5	7	5	6	6	6	7	5	4	6	6	7	7	7	4	5	7	5	3	4	4	3	4	4	1	1		1	5		1	4,88	1,00	2,00	34	#NIČ!

440	2	8328201	5	6	6	5	5	5	6	5	6	6	4	5	7	6	5	4	7	3	4	4	4	4	4	4	3	12		3		5		1	4,47	12,00	1,00	34	#NIČ!
441	2	8879945	5	6	7	7	7	6	5	6	6	7	5	4	5	6	5	4	5	5	5	5	4	5	5	5	2	7		2		4		2	4,65	7,00	4,00	34	#NIČ!
442	2	9415122	7	7	7	7	7	6	7	7	7	7	7	7	7	7	7	6	6	6	4	4	5	5	5	4	2	7		2		4		2	5,76	7,00	4,00	34	#NIČ!
443	2	435778	5	7	7	5	4	3	3	4	1	6	7	4	2	6	2	2	4	2	5	5	5	3	2	2	6	5		2		2		2	3,24	5,00	3,00	34	#NIČ!
444	2	3933521	4	4	7	5	4	4	4	3	4	5	4	4	5	5	4	4	4	3	3	3	3	3	3	3	4	4		2		4		2	3,35	4,00	6,00	34	#NIČ!
445	2	8360272	7	7	7	7	7	7	7	7	7	7	7	7	7	7	6	6	7	1	4	2	4	1	4	4	1	14		2		4		2	5,88	14,00	1,00	34	#NIČ!
446	2	800	7	7	7	6	6	6	6	4	6	7	4	4	6	5	6	2	7	4	5	5	4	4	4	4	3	2		1		5		2	4,65	2,00	5,00	34	#NIČ!
447	2	4923182	4	7	5	5	6	4	5	4	5	6	5	4	5	6	4	3	5	4	4	4	4	4	4	4	4	17		1		5		1	3,88	17,00	1,00	34	#NIČ!
448	2	4613510	7	7	7	7	7	6	6	6	6	6	6	6	6	6	6	6	6	4	4	4	4	4	4	4	3	17		2		3		2	5,29	17,00	1,00	34	#NIČ!
449	2	8298006	4	7	7	7	7	4	7	6	6	7	5	7	7	7	7	4	7	3	5	5	4	5	4	4	6	18		3		2		2	5,24	18,00	3,00	34	#NIČ!
450	#NIČ!	7649501	#NIČ!	#NIČ!	#NIČ!	#NIČ!	#NIČ!	#NIČ!	#NIČ!	#NIČ!	#NIČ!	#NIČ!	#NIČ!	#NIČ!	#NIČ!	#NIČ!	#NIČ!	#NIČ!	#NIČ!	#NIČ!	#NIČ!	#NIČ!	#NIČ!	#NIČ!	#NIČ!	#NIČ!	#NIČ!	#NIČ!		#NIČ!		#NIČ!		#NIČ!	#NIČ!	#NIČ!	#NIČ!	34	#NIČ!
451	2	3353485	4	7	7	6	6	7	5	4	6	6	5	6	6	6	4	5	5	3	3	3	3	3	3	3	2	16		2		2		2	4,59	16,00	1,00	34	#NIČ!
452	0	3818921	#NIČ!	#NIČ!	#NIČ!	#NIČ!	#NIČ!	#NIČ!	#NIČ!	#NIČ!	#NIČ!	#NIČ!	#NIČ!	#NIČ!	#NIČ!	#NIČ!	#NIČ!	#NIČ!	#NIČ!	#NIČ!	#NIČ!	#NIČ!	#NIČ!	#NIČ!	#NIČ!	#NIČ!	#NIČ!	#NIČ!		#NIČ!		#NIČ!		#NIČ!	#NIČ!	#NIČ!	#NIČ!	34	#NIČ!
453	#NIČ!	9034154	#NIČ!	#NIČ!	#NIČ!	#NIČ!	#NIČ!	#NIČ!	#NIČ!	#NIČ!	#NIČ!	#NIČ!	#NIČ!	#NIČ!	#NIČ!	#NIČ!	#NIČ!	#NIČ!	#NIČ!	#NIČ!	#NIČ!	#NIČ!	#NIČ!	#NIČ!	#NIČ!	#NIČ!	#NIČ!	#NIČ!		#NIČ!		#NIČ!		#NIČ!	#NIČ!	#NIČ!	#NIČ!	34	#NIČ!
454	2	3714006	6	7	7	6	6	6	6	6	6	7	6	6	7	7	7	4	7	4	4	4	4	3	4	4	2	16		1		5		2	5,29	16,00	1,00	34	#NIČ!
455	2	1467566	6	6	6	6	6	6	6	6	6	6	6	4	6	6	6	6	6	3	3	3	3	3	3	3	1	1		2		2		2	4,88	1,00	2,00	34	#NIČ!
456	2	6195815	5	6	6	6	6	6	6	6	6	6	6	6	6	6	6	4	6	3	4	3	2	3	3	2	7	#NIČ!		2		4		1	4,82	#NIČ!	#NIČ!	34	#NIČ!
457	2	6576375	2	4	4	4	3	5	4	3	3	4	3	4	4	4	2	2	2	1	4	5	2	2	4	2	7	#NIČ!		1		5		2	2,35	#NIČ!	#NIČ!	34	#NIČ!
458	0	4294431	#NIČ!	#NIČ!	#NIČ!	#NIČ!	#NIČ!	#NIČ!	#NIČ!	#NIČ!	#NIČ!	#NIČ!	#NIČ!	#NIČ!	#NIČ!	#NIČ!	#NIČ!	#NIČ!	#NIČ!	#NIČ!	#NIČ!	#NIČ!	#NIČ!	#NIČ!	#NIČ!	#NIČ!	#NIČ!	#NIČ!		#NIČ!		#NIČ!		#NIČ!	#NIČ!	#NIČ!	#NIČ!	34	#NIČ!
459	2	1743032	6	6	6	6	5	4	4	5	5	5	5	5	5	7	6	5	6	3	4	4	4	3	3	3	5	#NIČ!		4		2		2	4,35	#NIČ!	1,00	34	#NIČ!
461	2	5831866	7	7	7	7	7	7	7	7	7	7	7	7	7	7	7	7	7	5	5	5	5	5	5	5	3	3		1		5		1	6,00	3,00	5,00	34	#NIČ!
462	2	3930233	7	7	7	7	7	7	7	7	7	7	7	7	7	7	7	7	7	5	5	5	5	5	5	5	6	5		3		3		2	6,00	5,00	3,00	34	#NIČ!
463	2	2155566	4	5	7	7	7	7	7	7	7	7	7	7	7	7	6	7	7	5	4	5	5	5	5	5	3	17		2		2		2	5,65	17,00	1,00	34	#NIČ!
464	2	8987135	5	6	7	6	6	7	6	6	7	5	6	7	6	6	6	6	6	3	4	4	4	4	4	4	2	#NIČ!	radiolog	1		5		1	5,12	98,00	7,00	15	1,00
465	2	8468975	4	6	7	4	4	4	4	3	4	4	3	7	4	4	7	1	6	2	5	5	1	3	4	2	1	6		2		2		2	3,47	6,00	3,00	34	#NIČ!
466	2	5383470	6	7	7	6	5	5	5	5	4	6	5	5	5	7	6	4	7	3	4	4	3	3	3	3	2	9		2		2		2	4,59	9,00	1,00	34	#NIČ!
467	2	1510425	7	7	7	6	7	6	6	6	7	7	7	7	7	6	6	6	6	4	4	5	5	5	5	5	3	7		2		4		2	5,53	7,00	4,00	34	#NIČ!
468	2	1401715	6	7	7	7	6	7	6	6	6	7	6	7	6	7	7	6	7	4	5	5	4	4	5	5	2	9		3		4		1	5,53	9,00	1,00	34	#NIČ!
469	2	2477302	6	5	7	5	5	6	5	5	4	6	6	3	6	6	4	4	6	5	3	3	3	4	4	4	3	2		2		2		2	4,24	2,00	5,00	34	#NIČ!
470	#NIČ!	8181364	#NIČ!	#NIČ!	#NIČ!	#NIČ!	#NIČ!	#NIČ!	#NIČ!	#NIČ!	#NIČ!	#NIČ!	#NIČ!	#NIČ!	#NIČ!	#NIČ!	#NIČ!	#NIČ!	#NIČ!	#NIČ!	#NIČ!	#NIČ!	#NIČ!	#NIČ!	#NIČ!	#NIČ!	#NIČ!	#NIČ!		#NIČ!		#NIČ!		#NIČ!	#NIČ!	#NIČ!	#NIČ!	34	#NIČ!
471	2	3474914	6	7	7	7	7	7	7	6	7	7	7	7	7	7	6	6	7	3	4	5	5	5	5	4	1	#NIČ!	INFORM	#NIČ!	JO330	#NIČ!	3.letnik	2	5,76	98,00	7,00	12	1,00
472	2	2830479	7	7	7	7	6	7	6	7	7	7	7	7	7	6	6	6	6	4	5	4	4	4	5	5	4	9		3		4		2	5,65	9,00	1,00	34	#NIČ!
473	2	3727654	4	6	7	5	6	7	4	4	7	7	7	7	7	6	3	7	7	4	4	5	2	2	4	2	8	#NIČ!	adminis	4		3		1	4,94	98,00	7,00	1	1,00
474	2	146834	4	4	5	5	5	5	5	5	4	5	5	5	4	5	4	4	4	3	3	3	3	3	3	3	4	7		2		4		2	3,59	7,00	4,00	34	#NIČ!
475	2	9551206	4	5	7	4	2	6	1	2	1	1	7	5	1	5	5	2	6	2	5	4	2	2	2	1	2	9		2		4		2	2,76	9,00	1,00	34	#NIČ!
476	2	7315529	6	7	7	6	6	7	6	6	5	5	6	4	4	7	6	4	7	2	3	3	3	2	2	2	4	8		3		4		2	4,82	8,00	1,00	34	#NIČ!
477	2	7924027	7	7	7	6	6	5	5	5	6	6	5	6	6	6	5	6	6	3	4	3	3	4	3	3	5	1		2		2		2	4,88	1,00	2,00	34	#NIČ!
478	2	7673550	6	7	7	6	5	7	6	5	4	5	5	5	5	7	6	4	7	4	4	4	3	3	4	3	2	9		3		4		2	4,71	9,00	1,00	34	#NIČ!
479	2	4595667	5	7	7	6	6	6	5	5	5	7	6	6	6	5	5	5	6	2	4	4	3	2	2	2	3	6		2		2		2	4,76	6,00	3,00	34	#NIČ!
480	2	9957685	6	7	7	7	6	7	6	6	7	7	6	6	6	6	7	6	6	3	5	5	3	3	3	3	3	6		2		2		2	5,41	6,00	3,00	34	#NIČ!
481	2	6001425	4	5	5	4	4	4	3	3	2	3	3	2	2	3	3	1	3	1	2	3	2	2	2	2	3	6		1		4		2	2,18	6,00	3,00	34	#NIČ!
482	2	134069	6	6	7	6	5	4	4	6	5	5	6	6	6	5	5	6	6	3	4	5	4	4	3	3	7	#NIČ!	SNMP	4		2		2	4,53	98,00	7,00	21	1,00
483	#NIČ!	2666070	#NIČ!	#NIČ!	#NIČ!	#NIČ!	#NIČ!	#NIČ!	#NIČ!	#NIČ!	#NIČ!	#NIČ!	#NIČ!	#NIČ!	#NIČ!	#NIČ!	#NIČ!	#NIČ!	#NIČ!	#NIČ!	#NIČ!	#NIČ!	#NIČ!	#NIČ!	#NIČ!	#NIČ!	#NIČ!	#NIČ!		#NIČ!		#NIČ!		#NIČ!	#NIČ!	#NIČ!	#NIČ!	34	#NIČ!
484	2	2928142	4	6	6	5	4	6	4	5	6	6	6	5	5	6	5	4	7	4	2	3	2	2	4	2	6	#NIČ!	ambular	1		5		2	4,29	98,00	7,00	6	1,00
485	2	6642504	3	6	5	4	4	5	4	4	5	5	4	3	4	4	3	2	6	3	3	3	3	3	3	3	4	1		1		5		2	3,18	1,00	2,00	34	#NIČ!
486	2	4428091	6	7	7	7	6	7	6	6	6	6	6	7	6	7	7	6	7	4	4	4	4	4	4	4	5	1		2		4		1	5,47	1,00	2,00	34	#NIČ!
487	2	2212945	5	6	7	6	6	6	5	5	5	5	6	6	6	6	6	5	6	2	3	3	4	4	5	4	6	7		2		4		2	4,71	7,00	4,00	34	#NIČ!
488	2	7780454	3	6	6	4	4	3	3	3	1	4	2	3	4	4	4	4	5	2	3	3	3	3	3	3	7	#NIČ!	snmp	2		2		2	2,71	98,00	7,00	19	1,00
489	#NIČ!	2263433	#NIČ!	#NIČ!	#NIČ!	#NIČ!	#NIČ!	#NIČ!	#NIČ!	#NIČ!	#NIČ!	#NIČ!	#NIČ!	#NIČ!	#NIČ!	#NIČ!	#NIČ!	#NIČ!	#NIČ!	#NIČ!	#NIČ!	#NIČ!	#NIČ!	#NIČ!	#NIČ!	#NIČ!	#NIČ!	#NIČ!		#NIČ!		#NIČ!		#NIČ!	#NIČ!	#NIČ!	#NIČ!	34	#NIČ!
490	2	7975803	5	6	6	5	5	5	5	5	6	5	5	5	5	5	5	5	5	3	3	3	3	3	3	3	4	1		2		3		2	4,18	1,00	2,00	34	#NIČ!
491	#NIČ!	3088717	#NIČ!	#NIČ!	#NIČ!	#NIČ!	#NIČ!	#NIČ!	#NIČ!	#NIČ!	#NIČ!	#NIČ!	#NIČ!	#NIČ!	#NIČ!	#NIČ!	#NIČ!	#NIČ!	#NIČ!	#NIČ!	#NIČ!	#NIČ!	#NIČ!	#NIČ!	#NIČ!	#NIČ!	#NIČ!	#NIČ!		#NIČ!		#NIČ!		#NIČ!	#NIČ!	#NIČ!	#NIČ!	34	#NIČ!
492	2	1516177	7	6	5	6	6	6	6	7	7	7	5	5	4	6	6	1	7	3	5	4	3	3	3	3	5	12		3		5		2	4,71	12,00	1,00	34	#NIČ!
493	2	8314734	6	6	6	6	5	6	2	4	5	6	5	5	4	6	5	2	5	3	4	4	2	2	2	1	8	#NIČ!		4		4		2	3,94	#NIČ!	#NIČ!	34	#NIČ!
494	2	7025138	4	7	6	5	3	2	3	3	3	3	3	5	4	4	6	4	6	3	3	3	3	3	3	3	8	#NIČ!		4		4		1	3,18	#NIČ!	1,00	34	#NIČ!

495	2	181487	6	7	7	6	6	4	6	6	6	6	5	6	6	7	6	5	7	4	5	5	5	5	4	3	3	8		3	4	2	5,00	8,00	1,00	34	#NIČ!
497	2	9832024	7	7	7	7	7	7	7	7	7	7	7	7	7	7	7	7	7	5	5	5	5	5	5	5	1	11		3	4	2	6,00	11,00	1,00	34	#NIČ!
498	2	8615658	3	4	6	4	4	5	5	4	4	5	5	4	4	5	3	4	4	2	3	3	2	3	3	3	1	2		1	5	2	3,29	2,00	5,00	34	#NIČ!
499	2	3582217	5	7	7	6	7	7	6	7	6	7	7	6	6	6	6	6	6	3	3	3	2	3	3	3	3	2		2	4	2	5,35	2,00	5,00	34	#NIČ!
500	2	2064111	4	6	7	5	6	6	5	4	4	6	5	6	6	5	5	4	7	3	5	5	4	4	4	4	2	15		2	1	2	4,35	15,00	1,00	34	#NIČ!
501	2	9573904	2	3	3	7	3	2	3	3	2	3	2	2	2	1	3	1	2	2	2	2	2	2	1	2	1	4		2	2	2	1,59	4,00	6,00	34	#NIČ!
502	2	1677189	5	7	7	5	7	6	6	6	6	5	6	6	7	7	5	6	7	4	3	5	5	4	3	4	1	5		1	3	2	5,12	5,00	3,00	34	#NIČ!
503	2	9664231	4	5	7	6	5	7	5	4	4	7	6	7	6	5	4	6	7	2	4	5	2	3	1	3	4	26		2	4	2	4,59	26,00	1,00	34	#NIČ!
504	2	3289589	5	6	6	5	5	4	5	3	5	7	5	2	6	5	2	5	5	3	3	3	3	2	2	2	1	7		2	5	2	3,76	7,00	4,00	34	#NIČ!
505	2	7455253	6	7	7	5	5	6	4	4	4	4	4	6	5	4	3	4	5	2	3	3	3	3	3	3	4	17		2	2	2	3,88	17,00	1,00	34	#NIČ!
506	2	7407499	6	6	7	6	5	5	5	6	6	5	6	4	4	5	5	3	4	3	5	5	3	3	4	3	4	17		2	4	2	4,18	17,00	1,00	34	#NIČ!
507	#NIČ!	4671736	#NIČ!	#NIČ!	#NIČ!	#NIČ!	#NIČ!	#NIČ!	#NIČ!	#NIČ!	#NIČ!	#NIČ!	#NIČ!	#NIČ!	#NIČ!	#NIČ!	#NIČ!	#NIČ!	#NIČ!	#NIČ!	#NIČ!	#NIČ!	#NIČ!	#NIČ!	#NIČ!	#NIČ!	#NIČ!	#NIČ!		#NIČ!	#NIČ!	#NIČ!	#NIČ!	#NIČ!	#NIČ!	34	#NIČ!
508	2	1136184	7	7	7	7	7	7	7	7	7	7	7	6	7	7	6	6	7	3	5	5	4	5	5	5	3	4		2	2	2	5,82	4,00	6,00	34	#NIČ!
509	2	1665713	7	7	7	7	7	6	7	7	7	7	7	7	7	7	5	5	5	2	5	5	4	4	2	3	4	7		2	4	2	5,59	7,00	4,00	34	#NIČ!
510	2	4920011	6	5	6	6	5	6	6	4	6	6	6	5	6	6	4	5	6	2	4	3	2	3	3	2	6	11		1	5	1	4,53	11,00	1,00	34	#NIČ!
511	#NIČ!	9602918	#NIČ!	#NIČ!	#NIČ!	#NIČ!	#NIČ!	#NIČ!	#NIČ!	#NIČ!	#NIČ!	#NIČ!	#NIČ!	#NIČ!	#NIČ!	#NIČ!	#NIČ!	#NIČ!	#NIČ!	#NIČ!	#NIČ!	#NIČ!	#NIČ!	#NIČ!	#NIČ!	#NIČ!	#NIČ!	#NIČ!		#NIČ!	#NIČ!	#NIČ!	#NIČ!	#NIČ!	#NIČ!	34	#NIČ!
512	2	3254556	6	6	6	6	6	6	6	5	6	6	5	5	5	5	6	5	5	4	4	4	4	3	4	4	6	1		2	4	1	4,59	1,00	2,00	34	#NIČ!
513	2	7464026	4	7	6	6	7	6	7	6	7	7	6	6	7	7	6	7	7	3	5	5	4	4	5	5	3	5		3	2	1	5,41	5,00	3,00	34	#NIČ!
514	2	7556464	6	7	6	6	6	5	5	6	6	6	6	6	6	6	4	5	6	3	4	4	4	4	3	4	2	6		1	4	1	4,76	6,00	3,00	34	#NIČ!
516	#NIČ!	5390243	#NIČ!	#NIČ!	#NIČ!	#NIČ!	#NIČ!	#NIČ!	#NIČ!	#NIČ!	#NIČ!	#NIČ!	#NIČ!	#NIČ!	#NIČ!	#NIČ!	#NIČ!	#NIČ!	#NIČ!	#NIČ!	#NIČ!	#NIČ!	#NIČ!	#NIČ!	#NIČ!	#NIČ!	#NIČ!	#NIČ!		#NIČ!	#NIČ!	#NIČ!	#NIČ!	#NIČ!	#NIČ!	34	#NIČ!
517	2	4281822	6	7	5	6	5	4	4	4	5	5	5	5	5	5	4	4	6	2	3	3	2	2	2	2	3	#NIČ!		#NIČ!	#NIČ!	2	4,00	#NIČ!	#NIČ!	34	#NIČ!
518	2	5238383	5	6	6	5	6	4	5	6	5	7	4	4	6	5	4	4	4	3	4	5	4	4	5	4	4	7		2	3	1	4,06	7,00	4,00	34	#NIČ!
519	2	3346446	7	7	7	6	7	7	7	7	7	7	7	7	7	7	7	7	7	5	5	4	5	5	5	5	3	3		2	2	2	5,94	3,00	5,00	34	#NIČ!
520	2	1017084	6	6	7	6	5	5	5	5	5	6	5	5	6	5	6	5	6	4	3	5	4	3	3	3	2	17		2	2	2	4,53	17,00	1,00	34	#NIČ!
521	2	5046080	6	7	7	6	6	5	6	6	6	7	5	7	6	7	6	3	7	1	5	5	4	3	4	4	1	7		2	4	2	5,06	7,00	4,00	34	#NIČ!
522	2	2179150	7	7	7	7	7	7	7	6	7	7	7	7	7	7	6	7	7	4	4	4	4	3	4	5	1	1		2	4	2	5,88	1,00	2,00	34	#NIČ!
523	2	5757508	6	6	6	6	6	6	6	6	6	7	6	6	6	7	6	5	6	3	4	5	4	4	4	4	2	9		3	4	2	5,06	9,00	1,00	34	#NIČ!
524	2	2250093	5	7	7	6	6	6	7	4	7	7	7	6	6	5	5	3	7	2	4	5	4	3	5	5	3	1		2	5	2	4,94	1,00	2,00	34	#NIČ!
525	#NIČ!	3977939	#NIČ!	#NIČ!	#NIČ!	#NIČ!	#NIČ!	#NIČ!	#NIČ!	#NIČ!	#NIČ!	#NIČ!	#NIČ!	#NIČ!	#NIČ!	#NIČ!	#NIČ!	#NIČ!	#NIČ!	#NIČ!	#NIČ!	#NIČ!	#NIČ!	#NIČ!	#NIČ!	#NIČ!	#NIČ!	#NIČ!		#NIČ!	#NIČ!	#NIČ!	#NIČ!	#NIČ!	#NIČ!	34	#NIČ!
526	2	3139418	6	7	6	6	6	5	5	5	5	6	5	5	6	6	5	5	5	4	4	5	4	4	3	3	2	6		2	2	2	4,53	6,00	3,00	34	#NIČ!
527	2	3763271	7	7	7	7	7	7	7	7	7	7	7	7	7	7	7	7	7	5	5	5	5	5	5	5	6	1		2	1	2	6,00	1,00	2,00	34	#NIČ!
528	#NIČ!	9398104	#NIČ!	#NIČ!	#NIČ!	#NIČ!	#NIČ!	#NIČ!	#NIČ!	#NIČ!	#NIČ!	#NIČ!	#NIČ!	#NIČ!	#NIČ!	#NIČ!	#NIČ!	#NIČ!	#NIČ!	#NIČ!	#NIČ!	#NIČ!	#NIČ!	#NIČ!	#NIČ!	#NIČ!	#NIČ!	#NIČ!		#NIČ!	#NIČ!	#NIČ!	#NIČ!	#NIČ!	#NIČ!	34	#NIČ!
529	2	5700707	5	6	6	5	5	4	6	5	4	5	5	4	4	4	3	3	4	2	5	3	3	3	3	2	5	8		3	4	1	3,59	8,00	1,00	34	#NIČ!
530	#NIČ!	309221	#NIČ!	#NIČ!	#NIČ!	#NIČ!	#NIČ!	#NIČ!	#NIČ!	#NIČ!	#NIČ!	#NIČ!	#NIČ!	#NIČ!	#NIČ!	#NIČ!	#NIČ!	#NIČ!	#NIČ!	#NIČ!	#NIČ!	#NIČ!	#NIČ!	#NIČ!	#NIČ!	#NIČ!	#NIČ!	#NIČ!		#NIČ!	#NIČ!	#NIČ!	#NIČ!	#NIČ!	#NIČ!	34	#NIČ!
531	2	4443985	4	6	7	5	4	4	3	4	3	3	3	4	2	3	6	2	6	4	3	3	3	3	3	2	1	26		2	3	2	3,06	26,00	1,00	34	#NIČ!
532	2	1292261	5	7	7	7	6	6	6	6	7	7	7	6	6	7	6	6	7	5	5	5	5	5	4	4	3	23		2	2	2	5,41	98,00	1,00	34	#NIČ!
533	2	3129352	5	6	7	6	4	7	5	5	3	6	7	3	4	6	6	4	6	2	4	3	3	3	4	2	3	1		1	5	2	4,29	1,00	2,00	34	#NIČ!
534	2	1769979	7	7	7	7	7	7	7	7	6	7	6	7	6	7	6	6	6	5	4	5	4	4	4	4	3	4		1	5	1	5,65	4,00	6,00	34	#NIČ!
535	2	9461836	4	7	7	5	3	7	4	4	1	7	6	3	4	6	2	4	7	2	3	3	2	3	3	2	6	1		1	5	2	3,76	1,00	2,00	34	#NIČ!
536	2	1999244	3	7	7	5	5	5	5	4	4	6	4	5	5	5	4	4	5	3	3	3	3	3	1	2	3	5		1	4	2	3,88	5,00	3,00	34	#NIČ!
537	2	1610715	7	7	7	7	5	7	5	5	5	7	7	7	7	7	5	5	7	3	3	3	3	2	3	3	2	17		1	5	2	5,29	17,00	1,00	34	#NIČ!
538	2	2055841	7	7	7	7	7	7	7	7	6	6	7	6	6	7	4	1	4	1	5	5	3	2	1	2	6	2		1	5	2	5,06	2,00	5,00	34	#NIČ!
539	2	5447060	6	6	7	6	6	6	5	5	6	6	6	6	6	6	5	6	6	4	4	4	4	4	5	4	3	2		2	4	1	4,88	2,00	5,00	34	#NIČ!
540	2	1067779	4	7	6	6	7	6	7	7	6	6	2	5	6	7	7	3	5	2	4	4	3	3	5	3	3	1		2	3	2	4,71	1,00	2,00	34	#NIČ!
541	2	8997713	7	7	7	7	7	7	7	7	7	7	7	7	7	7	7	7	7	5	5	5	5	5	5	5	3	5		2	2	2	6,00	5,00	3,00	34	#NIČ!
542	2	1785228	6	7	7	7	7	6	6	7	6	7	7	6	7	7	6	6	7	3	4	4	3	3	4	4	7	#NIČ!	Ambulai	2	2	2	5,59	98,00	7,00	5	1,00
543	2	1933003	6	7	6	6	7	6	5	4	6	5	5	6	6	6	6	4	5	2	3	4	3	3	2	3	4	12		5	6	1	4,65	12,00	1,00	34	#NIČ!
544	0	4309331	#NIČ!	#NIČ!	#NIČ!	#NIČ!	#NIČ!	#NIČ!	#NIČ!	#NIČ!	#NIČ!	#NIČ!	#NIČ!	#NIČ!	#NIČ!	#NIČ!	#NIČ!	#NIČ!	#NIČ!	#NIČ!	#NIČ!	#NIČ!	#NIČ!	#NIČ!	#NIČ!	#NIČ!	#NIČ!	#NIČ!		#NIČ!	#NIČ!	#NIČ!	#NIČ!	#NIČ!	#NIČ!	34	#NIČ!
545	2	5747645	4	6	6	5	5	7	5	5	4	5	6	6	6	5	5	3	5	4	3	5	5	5	5	5	1	1		1	5	1	4,18	1,00	2,00	34	#NIČ!
546	2	5810234	7	7	7	7	7	7	7	7	7	7	6	7	7	7	6	6	7	5	4	5	5	5	4	4	3	12		3	1	2	5,82	12,00	1,00	34	#NIČ!
547	2	1808234	3	4	7	3	3	6	3	3	4	4	7	3	3	5	3	1	2	3	3	3	3	3	3	3	2	19		2	2	2	2,76	98,00	1,00	34	#NIČ!
548	0	1610470	#NIČ!	#NIČ!	#NIČ!	#NIČ!	#NIČ!	#NIČ!	#NIČ!	#NIČ!	#NIČ!	#NIČ!	#NIČ!	#NIČ!	#NIČ!	#NIČ!	#NIČ!	#NIČ!	#NIČ!	#NIČ!	#NIČ!	#NIČ!	#NIČ!	#NIČ!	#NIČ!	#NIČ!	#NIČ!	#NIČ!		#NIČ!	#NIČ!	#NIČ!	#NIČ!	#NIČ!	#NIČ!	34	#NIČ!
549	2	2627649	7	7	7	6	5	6	6	6	6	4	4	7	7	7	5	7	7	2	5	5	5	3	3	3	8	#NIČ!	uprava	3	2	2	5,12	98,00	7,00	26	1,00
550	2	8306833	6	7	7	5	7	4	6	6	7	7	7	3	6	7	6	3	7	4	4	4	2	2	2	2	3	1		1	5	2	4,94	1,00	2,00	34	#NIČ!

551	2	3651217	6	7	7	7	7	6	7	6	6	7	6	6	6	7	7	4	7	5	4	5	5	5	5	5	6	14		2	4	2	5,41	14,00	1,00	34	#NIČ!
552	#NIČ!	3335589	#NIČ!	#NIČ!	#NIČ!	#NIČ!	#NIČ!	#NIČ!	#NIČ!	#NIČ!	#NIČ!	#NIČ!	#NIČ!	#NIČ!	#NIČ!	#NIČ!	#NIČ!	#NIČ!	#NIČ!	#NIČ!	#NIČ!	#NIČ!	#NIČ!	#NIČ!	#NIČ!	#NIČ!	#NIČ!	#NIČ!		#NIČ!	#NIČ!	#NIČ!	#NIČ!	#NIČ!	#NIČ!	34	#NIČ!
553	#NIČ!	5724294	#NIČ!	#NIČ!	#NIČ!	#NIČ!	#NIČ!	#NIČ!	#NIČ!	#NIČ!	#NIČ!	#NIČ!	#NIČ!	#NIČ!	#NIČ!	#NIČ!	#NIČ!	#NIČ!	#NIČ!	#NIČ!	#NIČ!	#NIČ!	#NIČ!	#NIČ!	#NIČ!	#NIČ!	#NIČ!	#NIČ!		#NIČ!	#NIČ!	#NIČ!	#NIČ!	#NIČ!	#NIČ!	34	#NIČ!
554	2	8614702	7	7	7	7	7	7	5	7	5	7	5	6	6	7	7	3	7	3	5	5	5	5	5	5	6	5		2	2	2	5,29	5,00	3,00	34	#NIČ!
555	#NIČ!	5900629	#NIČ!	#NIČ!	#NIČ!	#NIČ!	#NIČ!	#NIČ!	#NIČ!	#NIČ!	#NIČ!	#NIČ!	#NIČ!	#NIČ!	#NIČ!	#NIČ!	#NIČ!	#NIČ!	#NIČ!	#NIČ!	#NIČ!	#NIČ!	#NIČ!	#NIČ!	#NIČ!	#NIČ!	#NIČ!	#NIČ!		#NIČ!	#NIČ!	#NIČ!	#NIČ!	#NIČ!	#NIČ!	34	#NIČ!
556	2	3659040	6	7	7	7	7	7	7	7	7	7	7	7	5	7	6	3	7	3	5	5	5	5	3	3	4	15		3	6	2	5,53	15,00	1,00	34	#NIČ!
557	2	593313	5	6	7	6	6	5	6	6	6	6	6	5	6	6	5	4	5	2	4	4	4	3	5	5	2	4		2	3	2	4,65	4,00	6,00	34	#NIČ!
558	2	1989445	4	5	6	5	6	6	4	4	5	6	6	5	4	4	4	4	5	3	4	4	4	4	3	3	2	3		1	5	2	3,88	3,00	5,00	34	#NIČ!
559	2	8167284	5	7	7	6	6	5	7	7	5	7	4	6	7	7	4	4	7	3	5	5	5	5	4	3	4	8		3	5	1	4,94	8,00	1,00	34	#NIČ!
560	2	4868089	6	7	6	6	5	5	7	5	6	7	3	4	4	6	5	1	7	2	3	3	2	2	2	2	6	9		3	3	2	4,29	9,00	1,00	34	#NIČ!
561	2	9838590	4	5	5	4	4	3	3	3	3	5	3	2	3	6	3	3	6	2	3	1	1	3	2	2	3	1		2	1	2	2,82	1,00	2,00	34	#NIČ!
562	2	4588686	6	7	7	7	7	6	6	6	7	7	7	7	7	7	6	5	6	4	4	3	3	3	1	1	4	5		2	2	2	5,53	5,00	3,00	34	#NIČ!
563	2	3427660	2	7	4	1	1	2	1	1	1	4	1	6	2	6	2	2	4	2	4	4	4	2	1	1	1	5		2	2	2	1,76	5,00	3,00	34	#NIČ!
564	#NIČ!	3372243	#NIČ!	#NIČ!	#NIČ!	#NIČ!	#NIČ!	#NIČ!	#NIČ!	#NIČ!	#NIČ!	#NIČ!	#NIČ!	#NIČ!	#NIČ!	#NIČ!	#NIČ!	#NIČ!	#NIČ!	#NIČ!	#NIČ!	#NIČ!	#NIČ!	#NIČ!	#NIČ!	#NIČ!	#NIČ!	#NIČ!		#NIČ!	#NIČ!	#NIČ!	#NIČ!	#NIČ!	#NIČ!	34	#NIČ!
565	2	6578233	6	6	7	6	5	7	5	6	5	5	5	6	5	6	5	6	7	4	5	5	3	3	3	3	2	17		1	5	2	4,76	17,00	1,00	34	#NIČ!
566	2	2774439	4	4	4	4	5	6	6	6	6	6	6	6	6	6	5	5	5	3	3	3	3	3	3	3	2	1		2	4	2	4,29	1,00	2,00	34	#NIČ!
567	2	4137493	6	6	7	6	6	7	6	6	6	6	6	6	6	6	5	6	6	1	4	4	1	3	1	1	6	15		3	5	1	5,06	15,00	1,00	34	#NIČ!
568	#NIČ!	2364152	#NIČ!	#NIČ!	#NIČ!	#NIČ!	#NIČ!	#NIČ!	#NIČ!	#NIČ!	#NIČ!	#NIČ!	#NIČ!	#NIČ!	#NIČ!	#NIČ!	#NIČ!	#NIČ!	#NIČ!	#NIČ!	#NIČ!	#NIČ!	#NIČ!	#NIČ!	#NIČ!	#NIČ!	#NIČ!	#NIČ!		#NIČ!	#NIČ!	#NIČ!	#NIČ!	#NIČ!	#NIČ!	34	#NIČ!
569	2	9408278	5	6	7	6	6	7	5	6	6	6	6	5	5	7	6	5	6	2	5	4	3	2	2	1	3	1		2	2	2	4,88	1,00	2,00	34	#NIČ!
570	#NIČ!	9948935	#NIČ!	#NIČ!	#NIČ!	#NIČ!	#NIČ!	#NIČ!	#NIČ!	#NIČ!	#NIČ!	#NIČ!	#NIČ!	#NIČ!	#NIČ!	#NIČ!	#NIČ!	#NIČ!	#NIČ!	#NIČ!	#NIČ!	#NIČ!	#NIČ!	#NIČ!	#NIČ!	#NIČ!	#NIČ!	#NIČ!		#NIČ!	#NIČ!	#NIČ!	#NIČ!	#NIČ!	#NIČ!	34	#NIČ!
571	2	1519842	4	6	6	6	6	6	6	5	5	6	6	6	6	6	6	6	6	3	3	3	3	3	3	3	6	6		1	5	2	4,76	6,00	3,00	34	#NIČ!
572	#NIČ!	7752404	#NIČ!	#NIČ!	#NIČ!	#NIČ!	#NIČ!	#NIČ!	#NIČ!	#NIČ!	#NIČ!	#NIČ!	#NIČ!	#NIČ!	#NIČ!	#NIČ!	#NIČ!	#NIČ!	#NIČ!	#NIČ!	#NIČ!	#NIČ!	#NIČ!	#NIČ!	#NIČ!	#NIČ!	#NIČ!	#NIČ!		#NIČ!	#NIČ!	#NIČ!	#NIČ!	#NIČ!	#NIČ!	34	#NIČ!
573	2	4202493	6	7	6	7	7	6	7	6	5	7	6	7	7	7	6	6	7	4	4	5	5	4	5	4	3	7		2	2	2	5,47	7,00	4,00	34	#NIČ!
574	2	7755255	7	7	7	7	7	7	7	7	7	7	7	7	7	7	7	6	4	4	5	5	5	5	4	4	2	8		3	4	2	5,76	8,00	1,00	34	#NIČ!
575	2	6168796	6	7	7	7	6	6	5	5	6	6	6	6	5	6	5	4	6	5	5	5	5	3	4	4	3	15		2	3	2	4,82	15,00	1,00	34	#NIČ!
576	2	7578215	5	5	5	5	4	6	3	5	4	4	5	5	4	7	4	3	7	3	4	4	4	4	4	3	4	1		2	2	2	3,76	1,00	2,00	34	#NIČ!
577	2	9384689	6	7	7	7	7	4	6	5	5	7	7	5	7	7	7	4	6	2	3	2	2	3	4	2	7	#NIČ!	Urgentn	1	6	2	5,12	98,00	7,00	30	1,00
578	2	4188801	5	6	7	5	4	7	4	3	4	4	4	6	3	3	4	3	7	3	4	4	3	4	2	1	5	1		2	2	2	3,65	1,00	2,00	34	#NIČ!
579	2	2789938	6	7	6	6	7	5	6	6	6	6	6	6	6	6	5	4	6	5	4	5	5	5	5	5	1	12		3	5	2	4,88	12,00	1,00	34	#NIČ!
580	2	1383286	4	4	3	3	3	3	3	1	4	4	3	3	4	3	6	4	5	2	3	3	2	2	2	2	6	3		2	4	2	2,53	3,00	5,00	34	#NIČ!
581	2	8546619	6	6	7	5	5	5	5	6	5	6	6	7	6	7	6	6	7	4	4	4	3	3	3	3	4	1		2	4	2	4,94	1,00	2,00	34	#NIČ!
582	2	8583234	7	7	7	7	6	4	4	4	5	3	3	4	2	6	6	2	6	2	5	4	3	2	2	1	4	1		2	2	2	3,88	1,00	2,00	34	#NIČ!
583	2	7276314	7	7	7	7	7	7	7	6	6	7	7	6	7	7	7	7	7	3	5	5	4	4	3	4	3	8		3	4	2	5,82	8,00	1,00	34	#NIČ!
584	2	631869	5	5	6	4	5	5	5	4	4	3	4	3	4	5	4	5	5	3	3	3	3	3	4	4	2	17		2	4	2	3,47	17,00	1,00	34	#NIČ!
585	2	1330401	4	6	7	5	7	6	6	6	5	7	4	4	4	5	4	2	5	2	5	5	2	2	3	2	4	1		1	5	2	4,12	1,00	2,00	34	#NIČ!
587	2	4756401	7	7	7	7	7	7	7	7	7	7	7	7	7	7	7	7	7	5	5	5	5	5	5	5	2	3		3	2	2	6,00	3,00	5,00	34	#NIČ!
588	2	9790801	6	7	6	6	5	6	5	4	5	6	4	6	6	6	6	4	7	5	4	4	4	5	5	5	6	17		1	5	1	4,59	17,00	1,00	34	#NIČ!
589	#NIČ!	4684801	#NIČ!	#NIČ!	#NIČ!	#NIČ!	#NIČ!	#NIČ!	#NIČ!	#NIČ!	#NIČ!	#NIČ!	#NIČ!	#NIČ!	#NIČ!	#NIČ!	#NIČ!	#NIČ!	#NIČ!	#NIČ!	#NIČ!	#NIČ!	#NIČ!	#NIČ!	#NIČ!	#NIČ!	#NIČ!	#NIČ!		#NIČ!	#NIČ!	#NIČ!	#NIČ!	#NIČ!	#NIČ!	34	#NIČ!
591	#NIČ!	6203606	#NIČ!	#NIČ!	#NIČ!	#NIČ!	#NIČ!	#NIČ!	#NIČ!	#NIČ!	#NIČ!	#NIČ!	#NIČ!	#NIČ!	#NIČ!	#NIČ!	#NIČ!	#NIČ!	#NIČ!	#NIČ!	#NIČ!	#NIČ!	#NIČ!	#NIČ!	#NIČ!	#NIČ!	#NIČ!	#NIČ!		#NIČ!	#NIČ!	#NIČ!	#NIČ!	#NIČ!	#NIČ!	34	#NIČ!
592	2	8863227	4	5	5	5	5	5	4	3	3	5	5	2	4	4	4	3	5	3	3	4	3	3	3	3	1	12		3	4	2	3,18	12,00	1,00	34	#NIČ!
593	2	4051602	7	7	7	7	7	7	7	6	7	7	7	6	7	7	6	6	7	3	4	4	3	3	3	3	3	7		2	3	2	5,76	7,00	4,00	34	#NIČ!
594	2	5705317	6	6	7	7	6	6	6	5	6	6	4	5	6	6	6	6	6	4	4	5	3	4	3	3	2	17		2	4	2	4,88	17,00	1,00	34	#NIČ!
595	2	1936904	4	5	5	4	3	4	3	3	3	3	3	3	3	5	4	1	4	2	3	2	2	2	2	2	2	1		3	4	2	2,53	1,00	2,00	34	#NIČ!
596	2	2568569	6	7	7	6	7	6	6	6	6	7	6	6	6	6	6	6	6	5	5	5	5	4	4	4	3	7		2	4	2	5,24	7,00	4,00	34	#NIČ!
597	2	7032132	4	5	6	6	6	6	6	3	5	5	5	5	5	5	5	1	2	3	5	5	3	3	4	3	6	3		2	4	2	3,71	3,00	5,00	34	#NIČ!
598	2	7454955	7	7	7	7	7	7	7	6	6	7	1	7	7	7	6	6	6	4	5	5	5	5	5	5	6	6		2	2	2	5,35	6,00	3,00	34	#NIČ!
599	2	6178377	6	7	7	7	7	7	6	6	7	7	7	6	6	7	6	5	7	5	5	5	5	5	5	5	3	1		2	3	2	5,53	1,00	2,00	34	#NIČ!
600	#NIČ!	8527021	#NIČ!	#NIČ!	#NIČ!	#NIČ!	#NIČ!	#NIČ!	#NIČ!	#NIČ!	#NIČ!	#NIČ!	#NIČ!	#NIČ!	#NIČ!	#NIČ!	#NIČ!	#NIČ!	#NIČ!	#NIČ!	#NIČ!	#NIČ!	#NIČ!	#NIČ!	#NIČ!	#NIČ!	#NIČ!	#NIČ!		#NIČ!	#NIČ!	#NIČ!	#NIČ!	#NIČ!	#NIČ!	34	#NIČ!
601	2	4099973	6	7	7	7	7	6	6	7	7	7	7	7	7	7	6	6	7	4	5	5	5	4	4	4	3	7		2	4	2	5,71	7,00	4,00	34	#NIČ!
602	2	4918804	5	5	5	4	4	7	4	4	5	3	5	5	4	5	4	3	5	3	4	5	3	4	3	3	3	6		1	4	2	3,53	6,00	3,00	34	#NIČ!
603	2	2294102	4	4	4	4	4	4	4	4	4	4	4	5	4	4	4	4	4	3	3	3	3	3	3	3	3	5		2	2	2	3,06	5,00	3,00	34	#NIČ!
604	2	6714097	6	7	7	7	6	6	6	6	7	7	6	6	6	7	6	6	6	5	5	5	5	5	5	5	3	1		2	2	2	5,35	1,00	2,00	34	#NIČ!
605	2	6688178	4	7	7	4	4	5	4	3	3	7	6	3	4	7	4	4	4	2	3	3	2	2	3	3	2	10		2	2	2	3,71	98,00	7,00	34	#NIČ!
606	2	3298599	6	6	7	6	6	7	6	6	6	6	6	7	7	7	6	6	7	4	4	4	4	4	3	3	8	#NIČ!		4	4	2	5,35	#NIČ!	1,00	34	#NIČ!

607	2	6428463	7	7	1	7	6	6	7	7	6	6	5	4	7	7	4	6	7	5	5	5	5	5	5	5	9	#NIČ!	uprava	2	4	1	4,88	98,00	7,00	26	1,00
608	#NIČ!	2246519	#NIČ!	#NIČ!	#NIČ!	#NIČ!	#NIČ!	#NIČ!	#NIČ!	#NIČ!	#NIČ!	#NIČ!	#NIČ!	#NIČ!	#NIČ!	#NIČ!	#NIČ!	#NIČ!	#NIČ!	#NIČ!	#NIČ!	#NIČ!	#NIČ!	#NIČ!	#NIČ!	#NIČ!	#NIČ!	#NIČ!		#NIČ!	#NIČ!	#NIČ!	#NIČ!	#NIČ!	#NIČ!	34	#NIČ!
609	2	1947207	6	7	7	6	6	6	6	6	6	6	6	7	5	7	6	6	6	4	4	4	4	4	5	4	3	1		2	2	2	5,18	1,00	2,00	34	#NIČ!
610	2	2996477	4	4	6	4	6	2	2	4	3	6	5	3	3	6	3	3	7	1	3	3	2	2	1	1	3	5		2	2	2	3,18	5,00	3,00	34	#NIČ!
611	2	9140765	6	6	6	6	6	4	5	6	4	7	6	6	6	6	6	4	4	3	5	5	4	4	5	4	1	6		1	4	2	4,53	6,00	3,00	34	#NIČ!
612	2	6714394	3	6	5	5	5	5	4	3	4	6	6	5	6	6	4	3	5	3	3	3	2	3	3	2	2	17		1	6	2	3,76	17,00	1,00	34	#NIČ!
613	2	6149676	4	5	6	4	4	4	4	3	3	4	4	3	4	7	4	4	5	3	3	3	3	3	2	2	3	1		2	2	2	3,24	1,00	2,00	34	#NIČ!
614	2	605199	4	7	6	4	4	4	4	3	4	3	5	4	4	5	5	2	5	2	4	1	2	3	3	2	3	1		1	5	2	3,29	1,00	2,00	34	#NIČ!
615	2	4576968	4	4	4	4	4	4	4	4	4	4	4	4	4	4	4	4	4	3	3	3	3	3	3	3	4	1		2	4	2	3,00	1,00	2,00	34	#NIČ!
616	2	1069244	6	7	6	6	4	4	4	4	5	5	5	5	5	4	4	2	5	2	4	4	3	3	3	3	4	#NIČ!	telefonij	4	3	2	3,76	98,00	7,00	25	1,00
617	2	1615315	7	7	7	7	7	7	7	7	6	7	6	7	6	7	6	7	7	5	5	5	5	5	5	5	1	1		1	5	2	5,76	1,00	2,00	34	#NIČ!
618	2	4868844	7	7	6	6	7	7	7	7	6	7	6	6	6	7	6	3	7	5	5	5	5	5	5	5	4	9		3	3	2	5,35	9,00	1,00	34	#NIČ!
619	#NIČ!	9498273	#NIČ!	#NIČ!	#NIČ!	#NIČ!	#NIČ!	#NIČ!	#NIČ!	#NIČ!	#NIČ!	#NIČ!	#NIČ!	#NIČ!	#NIČ!	#NIČ!	#NIČ!	#NIČ!	#NIČ!	#NIČ!	#NIČ!	#NIČ!	#NIČ!	#NIČ!	#NIČ!	#NIČ!	#NIČ!	#NIČ!		#NIČ!	#NIČ!	#NIČ!	#NIČ!	#NIČ!	#NIČ!	34	#NIČ!
620	2	2884834	4	7	7	7	4	6	5	4	6	6	6	4	3	7	4	3	7	5	4	4	3	4	4	3	3	3		2	4	2	4,29	3,00	5,00	34	#NIČ!
621	2	5929350	7	7	6	5	6	7	6	4	6	7	5	3	4	4	4	3	4	5	4	4	3	5	5	5	4	12		3	5	1	4,18	12,00	1,00	34	#NIČ!
622	#NIČ!	992250	#NIČ!	#NIČ!	#NIČ!	#NIČ!	#NIČ!	#NIČ!	#NIČ!	#NIČ!	#NIČ!	#NIČ!	#NIČ!	#NIČ!	#NIČ!	#NIČ!	#NIČ!	#NIČ!	#NIČ!	#NIČ!	#NIČ!	#NIČ!	#NIČ!	#NIČ!	#NIČ!	#NIČ!	#NIČ!	#NIČ!		#NIČ!	#NIČ!	#NIČ!	#NIČ!	#NIČ!	#NIČ!	34	#NIČ!
623	2	7173200	4	5	6	4	4	6	3	3	4	5	4	4	3	3	4	3	3	3	3	3	3	3	3	3	4	4		2	3	2	3,00	4,00	6,00	34	#NIČ!
624	#NIČ!	2889249	#NIČ!	#NIČ!	#NIČ!	#NIČ!	#NIČ!	#NIČ!	#NIČ!	#NIČ!	#NIČ!	#NIČ!	#NIČ!	#NIČ!	#NIČ!	#NIČ!	#NIČ!	#NIČ!	#NIČ!	#NIČ!	#NIČ!	#NIČ!	#NIČ!	#NIČ!	#NIČ!	#NIČ!	#NIČ!	#NIČ!		#NIČ!	#NIČ!	#NIČ!	#NIČ!	#NIČ!	#NIČ!	34	#NIČ!
625	2	2926645	5	6	6	6	5	3	4	5	4	7	6	6	6	6	5	6	5	2	3	4	4	3	3	3	1	7		2	3	1	4,35	7,00	4,00	34	#NIČ!
626	#NIČ!	5965479	#NIČ!	#NIČ!	#NIČ!	#NIČ!	#NIČ!	#NIČ!	#NIČ!	#NIČ!	#NIČ!	#NIČ!	#NIČ!	#NIČ!	#NIČ!	#NIČ!	#NIČ!	#NIČ!	#NIČ!	#NIČ!	#NIČ!	#NIČ!	#NIČ!	#NIČ!	#NIČ!	#NIČ!	#NIČ!	#NIČ!		#NIČ!	#NIČ!	#NIČ!	#NIČ!	#NIČ!	#NIČ!	34	#NIČ!
627	2	1047460	6	7	7	7	7	6	7	6	6	7	5	5	6	7	6	5	6	4	5	5	5	4	3	3	5	1		2	2	2	5,24	1,00	2,00	34	#NIČ!
628	2	7340862	7	7	7	7	7	7	7	6	7	7	7	7	7	7	7	7	7	4	5	5	5	5	5	5	8	#NIČ!	IRROZ	1	7	1	5,94	98,00	7,00	13	1,00
629	#NIČ!	3561933	#NIČ!	#NIČ!	#NIČ!	#NIČ!	#NIČ!	#NIČ!	#NIČ!	#NIČ!	#NIČ!	#NIČ!	#NIČ!	#NIČ!	#NIČ!	#NIČ!	#NIČ!	#NIČ!	#NIČ!	#NIČ!	#NIČ!	#NIČ!	#NIČ!	#NIČ!	#NIČ!	#NIČ!	#NIČ!	#NIČ!		#NIČ!	#NIČ!	#NIČ!	#NIČ!	#NIČ!	#NIČ!	34	#NIČ!
630	2	5787077	5	6	6	6	5	5	5	4	5	6	5	4	5	5	6	3	7	3	3	4	4	3	4	3	3	6		2	4	2	4,18	6,00	3,00	34	#NIČ!
631	2	8249588	7	6	6	5	6	6	6	6	6	6	6	6	6	6	6	6	6	4	4	4	3	4	4	4	3	#NIČ!	vrzdržev	4	2	2	5,00	98,00	7,00	32	1,00
632	2	3886708	6	6	6	6	6	6	6	6	6	6	6	6	6	6	6	6	6	3	4	5	5	5	5	5	5	1		1	5	1	5,00	1,00	2,00	34	#NIČ!
633	2	4684776	6	7	7	6	6	7	6	7	6	6	7	7	7	7	6	6	6	3	5	4	4	4	4	4	2	18		3	2	2	5,47	18,00	3,00	34	#NIČ!
634	2	1763757	7	6	7	6	6	7	6	7	6	6	7	6	6	6	6	6	7	4	5	3	3	3	3	3	3	7		2	4	2	5,35	7,00	4,00	34	#NIČ!
635	2	4764459	6	6	5	5	5	5	5	5	6	6	5	4	6	6	6	4	6	2	5	5	5	3	5	5	3	1		2	2	2	4,35	1,00	2,00	34	#NIČ!
636	2	8531022	5	6	6	5	5	5	6	5	5	4	4	5	6	5	6	4	5	3	5	5	4	4	5	5	3	2		1	5	1	4,12	2,00	5,00	34	#NIČ!
637	2	8361733	4	7	6	6	6	5	6	5	6	6	6	5	5	7	6	4	6	3	3	3	3	3	3	3	1	2		2	2	2	4,65	2,00	5,00	34	#NIČ!
638	2	6215598	5	7	6	6	6	5	5	5	5	7	6	5	5	6	4	2	4	4	5	3	3	4	4	4	2	8		3	4	2	4,24	8,00	1,00	34	#NIČ!
639	2	5992793	4	4	5	4	4	5	4	3	3	4	5	4	4	5	4	4	5	3	3	3	2	3	3	3	2	1		1	3	2	3,18	1,00	2,00	34	#NIČ!
640	2	1317172	6	7	7	7	7	7	7	6	6	7	7	6	6	7	7	1	7	5	5	5	5	5	5	5	3	8		3	4	2	5,35	8,00	1,00	34	#NIČ!
641	2	8607479	7	7	7	6	6	7	7	7	7	7	6	7	7	7	7	3	6	2	3	3	3	2	3	2	2	10		2	2	1	5,53	98,00	1,00	34	#NIČ!
642	2	9085883	6	6	7	6	6	7	6	6	6	6	7	6	6	6	6	6	5	3	5	5	3	4	4	5	6	1		1	5	2	5,12	1,00	2,00	34	#NIČ!
643	2	9606974	6	7	6	5	5	4	5	5	6	5	6	5	5	5	5	4	5	4	3	3	3	3	2	3	4	12		3	4	2	4,24	12,00	1,00	34	#NIČ!
644	2	777225	6	6	6	6	5	5	4	4	4	6	5	5	5	5	5	4	4	3	3	3	2	3	3	3	5	1		1	4	2	4,00	1,00	2,00	34	#NIČ!
645	2	5065203	6	7	6	6	6	7	6	6	6	6	7	7	7	7	6	7	7	4	4	5	5	3	3	4	2	6		1	4	2	5,47	6,00	3,00	34	#NIČ!
646	2	2994339	7	7	7	6	6	6	6	6	6	7	6	6	6	6	6	5	7	5	5	5	5	4	4	4	4	11		1	5	1	5,24	11,00	1,00	34	#NIČ!
647	2	9776085	7	7	7	7	7	7	7	7	7	7	7	7	7	7	7	7	7	5	5	5	5	5	5	5	2	#NIČ!	SIM	2	2	2	6,00	98,00	7,00	17	1,00
648	2	9897411	6	6	7	6	4	3	3	2	5	3	5	6	7	4	2	3	4	2	3	3	3	2	3	3	6	15		3	4	2	3,47	15,00	1,00	34	#NIČ!
649	2	158798	6	7	7	6	6	6	6	6	2	6	6	6	6	6	6	4	6	4	4	4	4	4	3	3	6	5		1	4	2	4,76	5,00	3,00	34	#NIČ!
650	2	1101756	7	7	7	7	6	6	6	6	6	6	6	6	6	6	6	6	6	4	4	3	3	3	3	3	3	1		1	5	2	5,24	1,00	2,00	34	#NIČ!
651	#NIČ!	5032388	#NIČ!	#NIČ!	#NIČ!	#NIČ!	#NIČ!	#NIČ!	#NIČ!	#NIČ!	#NIČ!	#NIČ!	#NIČ!	#NIČ!	#NIČ!	#NIČ!	#NIČ!	#NIČ!	#NIČ!	#NIČ!	#NIČ!	#NIČ!	#NIČ!	#NIČ!	#NIČ!	#NIČ!	#NIČ!	#NIČ!		#NIČ!	#NIČ!	#NIČ!	#NIČ!	#NIČ!	#NIČ!	34	#NIČ!
652	2	1856672	7	7	7	6	6	7	7	7	7	6	3	7	7	7	7	2	7	4	5	5	3	4	5	5	3	1		1	5	2	5,29	1,00	2,00	34	#NIČ!
653	2	4186198	6	7	5	5	7	6	7	4	6	7	7	7	7	7	6	3	4	3	3	4	3	3	3	3	6	3		2	1	2	4,94	3,00	5,00	34	#NIČ!
654	2	5360974	6	6	6	5	6	7	6	6	5	6	6	6	6	6	6	5	6	3	4	4	2	2	3	3	3	1		1	5	2	4,88	1,00	2,00	34	#NIČ!
655	2	4246276	7	7	6	7	7	7	7	6	7	7	6	7	7	7	7	6	7	5	5	5	4	5	5	5	7	#NIČ!	Snmp	2	2	2	5,76	98,00	7,00	20	1,00
656	#NIČ!	5148460	#NIČ!	#NIČ!	#NIČ!	#NIČ!	#NIČ!	#NIČ!	#NIČ!	#NIČ!	#NIČ!	#NIČ!	#NIČ!	#NIČ!	#NIČ!	#NIČ!	#NIČ!	#NIČ!	#NIČ!	#NIČ!	#NIČ!	#NIČ!	#NIČ!	#NIČ!	#NIČ!	#NIČ!	#NIČ!	#NIČ!		#NIČ!	#NIČ!	#NIČ!	#NIČ!	#NIČ!	#NIČ!	34	#NIČ!
657	2	3003469	4	6	6	5	4	5	3	2	4	4	3	4	4	3	2	1	5	3	4	5	4	3	5	3	7	#NIČ!	snmp	1	5	2	2,82	98,00	7,00	19	1,00
658	2	9571968	6	7	5	7	7	7	7	6	7	7	7	7	7	7	7	7	7	5	5	5	5	5	5	5	2	1		2	2	2	5,76	1,00	2,00	34	#NIČ!
659	2	8849433	6	6	6	6	6	6	5	5	6	6	6	6	6	6	6	4	4	4	5	3	3	4	4	3	6	6		1	4	2	4,65	6,00	3,00	34	#NIČ!
660	2	5531262	6	6	6	6	5	6	6	5	5	5	5	6	6	6	6	5	6	3	3	4	3	3	4	4	4	14		2	4	2	4,65	14,00	1,00	34	#NIČ!

661	2	1108010	6	6	6	6	6	6	6	6	6	6	6	6	6	6	6	6	6	2	3	3	3	3	3	3	3	7		2		2	2	5,00	7,00	4,00	34	#NIČ!
662	2	8946266	6	6	7	6	6	7	6	6	6	7	6	6	6	7	6	6	6	2	5	5	5	5	5	5	3	5		2		4	2	5,24	5,00	3,00	34	#NIČ!
663	#NIČ!	1407301	#NIČ!	#NIČ!	#NIČ!	#NIČ!	#NIČ!	#NIČ!	#NIČ!	#NIČ!	#NIČ!	#NIČ!	#NIČ!	#NIČ!	#NIČ!	#NIČ!	#NIČ!	#NIČ!	#NIČ!	#NIČ!	#NIČ!	#NIČ!	#NIČ!	#NIČ!	#NIČ!	#NIČ!	#NIČ!	#NIČ!		#NIČ!		#NIČ!	#NIČ!	#NIČ!	#NIČ!	#NIČ!	34	#NIČ!
664	2	197707	6	7	7	7	7	6	7	7	6	7	7	7	7	6	7	6	6	4	4	4	4	3	5	4	3	7		2		4	2	5,65	7,00	4,00	34	#NIČ!
665	2	6766631	6	7	7	6	6	7	6	6	6	7	7	7	7	7	6	5	7	4	4	4	4	4	3	3	4	7		2		4	2	5,47	7,00	4,00	34	#NIČ!
666	#NIČ!	3240037	#NIČ!	#NIČ!	#NIČ!	#NIČ!	#NIČ!	#NIČ!	#NIČ!	#NIČ!	#NIČ!	#NIČ!	#NIČ!	#NIČ!	#NIČ!	#NIČ!	#NIČ!	#NIČ!	#NIČ!	#NIČ!	#NIČ!	#NIČ!	#NIČ!	#NIČ!	#NIČ!	#NIČ!	#NIČ!	#NIČ!		#NIČ!		#NIČ!	#NIČ!	#NIČ!	#NIČ!	#NIČ!	34	#NIČ!
667	#NIČ!	5900290	#NIČ!	#NIČ!	#NIČ!	#NIČ!	#NIČ!	#NIČ!	#NIČ!	#NIČ!	#NIČ!	#NIČ!	#NIČ!	#NIČ!	#NIČ!	#NIČ!	#NIČ!	#NIČ!	#NIČ!	#NIČ!	#NIČ!	#NIČ!	#NIČ!	#NIČ!	#NIČ!	#NIČ!	#NIČ!	#NIČ!		#NIČ!		#NIČ!	#NIČ!	#NIČ!	#NIČ!	#NIČ!	34	#NIČ!
668	2	9781341	5	5	6	6	6	4	5	5	5	5	5	5	5	5	5	5	5	4	5	4	4	4	4	4	3	7		2		4	2	4,12	7,00	4,00	34	#NIČ!
669	2	1205835	3	4	4	4	4	4	4	4	4	4	4	4	4	4	4	4	4	3	3	3	3	3	3	3	3	7		2		2	2	2,94	7,00	4,00	34	#NIČ!
670	2	6685152	7	7	7	7	7	7	7	7	7	7	7	7	7	7	7	7	7	4	5	5	4	5	5	4	3	7		2		2	2	6,00	7,00	4,00	34	#NIČ!
671	2	9808254	3	5	5	4	2	6	4	3	2	4	5	4	4	6	6	6	7	3	5	4	2	2	4	1	2	1		1		5	2	3,47	1,00	2,00	34	#NIČ!
672	2	8985814	5	7	5	4	5	3	5	3	5	6	4	2	4	5	7	5	6	1	5	5	2	2	2	3	5	8		3		4	2	3,76	8,00	1,00	34	#NIČ!
673	2	5504309	7	6	7	6	5	6	5	4	4	5	4	4	5	7	4	4	7	2	5	4	3	2	3	3	8	#NIČ!	uprava	4		2	2	4,29	98,00	7,00	26	1,00
674	2	564105	6	7	7	6	7	7	7	7	6	7	7	5	7	7	7	6	7	5	5	5	5	5	5	5	9	#NIČ!	SIM cen	2		4	2	5,65	98,00	7,00	18	1,00
675	2	6307599	5	7	6	6	6	4	5	4	4	6	6	4	5	5	5	4	5	3	4	4	3	3	3	3	7	#NIČ!		1		5	2	4,12	#NIČ!	#NIČ!	34	#NIČ!
676	2	9845680	6	7	7	7	7	6	7	2	6	7	7	7	7	7	6	7	7	2	4	5	5	4	5	4	1	2		2		3	2	5,47	2,00	5,00	34	#NIČ!
677	2	305604	4	6	6	6	6	6	6	6	6	5	4	6	6	6	6	4	6	4	4	4	4	4	3	3	2	19		2		3	2	4,59	98,00	1,00	34	#NIČ!
678	2	1990981	6	7	6	5	5	5	6	6	6	7	7	6	6	6	4	5	5	3	4	5	3	3	4	4	7	#NIČ!	Urgenca	1		6	2	4,76	98,00	7,00	29	1,00
679	2	9038093	3	4	4	4	3	4	4	4	4	3	3	3	4	3	2	2	2	3	3	3	3	2	2	1	4	1		1		5	2	2,29	1,00	2,00	34	#NIČ!
680	2	9217523	4	5	5	4	5	5	5	5	5	5	4	5	5	5	4	1	5	3	4	4	3	3	4	4	4	4		2		2	2	3,53	4,00	6,00	34	#NIČ!
681	2	8973335	7	7	7	7	7	7	7	7	7	7	7	7	7	7	7	7	7	5	5	5	5	5	5	5	6	14		3		5	2	6,00	14,00	1,00	34	#NIČ!
682	2	7214108	7	7	7	7	7	7	7	7	7	7	7	7	7	7	7	7	7	5	5	5	5	5	5	5	6	5		1		4	2	6,00	5,00	3,00	34	#NIČ!
683	#NIČ!	9150535	#NIČ!	#NIČ!	#NIČ!	#NIČ!	#NIČ!	#NIČ!	#NIČ!	#NIČ!	#NIČ!	#NIČ!	#NIČ!	#NIČ!	#NIČ!	#NIČ!	#NIČ!	#NIČ!	#NIČ!	#NIČ!	#NIČ!	#NIČ!	#NIČ!	#NIČ!	#NIČ!	#NIČ!	#NIČ!	#NIČ!		#NIČ!		#NIČ!	#NIČ!	#NIČ!	#NIČ!	#NIČ!	34	#NIČ!
684	2	4110352	3	4	7	1	4	7	7	4	7	7	3	4	5	5	6	6	3	3	4	4	2	3	3	2	3	1		2		2	2	3,88	1,00	2,00	34	#NIČ!
685	2	3100156	6	6	6	6	6	6	6	6	6	6	6	6	6	6	6	5	6	4	4	4	4	4	4	3	6	2		1		6	2	4,94	2,00	5,00	34	#NIČ!
686	2	3169723	7	7	7	6	6	6	6	6	6	7	6	5	6	6	6	5	6	3	3	3	3	2	3	3	1	14		#NIČ!	prof.z	4	2	5,12	14,00	1,00	34	#NIČ!
687	2	6548149	6	7	6	6	6	6	6	6	5	6	6	5	6	6	6	6	6	3	4	4	4	4	4	4	5	1		1		5	2	4,94	1,00	2,00	34	#NIČ!
688	2	3231575	6	7	7	7	7	6	7	7	7	7	7	7	7	7	6	5	7	3	5	5	4	3	3	3	3	6		3		2	2	5,71	6,00	3,00	34	#NIČ!
689	2	6513430	7	7	7	7	7	7	7	7	7	7	7	7	7	7	7	7	7	5	5	5	5	5	5	5	3	23		1		5	2	6,00	98,00	1,00	34	#NIČ!
690	2	2872426	5	6	7	5	6	6	5	6	6	6	6	6	6	6	6	5	6	1	5	4	3	4	5	3	6	1		2		4	2	4,82	1,00	2,00	34	#NIČ!
691	2	4821838	1	5	5	6	5	6	6	5	5	5	5	5	4	5	4	3	4	3	3	3	3	3	3	3	1	2		2		3	2	3,65	2,00	5,00	34	#NIČ!
692	2	5491914	3	4	7	6	6	7	6	6	7	7	7	7	7	7	7	7	7	5	5	5	5	5	5	5	2	6		1		4	2	5,35	6,00	3,00	34	#NIČ!
693	2	2994056	4	7	5	4	4	4	4	3	4	5	4	5	4	4	5	1	5	2	3	2	1	1	1	1	6	6		1		4	2	3,24	6,00	3,00	34	#NIČ!
694	2	8494539	6	7	7	7	7	7	7	6	7	6	7	7	7	7	6	6	7	4	5	5	5	5	5	5	1	6		1		4	2	5,71	6,00	3,00	34	#NIČ!
695	2	3490527	7	7	6	7	7	7	7	6	6	7	7	7	7	7	6	7	7	5	5	5	5	5	5	5	1	6		2		2	2	5,76	6,00	3,00	34	#NIČ!
696	2	1969017	7	5	7	4	5	7	3	4	4	5	3	3	2	5	3	2	6	2	3	3	2	2	2	1	6	9		2		4	2	3,41	9,00	1,00	34	#NIČ!
697	2	9373503	6	6	6	6	6	6	6	6	6	6	6	6	6	6	6	6	6	3	3	3	3	3	3	3	2	2		1		5	2	5,00	2,00	5,00	34	#NIČ!
698	2	960467	6	7	7	7	7	7	7	6	6	7	7	7	7	7	6	6	7	4	5	5	4	5	4	4	1	18		3		2	1	5,71	18,00	3,00	34	#NIČ!
699	2	6681824	7	7	6	6	6	6	6	6	6	6	6	6	6	6	6	6	5	4	4	4	4	4	4	4	3	5		2		2	2	5,06	5,00	3,00	34	#NIČ!
700	2	527719	4	7	7	4	4	6	5	4	5	5	4	6	6	7	6	2	6	3	3	3	3	3	2	2	3	#NIČ!		4		2	2	4,18	#NIČ!	#NIČ!	34	#NIČ!
701	2	2593123	7	7	6	6	6	7	5	6	6	5	6	7	7	6	5	4	7	1	4	5	4	3	4	4	6	6		1		5	1	5,06	6,00	3,00	34	#NIČ!
702	#NIČ!	1382459	#NIČ!	#NIČ!	#NIČ!	#NIČ!	#NIČ!	#NIČ!	#NIČ!	#NIČ!	#NIČ!	#NIČ!	#NIČ!	#NIČ!	#NIČ!	#NIČ!	#NIČ!	#NIČ!	#NIČ!	#NIČ!	#NIČ!	#NIČ!	#NIČ!	#NIČ!	#NIČ!	#NIČ!	#NIČ!	#NIČ!		#NIČ!		#NIČ!	#NIČ!	#NIČ!	#NIČ!	#NIČ!	34	#NIČ!
703	2	9132928	5	7	7	6	5	7	4	4	4	6	7	7	5	6	5	3	7	4	4	4	3	3	2	3	8	#NIČ!		4		3	2	4,59	#NIČ!	#NIČ!	34	#NIČ!
704	2	1517262	6	6	7	7	7	7	7	7	7	7	7	7	7	7	7	6	7	3	4	4	2	4	3	3	6	1		1		5	2	5,82	1,00	2,00	34	#NIČ!
705	2	187527	4	5	6	4	4	5	4	3	4	5	6	3	4	4	1	3	6	2	3	2	2	3	3	2	1	8		3		4	2	3,18	8,00	1,00	34	#NIČ!
706	2	6385850	3	4	5	2	3	3	3	2	3	3	2	2	2	3	3	3	6	3	3	4	4	4	3	3	2	13		2		2	2	2,06	98,00	6,00	34	#NIČ!
707	2	1366667	7	7	7	6	7	7	6	7	6	7	6	7	7	7	7	6	7	2	5	4	3	3	2	2	6	5		1		4	2	5,71	5,00	3,00	34	#NIČ!
708	2	7675785	4	7	7	7	7	7	7	6	6	7	7	7	7	7	6	2	7	3	3	3	3	3	3	3	3	7		2		4	2	5,35	7,00	4,00	34	#NIČ!
709	2	4278924	7	7	7	7	7	7	7	7	7	7	7	7	7	7	7	7	7	5	5	5	5	5	5	5	2	9		1		5	2	6,00	9,00	1,00	34	#NIČ!
710	2	8367268	6	7	7	7	7	7	6	6	7	7	6	7	6	6	6	6	6	3	3	5	4	3	4	4	1	3		1		5	1	5,47	3,00	5,00	34	#NIČ!
711	2	8999567	5	5	5	4	5	5	5	6	5	6	5	5	5	6	5	6	5	4	3	3	3	3	3	3	4	7		2		4	2	4,18	7,00	4,00	34	#NIČ!
712	2	9896033	5	5	5	5	4	4	5	5	4	5	4	4	4	4	4	2	5	2	4	4	3	3	3	3	5	12		3		2	2	3,35	12,00	1,00	34	#NIČ!
713	2	2481465	6	7	7	6	6	7	6	6	7	7	7	6	6	7	6	6	7	4	5	4	4	4	2	3	2	5		2		2	2	5,47	5,00	3,00	34	#NIČ!
714	2	2719224	6	6	7	6	4	6	4	5	4	4	7	7	7	6	6	5	7	2	3	3	1	3	4	4	1	15		#NIČ!		5	2	4,71	15,00	1,00	34	#NIČ!

715	2	6151725	6	7	6	6	6	6	7	6	7	7	6	7	7	7	6	6	7	3	5	5	4	4	4	3	1	1	1	5	2	5,47	1,00	2,00	34	#NIČ!
716	2	2600953	5	7	7	7	5	4	6	5	4	7	7	4	7	7	5	5	7	3	4	4	3	3	4	3	2	#NIČ!	4	2	2	4,82	#NIČ!	#NIČ!	34	#NIČ!
717	#NIČ!	4549592	#NIČ!	#NIČ!	#NIČ!	#NIČ!	#NIČ!	#NIČ!	#NIČ!	#NIČ!	#NIČ!	#NIČ!	#NIČ!	#NIČ!	#NIČ!	#NIČ!	#NIČ!	#NIČ!	#NIČ!	#NIČ!	#NIČ!	#NIČ!	#NIČ!	#NIČ!	#NIČ!	#NIČ!	#NIČ!	#NIČ!	#NIČ!	#NIČ!	#NIČ!	#NIČ!	#NIČ!	#NIČ!	34	#NIČ!
718	#NIČ!	4945102	#NIČ!	#NIČ!	#NIČ!	#NIČ!	#NIČ!	#NIČ!	#NIČ!	#NIČ!	#NIČ!	#NIČ!	#NIČ!	#NIČ!	#NIČ!	#NIČ!	#NIČ!	#NIČ!	#NIČ!	#NIČ!	#NIČ!	#NIČ!	#NIČ!	#NIČ!	#NIČ!	#NIČ!	#NIČ!	#NIČ!	#NIČ!	#NIČ!	#NIČ!	#NIČ!	#NIČ!	#NIČ!	34	#NIČ!
719	2	1076734	6	6	7	6	6	4	5	5	5	6	4	5	7	6	5	3	4	3	4	5	5	4	4	3	3	5	1	4	2	4,29	5,00	3,00	34	#NIČ!
720	2	548364	6	7	6	7	6	6	6	6	5	7	6	7	7	7	6	5	7	4	4	4	4	4	3	2	4	1	1	5	2	5,29	1,00	2,00	34	#NIČ!
721	2	9511618	7	7	7	7	7	7	7	7	7	7	7	7	7	7	7	7	7	5	5	5	5	5	5	5	6	12	3	2	2	6,00	12,00	1,00	34	#NIČ!
722	2	5912997	4	4	4	4	4	4	4	4	4	4	4	4	4	4	4	4	4	3	3	3	3	3	3	3	6	12	3	5	2	3,00	12,00	1,00	34	#NIČ!
723	2	1030131	6	6	6	6	6	6	6	5	5	6	6	6	6	6	5	6	6	4	4	4	4	4	4	4	2	5	1	7	2	4,82	5,00	3,00	34	#NIČ!
724	2	7411666	6	6	6	4	4	4	5	4	4	4	5	4	5	4	4	3	4	2	3	4	3	3	1	1	3	5	1	5	2	3,47	5,00	3,00	34	#NIČ!
725	2	3967936	6	7	6	6	6	6	6	6	7	6	6	6	6	6	6	5	7	1	4	5	4	4	4	3	6	6	1	4	2	5,12	6,00	3,00	34	#NIČ!
726	2	7604682	7	7	7	7	7	7	7	7	7	7	7	7	7	7	7	6	7	3	5	5	4	5	3	3	2	4	2	4	2	5,94	4,00	6,00	34	#NIČ!
727	2	6119601	7	7	7	7	7	7	7	7	7	7	7	7	7	7	7	5	7	5	5	5	3	3	2	3	2	6	1	4	2	5,88	6,00	3,00	34	#NIČ!
728	2	7783962	6	7	7	7	7	7	7	6	7	7	7	7	7	7	6	7	7	5	4	4	4	4	4	4	1	1	2	2	2	5,82	1,00	2,00	34	#NIČ!
729	2	561006	7	7	7	7	7	7	7	7	7	7	7	7	7	7	7	7	7	5	5	5	5	5	5	5	3	18	3	1	2	6,00	18,00	3,00	34	#NIČ!
730	2	9453143	4	7	7	4	5	4	4	4	4	7	7	3	7	7	3	4	7	3	3	3	3	2	1	2	5	6	1	4	2	4,18	6,00	3,00	34	#NIČ!
731	2	5582697	5	6	7	6	5	7	5	6	7	6	5	6	6	6	5	6	7	3	4	5	4	4	4	4	3	1	1	5	2	4,94	1,00	2,00	34	#NIČ!
732	2	9554057	7	7	7	7	7	7	7	7	7	7	7	7	7	7	7	7	7	4	4	4	4	4	3	3	4	5	1	4	2	6,00	5,00	3,00	34	#NIČ!
733	2	1022193	6	6	7	6	6	6	6	6	6	6	5	5	6	7	5	5	6	4	4	5	4	3	4	4	6	12	5	5	1	4,88	12,00	1,00	34	#NIČ!
734	2	6448797	3	7	6	5	6	4	6	6	6	7	6	6	6	7	5	4	5	5	5	5	5	5	4	4	6	20	2	2	2	4,59	20,00	1,00	34	#NIČ!
735	#NIČ!	9177405	#NIČ!	#NIČ!	#NIČ!	#NIČ!	#NIČ!	#NIČ!	#NIČ!	#NIČ!	#NIČ!	#NIČ!	#NIČ!	#NIČ!	#NIČ!	#NIČ!	#NIČ!	#NIČ!	#NIČ!	#NIČ!	#NIČ!	#NIČ!	#NIČ!	#NIČ!	#NIČ!	#NIČ!	#NIČ!	#NIČ!	#NIČ!	#NIČ!	#NIČ!	#NIČ!	#NIČ!	#NIČ!	34	#NIČ!
736	2	6540635	6	7	6	6	6	5	6	6	6	6	6	6	6	6	6	5	6	3	4	4	4	4	4	4	2	22	1	5	2	4,94	98,00	1,00	34	#NIČ!
737	2	5170958	7	7	7	7	7	7	7	7	7	7	7	7	7	7	7	7	7	5	5	5	5	5	5	5	6	14	2	3	2	6,00	14,00	1,00	34	#NIČ!
738	2	6232888	7	7	7	7	7	7	7	7	7	7	7	7	7	7	7	7	7	5	5	5	5	5	5	5	1	28	2	2	2	6,00	98,00	1,00	34	#NIČ!
739	2	5651566	5	7	5	6	4	6	3	2	4	7	7	7	7	5	5	5	7	3	5	5	4	4	5	4	7	1	1	5	2	4,41	1,00	2,00	34	#NIČ!
740	2	9559165	7	7	7	7	5	7	7	7	7	4	7	7	7	7	7	6	7	4	3	5	4	3	3	3	6	3	1	5	2	5,65	3,00	5,00	34	#NIČ!
741	2	841129	7	7	6	6	5	4	5	6	4	7	6	5	5	7	5	3	7	2	4	4	3	2	3	3	6	8	3	4	2	4,59	8,00	1,00	34	#NIČ!
